# Importance of details in food descriptions in estimating population nutrient intake distributions

**DOI:** 10.1186/s12937-019-0443-5

**Published:** 2019-03-15

**Authors:** Liangzi Zhang, Anouk Geelen, Hendriek C. Boshuizen, José Ferreira, Marga C. Ocké

**Affiliations:** 1 Division of Human Nutrition, Wageningen University, Wageningen, the Netherlands; 2 National Institute for Public Health and the Environment (RIVM), Bilthoven, the Netherlands

**Keywords:** 24-h recall, Food consumption survey, Food descriptions, Dietary assessment, Population nutrient intake distributions, Variable importance measure

## Abstract

**Background:**

National food consumption surveys are important policy instruments that could monitor food consumption of a certain population. To be used for multiple purposes, this type of survey usually collects comprehensive food information using dietary assessment methods like 24-h dietary recalls (24HRs). However, the collection and handling of such detailed information require tremendous efforts. We aimed to improve the efficiency of data collection and handling in 24HRs, by identifying less important characteristics of food descriptions (facets) and assessing the impact of disregarding them on energy and nutrient intake distributions.

**Methods:**

In the Dutch National Food Consumption Survey 2007–2010, food consumption data were collected through interviewer-administered 24HRs using GloboDiet software in 3819 persons. Interviewers asked participants about the characteristics of each food item according to applicable facets. Food consumption data were subsequently linked to the food composition database. The importance of facets for predicting energy and each of the 33 nutrients was estimated using the random forest algorithm. Then a simulation study was performed to determine the influence of deleting less important facets on population nutrient intake distributions.

**Results:**

We identified 35% facets as unimportant and deleted them from the total food consumption database. The majority (79.4%) of the percent difference between percentile estimates of the population nutrient intake distributions before and after facet deletion ranged from 0 to 1%, while 20% cases ranged from 1 to 5% and 0.6% cases more than 10%.

**Conclusion:**

We concluded that our procedure was successful in identifying less important food descriptions in estimating population nutrient intake distributions. The reduction in food descriptions has the potential to reduce the time needed for conducting interviews and data handling while maintaining the data quality of the survey.

**Electronic supplementary material:**

The online version of this article (10.1186/s12937-019-0443-5) contains supplementary material, which is available to authorized users.

## Background

National food consumption surveys are essential policy instruments and have been carried out successively in many countries [1, 2]. They serve many purposes, such as identifying nutrient inadequacies at the population level, assessing the risk of hazardous substances, and developing dietary guidelines [1, 3].

The 24-h dietary recall (24HR) has been frequently used as the primary dietary assessment method for collecting national food consumption data [4, 5]. As an open-ended and retrospective method, 24HR is less likely to alter diet behaviour and has a lower literacy requirement for the participants than food records [6, 7]. Traditionally, interviewers collect information about the foods consumed during the preceding day or the previous 24 h by triggering the participant’s memory using different cues to increase the completeness of the survey [8]. This method collects sufficient food consumption data but has a long interview duration and a rather complicated data handling procedure [9, 10].

With the advent of computers, several comprehensive dietary assessment protocols have been incorporated into computer-assisted 24HR interview software used in large-scale studies [5, 11, 12]. These protocols standardize the dietary data collection procedure and help the respondents recall their food intake to the maximum extent [13]. Examples include the Automated Multiple-Pass Method (AMPM), developed by the U.S. Department of Agriculture (USDA) to conduct the dietary interview for the National Health and Nutrition Examination Survey [14]. In Europe, the International Agency for Research on Cancer (IARC) has developed the menu-driven 24HR software GloboDiet (previously known as EPIC-Soft), which was validated to be used in food consumption surveys in European countries [15, 16].

In the multiple-pass protocol of GloboDiet, the most time-consuming step is the collection of detailed information on each consumed food (i.e., food description). Details of each food item are collected through prompt windows for facets, which represent various characteristics of food, such as fat content, cooking method, and brand name. The predefined answers to the facet questions were called descriptors, such as full fat, semi-skimmed, and skimmed [17]. The use of facets and descriptors standardize the interview among different interviewers and characterize the consumed foods in aspects relevant for the study purposes, such as the content of nutrients and potentially hazardous chemicals [18–20].

Although applying a large number of facets and descriptors provides a high level of detail, the duration, and cost of the survey rise accordingly [7]. Specifically, the interviewers ask more questions during the interview, and the dieticians have to link all new food-descriptor combinations to the food composition database manually after the interview [10, 21, 22]. Furthermore, some food characteristics that require reading food labels (e.g., fortification) or knowledge about the preparation of the food (e.g., type of fat used) are difficult for many of the participants [1, 23]. Therefore, an investigation is needed on whether a reduction in food characteristics could improve the cost-effectiveness while maintaining data quality of the survey.

The current study aims to evaluate facet importance in predicting nutrient contents of foods, the impact on population nutrient intake distributions and the time saved after deleting less important facets from the data collection procedure.

## Methods

### Data collection

In the Netherlands, Dutch National Food Consumption Surveys (DNFCS) monitors the food consumption of the general Dutch population. The data used in this study came from the DNFCS performed from 2007 to 2010 on the diet of children and adults aged 7 to 69 years. Study design, recruitment, and results have been described elsewhere [24]. Subjects were excluded if they were pregnant, lactating, institutionalized or did not speak adequate Dutch. In total, 3819 participants (69%) were qualified and responded to the survey.

Dietary intake of participants was collected through two 24HRs on non-consecutive days with 2–6 weeks in between. Trained dieticians conducted the 24HRs for 2522 persons aged 16 and older through telephone interviews. The 24HRs for 1297 children between 7 to 15 years old were collected by face-to-face interviews with the presence of their caretakers during home visits. All interviews were conducted following the same data collection and handling protocol.

During both face-to-face and telephone 24HR interview, dieticians used the multi-step computer-based interview software GloboDiet to guide the interview and to enter the data in the computer. The average time needed to complete one face-to-face 24HR interview and one telephone interview was 41 min and 46 min, respectively. The GloboDiet interview consists of the following five steps: 1. Collection of the general information, 2. Listing of foods and recipes consumed throughout the day, 3. Specification of details of foods by choosing descriptors of relevant facets and consumed amounts, 4. Quality check of inaccurate input, and 5. Dietary supplement intake [15]. The collection of details in step three took about 15 min. IARC provided common facets and descriptors for countries that used Globodiet as their data collection software. The actual selection of facets and descriptors could be adjusted according to country/study-specific situations. A total of 16 facets with varying numbers of descriptors was selected by experienced dieticians to be included in the GloboDiet accustomed for DNFCS 2007–2010, based on the Dutch food market and the purposes of the data collected (Table 1).

**Table 1:** The list of facets and the examples of the corresponding descriptors in Globodiet for DNFCS 2007–2010

	Facet Names	Number of Descriptors	Examples of Descriptors
1	Source	21	beef, goat, pork …
2	Physical state/form as quantified	28	liquid, reconstituted from powder, minced …
3	Cooking method	28	cooked, baked, barbecued …
4	Preservation method	13	canned, frozen, dried …
5	Packing medium	22	canned in oil, canned in water …
6	Flavoured component	37	nuts, spices, mint …
7	Sugar content	6	non sweetened, sweetened, sugar reduced …
8	Fat content	39	whole, partially skimmed, skimmed …
9	Type of packing	4	in box, in paper, in bottle …
10	Food production	12	homemade fat used known, commercial fat used unknown …
11	Enriched/fortified	11	vitamins, mineral components, dietary fibre...
12	Brand name (yes/no)^a^	2	yes, no
13	Skin consumed	3	undefined, without skin, with skin
14	Visible fat consumed	3	undefined, without visible fat, with visible fat
15	Type of fat used	2	no fat used, choose from the food list
16	Type of milk/liquid used	13	milk, whole milk, skimmed milk...

^a^A brand name would be entered if participants chose the descriptor ‘yes’, entered brand names were not put in the random forest analysis in this study

### Data handling

The total collected consumption data from all participants for the two 24HRs has 219,006 food records, with 350,369 descriptors ranging from 0 to a maximum of 8 for each record. A number of 26,679 unique combinations of foods with descriptors was reached. Trained dieticians linked all combinations to 1599 most appropriate food codes in the Dutch National Food Composition Database (NEVO Table 2011/3.0), which contains energy, macro- and micronutrient contents of 2389 food codes in total [25].

### Statistical analysis

To assess the importance of the GloboDiet facets in predicting the nutrient contents of consumed foods in DNFCS, we used random forest [26]. Random forest is a prediction model that consists of a multitude of decision trees. Each tree is trained on different subsets of training data, and the remaining data (not used for the training) are used to estimate prediction error and variable importance. In our study, foods consumed by all participants in both 24HRs were used for predicting facet importance, the number of randomly selected variables to be considered when splitting the tree at each node was set to its default value (mtry = Total number of predictor variables/3); the number of trees for each nutrient was set at 10,000. Stratified by food group, the importance of a facet (denoted by %IncMSE), was calculated as the percentage increase in prediction error, when data for that facet were permuted in the dataset while keeping data for the other facets unchanged. The random forest algorithm was applied through the randomForest package in Rstudio 1.1.383.

The 24HR variables of 16 facets, food IDs (a series of numbers identifying food items) and food subgroups (elements of main food groups) were regarded as predictor variables. The detailed food group information can be found in Additional file 1. The energy and 33 macro- and micronutrients were regarded as response variables and were predicted one by one with the prediction variables. Food IDs were treated as continuous variables because it exceeds the limit of 32 levels allowed to categorical variables in the implementation of random forest. As comparable foods were numbered sequentially, treating food ID as continuous is reasonable. Facets were treated as categorical variables. Facet “Flavoured/added components” was separated into three sub-groups based on the category (nuts, sugary, savoury) of its descriptors, since the number of descriptors also exceeded the allowed 32 for categorical variables like in food IDs. The variable brand name was not included as a predictor, as this consists of a free text field, yielding many unordered categories that were difficult to separate into sub-groups. Instead, we included the facet “Brand name (yes/no)” that indicated whether this brand name field was filled in or not.

To facilitate the comparison of the relative importance of facets between nutrients, within each food group and each nutrient, %IncMSEs were normalized by dividing them by the highest %IncMSE over the facets. The maximum normalized %IncMSE for the facet across all nutrients would be retained for each food group. After deleting facets with a maximum normalized %IncMSE lower than 0.80 in each food group, small effects on population nutrient intake distributions were observed, therefore a cut-off point at 1.00 was chosen for more significant results. Hence, in each food group, facets with a normalized value below 1.00 for all nutrients were considered unimportant.

### Simulation study

We conducted a simulation study to investigate if deleting unimportant facets could affect the population nutrient intake. We summarized the average nutrient intake of two 24 HRs for each participant and calculated the population nutrient intake distributions in both the original and simulation scenarios.

**Fig. 1:**
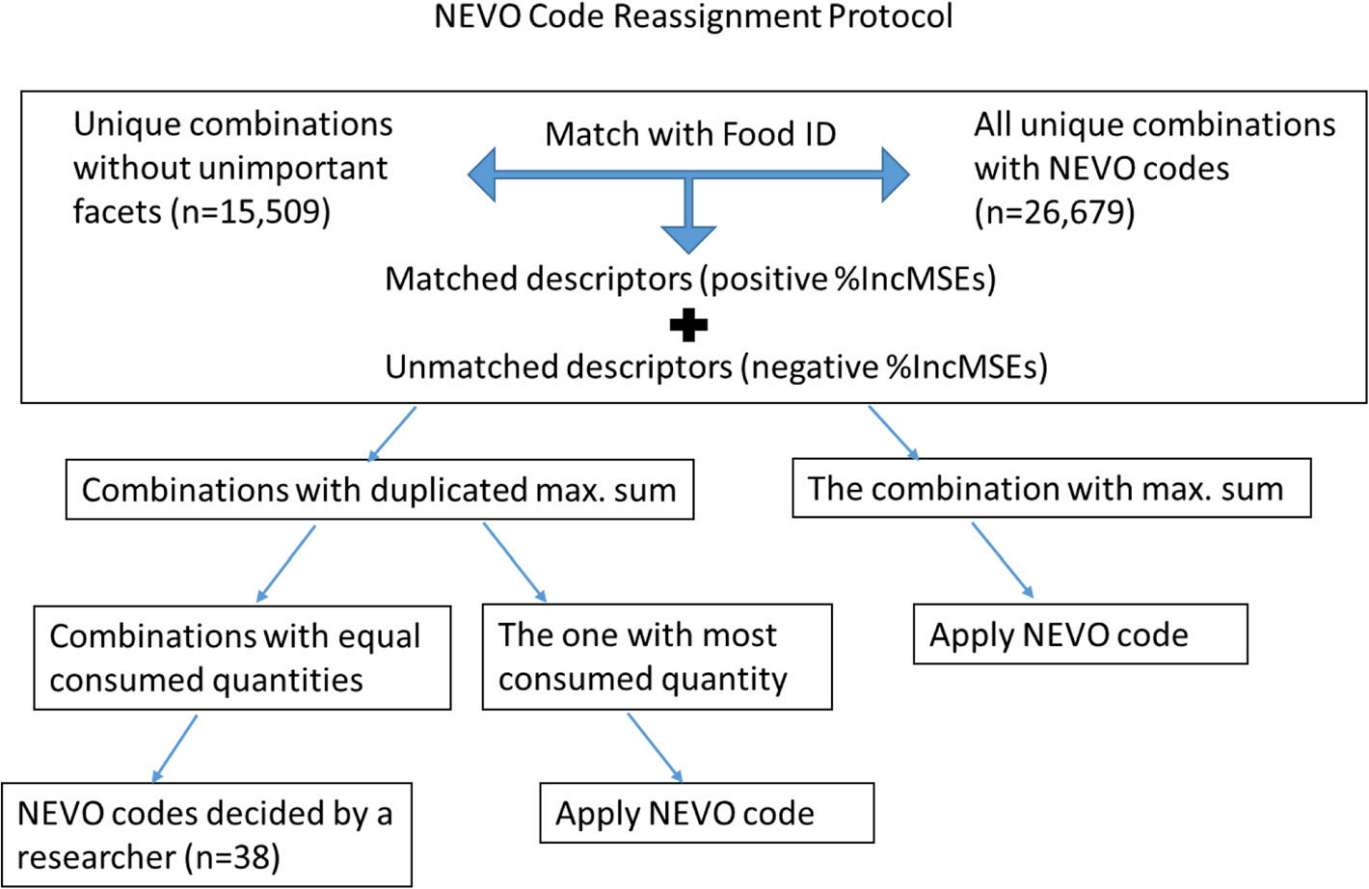
Flow chart of the NEVO code Reassignment Protocol. A NEVO code was assigned to each relinking combination according to the NEVO codes of the same food with the most similar descriptor combinations that have been linked by dieticians during the survey period. The combinations received a positive score for each identical pair of descriptors (equal to the maximum normalized %IncMSEs) and a penalty for descriptors that were different (equal to the negative maximum normalized %IncMSEs). The scores were summed, and the NEVO code of the food-descriptor combination with the highest score was assigned to the combination that needed to be relinked. In case there were more than one NEVO codes with the same highest score, or when no descriptors were left for a food item, the NEVO code of a food-descriptor combination with a higher consumed quantity would be selected. In case the consumed quantities were also the same (occurred in 38 cases), a researcher decided on NEVO code selection

The first step was to create the simulation datasets. After deleting one or more unimportant facets, we linked new unique food-descriptor combinations to the national food composition database NEVO semi-automatically. As illustrated in Fig. 1, for each new combination, a NEVO code was assigned based on the NEVO codes that have been linked to the same food with the most similar descriptor combinations by dieticians during the survey period. To identify the most similar descriptor combination, we gave combinations a positive score for each identical pair of descriptors (equal to the maximum normalized %IncMSEs) and a penalty for descriptors that were different (equal to the negative maximum normalized %IncMSEs). The scores were summed, and the NEVO code of the food-descriptor combination with the highest score was assigned to the combination that needed to be relinked. In case there were more than one NEVO codes with the same highest score, or when no descriptors were left for a food item after deleting unimportant facets, the NEVO code of a food-descriptor combination with a higher consumed quantity would be selected. In case the consumed quantities were also the same (occurred in 38 cases), a researcher decided on NEVO code selection.

To summarize the population nutrient intake distributions in both the original dataset and the simulation dataset, the energy and nutrient contents for 100 g of foods in NEVO were multiplied with the quantities consumed in DNFCS 2007–2010, averaged over two days of each participant. All results were weighted for small deviances in sociodemographic characteristics (age, sex, region, the degree of urbanisation and educational level), the day of the week and the season of data collection, to give results that are representative for the Dutch population and representative for all days of the week and all seasons. The mean, median, 5th, 25th, 75th, 95th percentile and the percent differences of consumption per nutrient between the original and simulation dataset were calculated for the total population and stratified by gender and age group (7–18 years old and 19–69 years old). The population nutrient intake distributions were conducted using the SAS 9.4, and the percent difference between the original and simulation dataset was calculated using Excel 2016 software.

## Results

Table 2 shows the normalized maximum importance (%IncMSEs) of 16 facets in predicting the nutrient contents of food items within each of 17 food groups. Using a cut-off point of 1.00, we identified a total of 64 out of 112 facets across food groups as unimportant, whereas a total of 50 facets fell below the cut-off point at 0.80. For a cut-off point at 0.80, 22% of the 350,369 facet descriptors were deleted in the total food consumption database. The majority of the percent difference between percentile estimates of the population nutrient intake distributions before and after facet deletion ranged from 0 to 1%, while only 2% cases ranged from 1 to 5% (Additional file 2).

**Table 2:** The maximum normalized %IncMSEs of the existing facets in each food group

Food groups																			
		1	2	3	4	5	6	7	8	9	10	11	12	13	14	15	16	17	
	Facet names	Potatoes	Vegetables	Legumes	Fruits, nuts, olives	Dairy (products)	Cereal (products)	Meat (products)	Fish and shellfish	Egg (products)	Fats and oils	Sugar and confectionery	Cakes and sweet biscuits	Non-alcoholic beverages	Alcoholic beverages	Condiments and sauces	Soups	Miscellaneous	# of Omitted/# of original
1	Source					0.53*		0.00*		0.12*									3/3
2	Physical state/form as quantified	1.00	1.00	0.34*	0.73*	1.00			0.80*					1.00		1.00	1.00	1.00	3/10
3	Cooking method	0.77*	1.00	0.31*	1.00	0.26*	1.00	1.00	1.00	1.00								1.00	3/10
4	Preservation method	1.00	1.00	0.61*	1.00	0.35*	0.51*	0.70*	1.00									0.52*	5/9
5	Packing medium	0.02*	0.87*	1.00	0.61*	0.00*	0.40*	0.00*	1.00									0.00*	7/9
6A^a^	Flavoured component A											1.00		0.50*				0.74*	2/3
6B	Flavoured component B					1.00	0.96*							0.50*				0.63*	3/4
6C	Flavoured component C											1.00	0.83*	0.67*				0.65*	3/4
7	Sugar content				0.57*	1.00	1.00					1.00		1.00				0.60*	2/6
8	Fat content				1.00	1.00	1.00	0.00*			1.00							0.60*	2/6
9	Type of packing										1.00								0/1
10	Food production					0.68*	0.68*						0.89*	1.00		1.00	0.92*	0.41*	5/7
11	Enriched/fortified					0.89*	1.00					1.00	0.50*	0.85*				1.00	3/6
12	Brandname (yes/no)	0.69*			0.73*	0.85*	1.00	0.00*	0.00*		1.00	0.96*	0.84*	1.00	1.00	0.87*	0.82*	0.74*	10/14
13	Skin consumed	1.00	1.00		0.82*			0.00*	0.40*										3/5
14	Visible fat consumed							0.00*											1/1
15	Type of fat used	1.00		0.31*			0.06*						0.45*			0.90*	0.38*		5/6
16	Type of milk/liquid used	1.00		0.30*		0.63*	1.00						0.60*			1.00	1.00	0.70*	4/8
	# of omitted/ # of original	3/8	1/5	5/6	5/8	8/12	5/11	7/8	3/6	1/2	0/3	1/5	6/6	4/8	0/1	2/5	3/5	10/13	

*Facets to be omitted for the corresponding food group with the maximum normalized %IncMSEs (among all nutrients) below the cut-off point of 1.00

^a^Random forests require categorical predictors to have no more than 32 levels. Facet 6 (flavoured component) has more than 32 levels and was categorized into three sub-groups (6A, 6B, 6C) based on the category of flavours (nuts, sugary, savoury)

From Table 2, for a cut-off point at 1.00, no facets were unimportant in the food groups ‘Fats and oils’ and ‘Alcoholic beverages’, whereas all facets were unimportant for ‘Cakes and sweet biscuits’. The food group ‘Miscellaneous’ has the largest amount of unimportant facets than the rest of the food groups. In the ‘Meat’ group, most facets had zero effect in predicting food groups, including ‘Source’, ‘Packing medium’, ‘Fat content’, ‘Brand name (yes/no)’, ‘skin consumed, and ‘visible fat consumed’.

From the facet perspective, ‘Brand name (yes/no)’ and ‘Packing medium’ were unimportant for the most of the food groups (10 and 7 food groups, respectively). The number of deletions ranged from 1 to 5 times for the rest of the facets. ‘Source’ and ‘Visible fat consumed’ were unimportant for all the food groups for which they are relevant (3 and 1 food groups, respectively). On the other hand, ‘Physical state’ and ‘Cooking method’ were strong predictors (importance of 1.00) for the largest number of food groups. Facet ‘Type of packing’ was only available for the food group ‘Fats and oils’ and was a strong predictor for that food group. Despite ‘Brand name (yes/no)’ was unimportant for most of the food groups, it was a strong predictor for food group ‘Cereals’, ‘Fats and oils’, ‘Alcoholic’ and ‘Non-alcoholic beverages’. Full results of the facet importance for each nutrient in each food group can be found in Additional file 3.

In the original total food consumption database, 35% (121,015 out of 350,369) of the total descriptors used were identified as unimportant, which has resulted a NEVO code change of 11% (2923 out of 26,679) combinations in the unique food dataset and 3.7% (8196 out of 219,006) combinations in the total food consumption dataset.

After reassigning the NEVO codes, the population means and percentiles of two days’ average energy and nutrient intakes in DNFCS 2007–2010 were calculated, as well as the percent difference between the original and the simulation result. Table 3 shows the results of energy and ten nutrients that were commonly found in the nutrition facts label. The results of all nutrients can be found in Additional file 4. The majority (79.4%) of the percent difference between distribution percentiles before and after facet deletion ranged from 0 to 1%, while 20% cases ranged from 1 to 5% and 0.6% cases more than 10%. Percent difference larger than 1% were mainly found in vitamins. Differences more than 10% appeared mostly in vitamins for 7–18-year-olds and in the extreme percentiles P5 and P95. Some of the differences that were larger than 10% were small as the absolute difference. For example, the most significant difference of 14.1% was for the P95 of vitamin B6; but the absolute difference of the two scenarios was 0.5 mg (rounded to mg). No general patterns were found on nutrient over- and underestimation after facet deletion for most nutrients. However, lower vitamin C contents were found in each percentile after facet deletion for all age groups, whereas higher amounts of vitamin B group were found after facet deletion.

**Table 3:** The population means and percentiles of two days’ average energy and ten nutrients’ intake distributions before and after facets’ deletion at cut-off at 1.00

Nutrients	7–18 years						19–69 years						All ages					
	Male			Female			Male			Female			Male		Female			
	Before	After	% D^a^	Before	After	% D	Before	After	% D	Before	After	% D	Before	After	% D	Before	After	% D
Energy (kcal)																		
P5	1457	1456	0.0	1314	1318	−0.3	1482	1481	0.0	1121	1134	−1.1	1481	1475	0.4	1163	1165	−0.2
P25	1859	1857	0.1	1671	1669	0.1	2048	2042	0.3	1539	1539	0.0	2010	2009	0.1	1565	1567	−0.1
P50	2226	2226	0.0	1915	1920	−0.2	2483	2487	−0.2	1837	1834	0.2	2421	2427	−0.2	1853	1851	0.1
P75	2653	2657	−0.1	2200	2199	0.0	2964	2965	0.0	2223	2225	−0.1	2907	2906	0.0	2220	2225	−0.2
P95	3676	3654	0.6	2694	2694	0.0	3749	3764	−0.4	2834	2830	0.2	3742	3745	−0.1	2813	2806	0.2
Mean	2326	2328	−0.1	1951	1953	−0.1	2534	2539	−0.2	1902	1905	−0.1	2496	2500	− 0.2	1911	1914	−0.1
Protein (g)																		
P5	41	41	−0.4	37	37	0.0	55	55	0.0	42	42	0.0	49	49	0.0	41	41	−0.2
P25	56	56	−0.3	50	50	−0.1	74	74	−0.1	57	57	−0.1	70	70	−0.4	55	55	−0.3
P50	69	70	−0.8	59	59	0.2	87	88	−0.2	68	68	0.0	84	84	0.3	66	66	−0.1
P75	86	86	0.2	70	70	−0.2	105	105	0.4	82	82	0.1	102	102	−0.1	80	80	0.0
P95	114	114	0.0	91	91	0.4	133	132	0.5	102	102	0.0	131	130	0.3	101	101	0.2
Mean	72	72	0.0	61	61	0.0	90	90	0.0	70	70	0.1	87	87	0.0	69	68	0.1
Fat (g)																		
P5	45	45	0.1	40	41	−0.9	47	47	−0.1	34	34	0.2	47	47	−0.4	35	35	−0.8
P25	63	63	0.1	56	56	0.1	73	73	−0.3	52	53	−0.4	71	71	0.4	53	53	−0.4
P50	83	83	−0.9	70	70	−0.8	95	95	−0.3	68	69	−0.1	92	93	−0.3	69	69	−0.1
P75	102	103	−0.9	86	87	−1.2	118	119	−1.0	90	90	−0.5	116	117	−1.3	89	89	−0.4
P95	151	151	0.0	112	112	0.0	158	158	−0.3	122	123	−0.7	158	158	−0.1	120	121	−0.9
Mean	87	87	−0.3	72	73	−0.4	98	98	−0.4	73	73	−0.4	96	96	−0.4	73	73	−0.4
Saturated fatty acids (g)																		
P5	16	16	−0.9	14	14	0.2	16	16	−0.1	12	12	−0.1	16	16	−0.1	12	13	−4.1
P25	23	23	0.6	21	21	0.5	27	27	−0.4	20	20	−1.7	26	26	−0.3	20	20	−1.8
P50	30	30	−0.7	27	27	−1.3	34	35	−0.3	26	27	−1.4	33	34	−0.5	26	27	−1.1
P75	39	39	−0.4	33	33	−0.3	44	44	−0.5	35	35	−0.1	43	43	−0.5	34	34	−0.5
P95	54	54	−0.7	44	44	−0.2	60	61	−0.6	49	49	0.6	58	59	−0.8	48	48	−0.1
Mean	32	32	−0.3	27	28	−0.4	36	36	−0.7	28	28	−0.7	35	35	−0.6	28	28	−0.6
Carbohydrates (g)																		
P5	187	188	−0.1	160	161	−0.3	147	147	0.0	117	117	0.4	151	150	0.1	121	121	−0.1
P25	238	237	0.4	213	212	0.5	214	213	0.4	171	170	0.1	218	219	−0.1	176	177	− 0.5
P50	287	288	0.0	246	246	0.0	266	265	0.3	208	209	−0.3	270	270	−0.2	216	216	0.0
P75	344	344	0.0	286	286	0.2	330	330	0.0	251	251	0.1	334	334	−0.1	260	260	0.1
P95	454	454	0.0	359	361	−0.4	436	436	0.1	338	337	0.2	439	438	0.2	341	340	0.2
Mean	299	299	0.0	252	251	0.1	277	277	−0.1	215	215	0.0	281	281	−0.1	222	222	0.0
Monosaccharides (g)																		
P5	79	79	0.9	65	65	−0.2	42	43	− 0.4	38	38	−1.1	46	45	0.4	41	41	0.3
P25	120	120	0.1	103	102	0.8	82	81	0.8	73	72	0.4	86	86	0.0	76	76	0.3
P50	148	148	0.3	131	130	0.2	114	114	−0.1	96	96	0.0	122	122	0.3	103	102	0.1
P75	185	185	0.3	161	160	1.0	159	159	0.2	129	129	0.2	166	165	0.3	137	137	0.0
P95	263	263	0.0	218	218	−0.1	243	243	0.2	193	194	−1.0	246	246	0.0	200	201	−0.6
Mean	156	156	0.2	134	133	0.4	125	125	0.1	104	104	−0.1	131	130	0.1	109	109	0.0
Fibre (g)																		
P5	9.8	9.79	0.0	9.0	9.1	−0.3	11.6	11.6	0.1	9.6	9.6	0.0	10.9	10.9	0.0	9.5	9.5	0.0
P25	13.9	14.2	−2.2	12.6	12.7	−0.8	16.9	16.9	−0.1	14.2	14.1	0.5	16.3	16.3	−0.3	13.9	13.9	−0.2
P50	17.5	17.8	−1.9	15.5	15.4	0.6	21.5	21.6	−0.2	17.6	17.5	0.1	21.0	21.0	−0.1	17.2	17.2	0.0
P75	21.7	21.8	−0.4	18.6	18.8	−0.7	26.4	26.6	−0.8	22.0	22.0	0.1	25.8	26.0	−0.7	21.3	21.3	−0.1
P95	29.9	29.9	0.0	24.6	25.0	−1.5	35.6	35.3	0.8	28.6	28.6	−0.1	35.3	35.1	0.7	28.4	28.3	0.3
Mean	18.5	18.5	−0.4	16.0	16.1	−0.5	22.3	22.3	0.0	18.4	18.4	0.2	21.6	21.6	0.0	18.0	18.0	0.0
Sodium (mg)																		
P5	1447	1444	0.2	1273	1270	0.2	1625	1622	0.2	1226	1219	0.6	1533	1559	−1.7	1234	1228	0.5
P25	1951	1953	−0.1	1748	1754	−0.3	2357	2364	−0.3	1773	1789	−0.9	2270	2272	−0.1	1767	1785	−1.0
P50	2473	2492	−0.8	2139	2134	0.2	2970	2986	−0.5	2236	2239	−0.1	2873	2890	−0.6	2225	2224	0.1
P75	3147	3152	−0.2	2567	2568	0.0	3630	3644	−0.4	2812	2825	−0.5	3540	3561	−0.6	2782	2782	0.0
P95	4256	4260	−0.1	3398	3409	−0.3	4792	4830	−0.8	3772	3780	−0.2	4739	4745	−0.1	3668	3674	−0.2
Mean	2611	2619	−0.3	2195	2198	−0.1	3058	3065	−0.2	2336	2346	−0.4	2977	2984	−0.2	2311	2320	−0.4
Calcium (mg)																		
P5	391	391	−0.1	332	332	0.0	470	469	0.2	429	429	0.0	448	449	−0.2	404	404	0.0
P25	686	690	−0.5	616	627	−1.9	791	792	−0.1	708	710	−0.3	769	769	0.0	695	696	−0.2
P50	922	917	0.5	828	834	−0.7	1092	1091	0.1	944	941	0.3	1049	1055	−0.6	924	924	0.0
P75	1232	1238	−0.5	1072	1080	−0.7	1448	1441	0.4	1195	1199	−0.3	1403	1401	0.1	1176	1179	−0.2
P95	1832	1865	−1.8	1545	1545	0.0	1999	1999	0.0	1702	1702	0.0	1998	1998	0.0	1677	1681	−0.2
Mean	995	1000	−0.4	874	880	−0.6	1148	1150	−0.1	992	992	0.0	1121	1122	−0.1	971	973	−0.1
Vitamin C (mg)																		
P5	24	24	1.3	23	21	**8.3**	26	27	−1.2	24	24	0.3	26	26	0.0	24	23	2.0
P25	48	46	3.6	47	44	**5.2**	50	50	−0.1	51	51	0.0	50	49	1.2	50	48	2.8
P50	76	72	**5.4**	73	67	**8.4**	84	83	1.1	83	81	2.1	82	80	1.7	81	79	2.2
P75	115	109	4.9	110	106	4.1	129	127	2.0	126	124	1.6	127	123	3.3	122	119	2.3
P95	176	167	**5.2**	169	165	2.6	218	218	0.0	209	206	1.3	210	208	1.0	204	201	1.9
Mean	86	82	4.8	84	79	**5.3**	98	96	1.5	95	94	1.6	96	94	2.1	93	91	2.2
Vitamin B6 (mg)																		
P5	0.7	0.7	−4.6	0.7	0.7	−3.0	0.9	0.9	−0.2	0.7	0.7	0.2	0.9	0.9	−1.5	0.7	0.7	−2.1
P25	1.1	1.2	−3.7	1.0	1.0	−4.1	1.5	1.5	−1.9	1.1	1.1	−1.0	1.4	1.4	−2.2	1.1	1.1	−2.0
P50	1.6	1.6	−4.2	1.3	1.4	**−6.2**	2.0	2.0	−1.5	1.5	1.6	−1.2	1.9	1.9	−2.6	1.5	1.5	−2.8
P75	2.2	2.4	**−10.4**	1.8	1.9	**−5.1**	2.5	2.6	−3.0	2.1	2.1	−1.0	2.5	2.6	−4.4	2.0	2.1	−2.2
P95	3.8	4.3	**−14.1**	3.0	3.4	**−13.5**	4.1	4.3	−2.6	3.4	3.5	−3.1	4.1	4.3	−4.1	3.3	3.5	**−6.8**
Mean	1.8	1.9	**−7.5**	1.5	1.6	**−7.6**	2.2	2.2	−2.5	1.7	1.8	−1.9	2.1	2.2	−3.3	1.7	1.7	−2.8

^a^% D represents the percent difference of nutrient intake distributions before and after facets’ deletion for the Dutch population aged 7 to 69 years

Percent difference larger than 5% are shown in bold

## Discussion

To enhance the efficiency of data collection and handling of GloboDiet 24HRs, we explored the option of deleting less important food characteristics (facets) from the interview. The importance of each facet in predicting nutrient contents in foods was determined by the random forest algorithm. When the 35% least predictive facets were deleted from the dataset of the Dutch National Food Consumption Survey 2007–2010, the difference between the original and simulated population nutrient intake distributions was small for the majority of the nutrients.

There are several possible explanations for certain facets to be less or more predictive in certain food groups. One reason for less predictive facets is that some facets were only applicable to a few food items in certain food groups, and those food items were rarely consumed. An example of this is the facet ‘Enriched/fortified’ in the food group ‘Cakes and sweet biscuits’. A second reason is a lack of variation in the chosen descriptors within a facet. An example of this is the facet ‘source’ in dairy products since cow milk is the basis for the majority of the consumed dairy products in the Netherlands. Another possible explanation for the less predictive facets is the use of a generic food composition database NEVO [27]. Some facets might have been important for predicting true nutrient levels but not for averaged nutrient levels of generic foods. For example, the facet ‘Brand name (yes/no)’, which could typically be a good predictor for nutrient levels in industrially processed foods [28], showed low predicting power for most of the food groups in this study.

In contrast, some facets showed strong predictive power in estimating nutrient contents in certain food groups. The facet ‘Type of packing’ predicted strongly for the ‘Fats and oils’ group, because the type of packing materials could distinguish solid from liquid fat. Hence, the variance in the fat content between solid and liquid fat could be differentiated by the facet ‘Type of packing’. Similarly, as can be expected from a nutrition point of view, facet ‘Physical state’, ‘Sugar’ and ‘Fat content’ were strong predictors for most of their allocated food groups, except for unprocessed products (e.g., fruit, meat, and fish).

In terms of comparing nutrient intake distributions before and after the facets had been deleted, a difference of less than 10% was found for most nutrients. A similar finding was observed in a study that investigated the effect of a concise versus an extensive food list in a self-administered web-based 24HR tool. They found that the differences between population nutrient intakes assessed by two methods were less than 6% [29], which is consistent with our study that the majority of the differences fell below 5% before and after facet deletion. In this study, the small difference could be explained by the fact that 96.3% of the combinations were relinked to the same food code in the food composition database. From this, we speculated that sufficient information for NEVO linkage could be provided by the food names and remaining facets. For those combinations with deleted facets that were linked to different food codes in the food composition database, the difference in nutrient contents of the original and alternative food codes may have been small, or the foods were consumed by few persons or in small amounts and therefore did not influence population nutrient intake distributions substantially.

Specifically, a significant decrease in the amount of vitamin C was found for children in our study, and the reason was speculated to be the deletion of the facet ‘Enriched/fortified’ in the food group ‘Non-alcoholic beverages’. According to the report of 2007–2010 survey, ‘Non-alcoholic beverages’ and ‘Meat and meat products’ together, contribute for one third to the total vitamin C intake partly due to food fortification and processing [24]. Hence, beverages with fortification were linked to NEVO codes for products without fortification and resulted in a lower vitamin C content. On the other hand, a large increase in the amount of vitamin B group was found for children. A possible explanation would be the deletion of ‘Flavoured component’ in the food group of ‘Cereal’, which may have caused a linkage between flavoured cereals to cereals without flavours (i.e., whole wheat cereals) which normally have higher vitamin B contents. A closer investigation should be conducted before deleting facets in the real setting.

To our knowledge, this is the first study investigating the impact of reducing food descriptions in interview-based 24HRs for the estimation of population nutrient intake distributions. Until now decisions on the facets that were included in the 24HR interview of DNFCS were based on expert judgment. A strength of our approach is that both evaluating the facet importance and assessing their impact was data-driven. Another strength is the use of the random forest for the identification of unimportant facets. This prediction model is more efficient in large datasets, has a lower risk of overfitting and is better in dealing with correlated predictors than multiple linear regression [30]. However, the applied random forest implementation only allows nominal variables with a limited number of levels as predictors. Therefore, the nominal variable “food ID” was treated as a continuous variable, and the importance of the information on the full brand and product name of each food could not be evaluated. Also, the importance of the facet “Cooking method” could not fully be assessed, since the added fat in case of frying was not included in the nutrient content of the food, but became a separate food item in the food consumption database. Another limitation of our study was the use of a semi-automated protocol of reassigning a different NEVO code to combinations with deleted facets rather than applying the original approach of ‘manual’ linkage by dieticians. However, manual matching would only have further decreased the effect of facet deletion, so we do not think our conclusions would have been different. Finally, the impact of facet reduction on respondents’ answers during the food description part of the interview was not assessed. Although a face-to-face or telephone 24HR interview has generally smaller self-reporting error than other methods, measurement error still exists (i.e., rely on memory, underreporting) [6]. However, we assume that the effect of facet reduction on self-reporting error will be small.

The scope of our analyses focused on the nutrition aspects in deleting facets, while other aspects can be important as well. One example is the facet ‘Physical state’, which is essential in quantifying the consumed foods, e.g., coffee powder is quantified differently than coffee as a beverage. Moreover, deleting facets that could estimate exposure to potentially harmful substances should be carefully considered for practical use. For example, facets related to food preparation should be kept for some foods like meats since it is a crucial food characteristic to identify microbiological risks. In principle, the procedure described in this manuscript can also be applied to evaluate facet importance for food chemical distributions. The facets and descriptors of the GloboDiet software can be tailored for any new study [17]. Researchers use this software should thus consider carefully which food characteristics are important for their study aims before the start of a study.

The objective of looking at the reduction in food characteristics was to enhance efficiency in conducting future surveys. Less extensive food description would result in a shorter interview duration and less work in linking the food with the food composition database. The time needed to go through facets for all consumed foods was estimated to be 15 min out of a 44 min 24HR interview. Without 35% of the unimportant facets, the time saved for one interview would on average be 5 min. In a survey with 3819 participants that are interviewed twice, a total of 637 h would be saved. Moreover, less extensive food description during data collection would lead to fewer unique food-descriptor combinations reported in a survey. In the data handling phase, each unique food-descriptor combination needs to be linked manually to the food composition database, which would cost 5 to 10 min approximately. Hence, a reduced number of 3534 unique combinations (from 26,679 to 23,145) after deleting less important facets would save around 442 h. To sum up, we estimated that around 1079 h would be saved for both data collection and handling if facet deletion would be applied.

The current study focused on reducing the number of facets as a potential efficiency measure for a national food consumption survey. Other alternative efficiency options have also been studied elsewhere. One alternative is to use 24-h dietary recall software to guide the interviews in which the food list is directly related to the foods in the national food composition database [9, 31]. The reason why GloboDiet did not choose this option was to give flexibility for new foods that have entered the food market (but have not been included in food composition databases yet), to standardize food description across different countries that use the same software, and to be able to collect characteristics of food relevant for other purposes than nutrient intake estimations [17]. A more cost-efficient alternative regarding dietary assessment is to use self-administered methods. However, the accuracy and reliability of those tools need to be further evaluated, due to self-reporting errors, and various levels of acceptance by different age-groups [32]. Furthermore, matching food consumption and food composition data could be more efficient through automatic or semi-automatic linkages. In this study, decisions on NEVO code reassignment for food-descriptor combinations were made based on a simple algorithm with the results of the random forest algorithm. For matching future food consumption data automatically or semi-automatically, random forest prediction models using available previously matched food consumption and food composition data as training dataset could be developed. Similar approaches have been developed in some studies including a semi-automatic food matching technique using machine-learning and a natural language processing approach. These approaches have shown a promising future of replacing manual linkage between food and food composition database [33, 34].

## Conclusion

In conclusion, the data-driven procedure that combined random forest prediction with a simulation study was successful in identifying less important characteristics of food description. After deleting those less important characteristics, there was little impact on the population nutrient intake distributions for most nutrients, thus yielding a promising approach for saving labour and costs.

## Additional files

Additional file 1: Dutch National Food Consumption Survey 2007–2010 NEVO-codes and EPIC-Soft group classification. (DOCX 316 kb)

Additional file 2: The population two days’ average of energy and nutrient intake distributions before and after facets’ deletion at cut-off at 0.8. The population means and percentiles of two days’ average energy, macro- and micronutrient intake distributions before and after facets’ deletion and percent difference (% D) for the Dutch population aged 7 to 69 years (DNFCS 2007–2010) (*n* = 3819). (XLSX 62 kb)

Additional file 3: The normalized %IncMSEs of the existing facets in each food group calculated by random forests. The normalized percent increase in mean square error (%IncMSEs) (among energy and thirty-three nutrients) of the existing facets in each food group calculated by random forests using the data from DNFCS 2007–2010. (XLSX 65 kb)

Additional file 4: The population two days’ average of energy and nutrient intake distributions before and after facets’ deletion at cut-off at 1.00. The population means and percentiles of two days’ average of energy, macro- and micronutrient intake distributions before and after facets’ deletion and percent difference (% D) for the Dutch population aged 7 to 69 years (DNFCS 2007–2010) (n = 3819). (XLSX 72 kb)

## Abbreviations

%IncMSE: Percentage increase in prediction error
24HRs: 24-h dietary recalls
AMPM: Automated Multiple-Pass Method
DNFCS: Dutch National Food Consumption Surveys
IARC: International Agency for Research on Cancer
NEVO: Dutch National Food Composition Database
USDA: U.S. Department of Agriculture
