# Supplementary material for: Importance of details in food descriptions in estimating population nutrient intake distributions
Source: Nutr J. 2019 Mar 15;18:17. doi: 10.1186/s12937-019-0443-5 (PMC6419831; doi:10.1186/s12937-019-0443-5)
Supplement: Supplementary file 1 — Dutch National Food Consumption Survey 2007–2010 NEVO-codes and EPIC-Soft group classification. (DOCX 316 kb) [file 12937_2019_443_MOESM1_ESM.docx]

|  |
| --- |
| Additional File 1  Dutch National Food Consumption Survey 2007-2010    **Description of the database**  NEVO-codes and EPIC-Soft group classification  Zohreh Etemad  Robert Jan de Klein  Jan D. van der Laan  Marga C. Ocké  Caroline T.M. van Rossum  Contact:  Zohreh Etemad  Centre for Nutrition, Prevention and Health Services (VPZ)  [zohreh.etemad@rivm.nl](mailto:zohreh.etemad@rivm.nl) |
| Version 1.0 (November 2011)  © RIVM 2011 |
|  |

NEVO-codes and EPIC-Soft group classification

| **EPIC-Soft group** | | | **NEVO code** | **English** | **Dutch** |
| --- | --- | --- | --- | --- | --- |
| **1** |  |  |  | **POTATOES AND OTHER TUBERS** |  |
| **1** | **1** |  |  | **POTATOES** |  |
|  |  |  | 121 | Potatoes mashed prepared with semi-skimmed milk and marg | Aardappelpuree bereid m hv melk m marg |
|  |  |  | 948 | Rösti prepared without fat | Rösti bereid z vet |
|  |  |  | 982 | Potatoes without skin boiled average | Aardappelen z schil gekookt gem |
|  |  |  | 1150 | Potatoes sliced frozen unprepared | Aardappelschijfjes diepvries onbereid |
|  |  |  | 1455 | Potatoes mashed instant powder average | Aardappelpureepoeder gem |
|  |  |  | 1456 | Chips pre-fried unprepared | Frites voorgebakken onbereid |
|  |  |  | 1679 | Chips oven frozen prepared | Frites oven- diepvries bereid |
|  |  |  | 2108 | Chips prepared average | Frites bereid gem |
|  |  |  | 2322 | Potatoes mashed instant semi-skimmed milk | Aardappelpuree instant bereid m halfvolle melk |
|  |  |  | 2323 | Potatoes mashed instant prepared with water | Aardappelpuree instant bereid m water |
|  |  |  | 2324 | Potatoes mashed prepared with semi-skimmed milk without fat | Aardappelpuree bereid m hv melk z vet |
|  |  |  | 2325 | Potatoes boiled with skin average | Aardappelen m schil gekookt gem |
|  |  |  | 2326 | Potato croquettes frozen unprepared | Aardappelkroketten diepvries onbereid |
|  |  |  | 2426 | Mashed potatoes prepared with water without fat | Aardappelpuree bereid m water z vet |
|  |  |  | 2574 | Potatoes mashed with semi-skimmed milk and butter | Aardappelpuree bereid m hv melk en boter |
|  |  |  | 2575 | Potatoes mashed with whole milk and margarine | Aardappelpuree bereid m volle melk en margarine |
|  |  |  | 2576 | Potatoes mashed with semi-skimmed milk and halvarine | Aardappelpuree bereid m hv melk en halvarine |
|  |  |  | 2620 | Potatoes mashed inst with semi-skimmed milk/water/marg 2063 | Aardappelpuree instant ber m hv melk/water/marg 2063 |
|  |  |  | 2621 | Potatoes mashed with skimmed milk and liquid fat 2066 | Aardappelpuree ber m mag melk en braadvet vlb 2066 |
|  |  |  | 2834 | Potato waffles/balls frozen unprepared | Aardappelbolletjes/-wafeltjes ed dv onbereid |
|  |  |  | 2867 | Mashed potatoes with water and margarine | Aardappelpuree bereid m water en margarine |
| **1** | **2** |  |  | **OTHER TUBERS** |  |
|  |  |  | 2109 | Cassava boiled | Cassave gekookt |
|  |  |  | 2111 | Yam boiled | Yam gekookt |
|  |  |  | 2112 | Sweet potato boiled | Bataat zoete gekookt |
|  |  |  |  |  |  |
| **2** |  |  |  | **VEGETABLES** |  |
| **2** | **1** |  |  | **LEAFY VEGETABLES (EXCEPT CABBAGES)** | |
|  |  |  | 7 | Endive raw | Andijvie rauw |
|  |  |  | 8 | Endive boiled | Andijvie gekookt |
|  |  |  | 36 | Purslane boiled | Postelein gekookt |
|  |  |  | 38 | Turnip tops raw | Raapstelen rauw |
|  |  |  | 46 | Lettuce head raw | Sla krop- rauw |
|  |  |  | 47 | Lettuce head boiled | Sla gekookt |
|  |  |  | 48 | Swiss chard leaf boiled | Snijbiet gekookt |
|  |  |  | 51 | Spinach raw | Spinazie rauw |
|  |  |  | 52 | Spinach boiled | Spinazie gekookt |
|  |  |  | 65 | Lettuce lambs raw | Sla veld- rauw |
|  |  |  | 67 | Chicory raw | Witlof rauw |
|  |  |  | 68 | Chicory boiled | Witlof gekookt |
|  |  |  | 126 | Cress garden raw | Sterkers rauw |
|  |  |  | 140 | Spinach tinned | Spinazie blik/glas |
|  |  |  | 146 | Spinach frozen boiled | Spinazie diepvries gekookt |
|  |  |  | 651 | Spinach creamed frozen boiled | Spinazie à la crème diepvries gekookt |
|  |  |  | 684 | Tannia leaves raw | Tajerblad rauw |
|  |  |  | 1136 | Endive frozen unprepared | Andijvie diepvries onbereid |
|  |  |  | 1370 | Grape leaves glass | Druivenblad glas |
|  |  |  | 1399 | Lettuce iceberg raw | Sla ijsberg- rauw |
|  |  |  | 1897 | Seaweed kelp raw | Zeewier kelp rauw |
|  |  |  | 1898 | Seaweed agar agar dried | Zeewier agar agar gedroogd |
|  |  |  | 1904 | Vegetables average boiled | Groenten gekookt gem |
|  |  |  | 2327 | Lettuce mixture | Slamelange krop- en friseesla |
|  |  |  | 2346 | Lettuce green average raw | Sla groene gem rauw |
|  |  |  | 2708 | Lettuce red raw | Sla rode rauw |
|  |  |  | 2736 | Rocket raw | Sla rucola rauw |
| **2** | **2** |  |  | **FRUITING VEGETABLES** |  |
|  |  |  | 11 | Aubergine boiled | Aubergine gekookt |
|  |  |  | 27 | Cucumber without skin raw | Komkommer z schil rauw |
|  |  |  | 28 | Cucumber boiled | Komkommer gekookt |
|  |  |  | 31 | Sweet pepper green raw | Paprika groene rauw |
|  |  |  | 32 | Sweet pepper green boiled | Paprika groene gekookt |
|  |  |  | 131 | Gherkins sour pickled | Augurken zuur |
|  |  |  | 132 | Gherkins sweet pickled | Augurken zoetzuur |
|  |  |  | 138 | Beans runner tinned | Bonen snij- blik/glas |
|  |  |  | 139 | Beans French tinned | Bonen sperzie- blik/glas |
|  |  |  | 141 | Tomato puree concentrated tinned | Puree tomaten- geconcentreerd blik |
|  |  |  | 680 | Okra raw | Okra rauw |
|  |  |  | 689 | Avocado | Avocado |
|  |  |  | 851 | Sweet pepper sweet/sour pickled | Paprika zoetzuur glas |
|  |  |  | 884 | Sweet pepper red raw | Paprika rode rauw |
|  |  |  | 885 | Sweet pepper red boiled | Paprika rode gekookt |
|  |  |  | 922 | Courgette raw | Courgette rauw |
|  |  |  | 951 | Beans French boiled | Bonen sperzie- gekookt |
|  |  |  | 954 | Beans French frozen boiled | Bonen sperzie- diepvries gekookt |
|  |  |  | 961 | Beans runner boiled | Bonen snij- gekookt |
|  |  |  | 964 | Mange-tout boiled | Peultjes gekookt |
|  |  |  | 966 | Courgette boiled | Courgette gekookt |
|  |  |  | 1021 | Artichoke raw | Artisjok rauw |
|  |  |  | 1114 | Plantain raw | Banaan bak- rauw |
|  |  |  | 1143 | Beans runner frozen unprepared | Bonen snij- diepvries onbereid |
|  |  |  | 1161 | Cucumber sliced pickled | Komkommerschijven zoetzuur glas |
|  |  |  | 1372 | Tomato puree Turkish concentrated tinned | Puree tomaten- Turks geconcentreerd blik |
|  |  |  | 1811 | Beans long yard Kouseband boiled | Kousenband gekookt |
|  |  |  | 2113 | Pumpkin boiled | Pompoen gekookt |
|  |  |  | 2293 | Tomatoes tinned | Tomaat in blik |
|  |  |  | 2377 | Tomatoes dried in oil tin/glass | Tomaat gedroogd in olie blik/pot |
|  |  |  | 2378 | Tomato sun-dried | Tomaat zongedroogd |
|  |  |  | 2402 | Capers | Kappertjes |
|  |  |  | 2498 | Tomatoes sieved | Tomaten gezeefd pak |
|  |  |  | 2524 | Chilli pepper raw | Peper Spaanse rauw |
|  |  |  | 2731 | Tomato cherry raw | Tomaat kers- rauw |
|  |  |  | 2732 | Tomato beef raw | Tomaat vlees- rauw |
|  |  |  | 2733 | Tomato beef boiled | Tomaat vlees- gekookt |
|  |  |  | 2734 | Tomato average raw | Tomaat rauw gem |
|  |  |  | 2735 | Tomato average boiled | Tomaat gekookt gem |
|  |  |  | 2739 | Cucumber with skin raw | Komkommer m schil rauw |
|  |  |  | 2740 | Sweet pepper yellowith raw | Paprika gele rauw |
|  |  |  | 2741 | Sweet pepper yellowith boiled | Paprika gele gekookt |
|  |  |  | 2742 | Sweet pepper average raw | Paprika rauw gem |
|  |  |  | 2743 | Sweet pepper average boiled | Paprika gekookt gem |
| **2** | **3** |  |  | **ROOT VEGETABLES** |  |
|  |  |  | 26 | Celeriac boiled | Selderij knol- gekookt |
|  |  |  | 29 | Swede boiled | Koolraap gekookt |
|  |  |  | 30 | Kohlrabi boiled | Koolrabi gekookt |
|  |  |  | 71 | Carrots raw average | Wortelen rauw gem |
|  |  |  | 72 | Carrots boiled average | Wortelen gekookt gem |
|  |  |  | 124 | Radish red/white raw | Radijs rauw |
|  |  |  | 143 | Carrots tinned | Wortelen blik/glas |
|  |  |  | 558 | Celeriac raw | Selderij knol- rauw |
|  |  |  | 958 | Beetroot boiled | Bieten gekookt |
|  |  |  | 960 | Salsify boiled | Schorseneren gekookt |
|  |  |  | 1454 | Beetroot pickled glass | Bieten rode zoetzuur glas |
| **2** | **4** |  |  | **CABBAGES** |  |
|  |  |  | 14 | Cauliflower raw | Kool bloem- rauw |
|  |  |  | 15 | Cauliflower boiled | Kool bloem- gekookt |
|  |  |  | 16 | Kale curly boiled | Kool boeren- gekookt |
|  |  |  | 21 | Cabbage Chinese raw | Kool Chinese rauw |
|  |  |  | 22 | Cabbage Chinese boiled | Kool Chinese gekookt |
|  |  |  | 25 | Cabbage green boiled | Kool groene gekookt |
|  |  |  | 41 | Cabbage red raw | Kool rode rauw |
|  |  |  | 42 | Cabbage red boiled | Kool rode gekookt |
|  |  |  | 43 | Cabbage Savoy raw | Kool savooie- rauw |
|  |  |  | 44 | Cabbage Savoy boiled | Kool savooie- gekookt |
|  |  |  | 53 | Cabbage oxheart raw | Kool spits- rauw |
|  |  |  | 54 | Cabbage oxheart boiled | Kool spits- gekookt |
|  |  |  | 55 | Brussel sprouts boiled | Spruitjes gekookt |
|  |  |  | 69 | Cabbage white raw | Kool witte rauw |
|  |  |  | 70 | Cabbage white cooked | Kool witte gekookt |
|  |  |  | 73 | Cabbage sauerkraut raw | Kool zuur- rauw |
|  |  |  | 74 | Cabbage sauerkraut cooked | Kool zuur- gekookt |
|  |  |  | 145 | Cabbage red glass | Kool rode blik/glas |
|  |  |  | 650 | Cabbage red with apple pieces frozen boiled | Kool rode m appeltjes diepvries gekookt |
|  |  |  | 673 | Mustard leaf raw | Amsoi rauw |
|  |  |  | 920 | Broccoli boiled | Broccoli gekookt |
|  |  |  | 921 | Broccoli raw | Broccoli rauw |
|  |  |  | 952 | Kale curly frozen boiled | Kool boeren- diepvries gekookt |
|  |  |  | 1147 | Brussel sprouts frozen boiled | Spruitjes diepvries gekookt |
|  |  |  | 1398 | Kale curly boiled glass | Kool boeren- glas |
|  |  |  | 1892 | Cabbage pak-choi raw | Paksoi rauw |
|  |  |  | 2710 | Cabbage red with apple pieces glass | Kool rode m appeltjes blik/glas |
|  |  |  | 2840 | Cabbage red with apple pieces | Kool rode m appeltjes huishoudelijk bereid |
| **2** | **5** |  |  | **MUSHROOMS** |  |
|  |  |  | 18 | Mushrooms chanterelle boiled | Cantharellen gekookt |
|  |  |  | 19 | Mushrooms raw | Champignons rauw |
|  |  |  | 20 | Mushrooms boiled | Champignons gekookt |
|  |  |  | 133 | Mushrooms tinned | Champignons blik/glas |
| **2** | **6** |  |  | **GRAIN AND POD VEGETABLES** |  |
|  |  |  | 57 | Sweet corn boiled | Mais suiker- gekookt |
|  |  |  | 135 | Pea garden super fine tinned | Doperwten zeer fijn blik/glas |
|  |  |  | 142 | Beans broad tinned | Bonen tuin- blik/glas |
|  |  |  | 953 | Peas frozen boiled | Doperwten diepvries gekookt |
|  |  |  | 962 | Beans broad boiled | Bonen tuin- gekookt |
|  |  |  | 963 | Peas fresh boiled | Doperwten gekookt |
|  |  |  | 1148 | Beans broad frozen unprepared | Bonen tuin- diepvries onbereid |
| **2** | **7** |  |  | **ONION, GARLIC** |  |
|  |  |  | 37 | Leek boiled | Prei gekookt |
|  |  |  | 63 | Onion raw | Ui rauw |
|  |  |  | 64 | Onion boiled | Ui gekookt |
|  |  |  | 144 | Silver-skin onions sweet pickled glass | Uien zilver- zoetzuur glas |
|  |  |  | 562 | Leek raw | Prei rauw |
|  |  |  | 830 | Garlic fresh | Knoflook vers |
|  |  |  | 1484 | Onions deep-fried sachet | Uitjes gefrituurd zak |
|  |  |  | 2737 | Onion Welsh raw | Ui sla- rauw |
|  |  |  | 2738 | Onion Welsh boiled | Ui sla- gekookt |
| **2** | **8** |  |  | **STALK VEGETABLES, SPROUTS** |  |
|  |  |  | 13 | Celery boiled | Selderij bleek- gekookt |
|  |  |  | 58 | Bean sprouts raw | Taugé rauw |
|  |  |  | 59 | Bean sprouts boiled | Taugé gekookt |
|  |  |  | 130 | Asparagus tinned | Asperges blik/glas |
|  |  |  | 557 | Celery raw | Selderij bleek- rauw |
|  |  |  | 849 | Fennel raw | Venkel rauw |
|  |  |  | 957 | Asparagus boiled | Asperges gekookt |
|  |  |  | 967 | Fennel boiled | Venkel gekookt |
|  |  |  | 1187 | Bamboo shoots tinned | Bamboespruiten blik/glas |
| **2** | **9** |  |  | **MIXED SALAD, MIXED VEGETABLES** |  |
|  |  |  | 75 | Vegetable mixture for soup raw | Groente soep- rauw |
|  |  |  | 127 | Vegetables mixture raw | Rauwkost gemiddeld |
|  |  |  | 132 | Gherkins sweet pickled | Augurken zoetzuur |
|  |  |  | 136 | Peas and carrots tinned | Doperwten m wortelen blik/glas |
|  |  |  | 850 | Vegetables mixed pickled atjar tjampoer | Groente zoetzuur atjar tjampoer glas |
|  |  |  | 1139 | Peas and carrots frozen unprepared | Doperwten m wortelen diepvries onbereid |
|  |  |  | 1141 | Vegetable mixed Mexico frozen unprepared | Groentemix Mexico diepvries onbereid |
|  |  |  | 1144 | Vegetables mixture for soup frozen | Groente soep- diepvries onbereid |
|  |  |  | 1501 | Herb and vegetable mix sachet | Kruidenmix m groente zakje |
|  |  |  | 1904 | Vegetables average boiled | Groenten gekookt gem |
|  |  |  | 1905 | Vegetables average raw | Groenten rauw gem |
|  |  |  | 2330 | Mange-tout and carrots boiled | Peultjes m wortelen gekookt |
|  |  |  | 2331 | Vegetables mixed for Chinese noodles dish boiled | Bamipakket gekookt |
|  |  |  | 2332 | Herb and vegetable mix prepared sachet | Kruidenmix m groente bereid zakje |
|  |  |  |  |  |  |
| **3** |  |  |  | **LEGUMES** |  |
| **3** | **1** |  |  | **LEGUMES** |  |
|  |  |  | 196 | Peas marrowfat legumes tinned | Kapucijners blik/glas |
|  |  |  | 197 | Beans baked in tomato sauce tinned | Bonen witte in tomatensaus blik/glas |
|  |  |  | 660 | Beans brown tinned | Bonen bruine blik/glas |
|  |  |  | 968 | Beans white/brown boiled | Bonen witte/bruine gekookt |
|  |  |  | 972 | Peas green boiled | Erwten groene gekookt |
|  |  |  | 1095 | Peas chick boiled | Erwten kikker- gekookt |
|  |  |  |  |  |  |
| **4** |  |  |  | **FRUITS, NUTS AND SEEDS** |  |
| **4** | **1** |  |  | **FRUITS** |  |
|  |  |  | 33 | Raisins dried | Rozijnen gedroogd |
|  |  |  | 147 | Apple without skin average | Appel z schil gem |
|  |  |  | 148 | Strawberries | Aardbeien |
|  |  |  | 149 | Apricots with skin | Abrikozen m schil |
|  |  |  | 150 | Pineapple | Ananas |
|  |  |  | 151 | Banana | Banaan |
|  |  |  | 152 | Blueberries | Bessen blauwe |
|  |  |  | 153 | Redcurrants | Bessen rode |
|  |  |  | 154 | Blackcurrants | Bessen zwarte |
|  |  |  | 155 | Bilberries | Bessen bos- |
|  |  |  | 157 | Blackberries | Bramen |
|  |  |  | 158 | Lemon | Citroen |
|  |  |  | 159 | Cranberries | Cranberries |
|  |  |  | 160 | Grapes with skin average | Druiven m schil gem |
|  |  |  | 161 | Raspberries | Frambozen |
|  |  |  | 162 | Grapefruit | Grapefruit |
|  |  |  | 163 | Cherries | Kersen |
|  |  |  | 164 | Gooseberries | Bessen kruis- |
|  |  |  | 165 | Mandarin | Mandarijn |
|  |  |  | 166 | Melon netted | Meloen net- |
|  |  |  | 168 | Pear without skin | Peer z schil |
|  |  |  | 169 | Peach without skin | Perzik z schil |
|  |  |  | 170 | Plums with skin | Pruimen m schil |
|  |  |  | 171 | Orange | Sinaasappel |
|  |  |  | 172 | Fruit fresh citrus average | Fruit vers citrus- gem |
|  |  |  | 173 | Fruit fresh average excluding citrus | Fruit vers excl citrus- gem |
|  |  |  | 174 | Strawberries in syrup tinned | Aardbeien op siroop blik/glas |
|  |  |  | 175 | Apricots dried | Abrikozen gedroogd |
|  |  |  | 176 | Apricots in syrup tinned | Abrikozen op siroop blik/glas |
|  |  |  | 177 | Pineapple in syrup tinned | Ananas op siroop blik/glas |
|  |  |  | 179 | Apple sauce tinned | Appelmoes blik/glas |
|  |  |  | 180 | Billberries in syrup tinned | Bessen bos- op siroop blik/glas |
|  |  |  | 181 | Dates candied | Dadels gekonfijt |
|  |  |  | 182 | Raspberries in syrup tinned | Frambozen op siroop blik/glas |
|  |  |  | 184 | Cherries in syrup tinned | Kersen op siroop blik/glas |
|  |  |  | 185 | Currants dried | Krenten gedroogd pak |
|  |  |  | 186 | Tangerines in syrup tinned | Mandarijnen op siroop blik/glas |
|  |  |  | 188 | Pears in syrup tinned | Peren op siroop blik/glas |
|  |  |  | 189 | Peaches in syrup tinned | Perziken op siroop blik/glas |
|  |  |  | 190 | Prunes dried | Pruimen gedroogd |
|  |  |  | 191 | Plums in syrup tinned | Pruimen op siroop blik/glas |
|  |  |  | 192 | Fruit mixed dried | Tuttifrutti gedroogd |
|  |  |  | 193 | Figs dried | Vijgen gedroogd |
|  |  |  | 538 | Rhubarb puree with sugar | Rabarbermoes m suiker |
|  |  |  | 692 | Mango | Mango |
|  |  |  | 693 | Papaya | Papaja |
|  |  |  | 875 | Apple with skin average | Appel m schil gem |
|  |  |  | 1010 | Figs fresh | Vijgen vers |
|  |  |  | 1056 | Kiwi fruit | Kiwi |
|  |  |  | 1057 | Kaki / Sharon fruit | Kaki / Sharonvrucht |
|  |  |  | 1074 | Passion fruit | Passievrucht |
|  |  |  | 1090 | Lychee | Lychee |
|  |  |  | 1105 | Melon water | Meloen water- |
|  |  |  | 1106 | Melon honeydew | Meloen suiker- |
|  |  |  | 1116 | Melon in syrup canned | Meloen op siroop blik/glas |
|  |  |  | 1182 | Apple sauce without sugar tinned | Appelmoes z suiker blik/glas |
|  |  |  | 1812 | Nectarine | Nectarine |
|  |  |  | 1887 | Dates fresh | Dadels vers |
|  |  |  | 1906 | Fruit fresh average including citrus | Fruit vers incl citrus- gem |
|  |  |  | 2379 | Raisins soaked in water | Rozijnen geweekt |
|  |  |  | 2685 | Apricots dried and soaked | Abrikozen gedroogde geweekt |
|  |  |  | 2686 | Apple sauce without sugar with sweetener tinned | Appelmoes z suiker m zoetstof blik/glas |
|  |  |  | 2748 | Pear with skin | Peer m schil |
|  |  |  | 2843 | Pineapple in own juice tinned | Ananas op eigen sap blik/glas |
|  |  |  | 2956 | Prunes dried soaked in water | Pruimen gedroogd geweekt |
| **4** | **2** |  |  | **NUTS AND SEEDS (AND NUT SPREAD)** | |
|  |  |  | 198 | Almonds blanched unsalted | Noten amandelen z vliesje ongezouten |
|  |  |  | 199 | Cashew nuts unsalted | Noten cashew- ongezouten |
|  |  |  | 200 | Hazelnuts unsalted | Noten hazel- ongezouten |
|  |  |  | 201 | Chestnuts | Kastanjes |
|  |  |  | 202 | Coconut meat fresh | Kokosnootvlees |
|  |  |  | 203 | Brazil nuts unsalted | Noten para- ongezouten |
|  |  |  | 206 | Walnuts unsalted | Noten wal- ongezouten |
|  |  |  | 207 | Nuts mixed unsalted | Noten gemengd ongezouten |
|  |  |  | 455 | Peanut butter | Pindakaas |
|  |  |  | 541 | Peanut butter with nut pieces | Pindakaas m stukjes noot |
|  |  |  | 546 | Peanuts coated | Noten borrel- |
|  |  |  | 838 | Sesame seeds | Sesamzaad |
|  |  |  | 867 | Linseeds | Lijnzaad |
|  |  |  | 872 | Sunflower seeds | Zonnebloempitten |
|  |  |  | 876 | Peanuts salted | Noten pinda's gezouten |
|  |  |  | 1461 | Sesame paste tahin | Pasta sesam- tahin |
|  |  |  | 1895 | Pecan nuts unsalted | Noten pecan- ongezouten |
|  |  |  | 1896 | Pistachio nuts salted | Noten pistache- gezouten |
|  |  |  | 1935 | Nuts mixed salted | Noten gemengd gezouten |
|  |  |  | 2048 | Peanuts dry roasted | Noten pinda's dry roasted |
|  |  |  | 2176 | Pine nuts | Pijnboompitten |
|  |  |  | 2345 | Peanuts sugar coated | Noten pinda's suiker- |
|  |  |  | 2367 | Peanut butter light | Pindakaas light |
|  |  |  | 2806 | Pumpkin seeds | Pompoenpitten |
|  |  |  | 2844 | Nuts macadamia | Noten macadamia |
| **4** | **3** |  |  | **MIXED FRUITS** |  |
|  |  |  | 179 | Apple sauce tinned | Appelmoes blik/glas |
|  |  |  | 183 | Fruit cocktail in syrup tinned | Fruitcocktail op siroop blik/glas |
|  |  |  | 192 | Fruit mixed dried | Tuttifrutti gedroogd |
|  |  |  | 205 | Mixed nuts and raisins | Studentenhaver |
|  |  |  | 1764 | Infant food fruit 12 months | Babyvoeding fruit 12 mnd |
|  |  |  | 1906 | Fruit fresh average including citrus | Fruit vers incl citrus- gem |
|  |  |  | 2845 | Fruitsnack Fruit'n fun multi fruit | Fruitsnack Fruit'n fun multifruit |
|  |  |  | 2875 | Fruitsnack Knijpfruit compote | Fruitsnack Knijpfruit/Slurpfruit |
|  |  |  | 2957 | Fruit mixed dried soaked in water | Tuttifrutti gedroogd geweekt |
| **4** | **4** |  |  | **OLIVES** |  |
|  |  |  | 137 | Olives tinned/glass | Olijven blik/glas |
|  |  |  |  |  |  |
| **5** |  |  |  | **DAIRY PRODUCTS** |  |
| **5** | **1** |  |  | **MILK** |  |
|  |  |  | 270 | Milk raw | Melk rauwe |
|  |  |  | 272 | Milk chocolate-flavoured whole | Melk chocolade- volle |
|  |  |  | 279 | Milk whole | Melk volle |
|  |  |  | 280 | Coffee creamer whole | Melk koffie- volle |
|  |  |  | 281 | Milk full fat condensed with sugar tinned | Melk volle gecondenseerd m suiker blik |
|  |  |  | 285 | Coffee creamer semi-skimmed | Melk koffie- halfvolle |
|  |  |  | 286 | Milk semi-skimmed | Melk halfvolle |
|  |  |  | 289 | Buttermilk | Melk karne- |
|  |  |  | 294 | Milk skimmed | Melk magere |
|  |  |  | 295 | Milk skimmed dried | Melkpoeder magere |
|  |  |  | 296 | Milk whole dried | Melkpoeder volle |
|  |  |  | 479 | Buttermilk with fruit | Melk karne- m vruchten |
|  |  |  | 1719 | Milk semi-skimmed enriched with calcium | Melk halfvolle verrijkt m calcium |
|  |  |  | 2172 | Nutrilon toddler milk p 100 ml | Nutrilon Peuter-Groei-melk p 100 ml Nutricia |
|  |  |  | 2240 | Milk goats whole | Melk geiten- volle |
|  |  |  | 2259 | Dairy drink Milk&Fruit light | Zuiveldrank Milk&Fruit light |
|  |  |  | 2309 | Milkdrink half fat Becel pro-activ | Melkdrank halfvolle- Becel pro-activ |
|  |  |  | 2496 | Dairy drink Campina fruitmilk | Zuiveldrank Fruitmelk Campina |
|  |  |  | 2725 | Milkdrink skimmed milk Becel pro-activ | Melkdrank magere- Becel pro-activ |
| **5** | **2** |  |  | **MILK BEVERAGES** |  |
|  |  |  | 272 | Milk chocolate-flavoured whole | Melk chocolade- volle |
|  |  |  | 273 | Milk chocolate-flavoured skimmed | Melk chocolade- magere |
|  |  |  | 279 | Milk whole | Melk volle |
|  |  |  | 294 | Milk skimmed | Melk magere |
|  |  |  | 479 | Buttermilk with fruit | Melk karne- m vruchten |
|  |  |  | 657 | Yoghurt drink | Yoghurtdrank |
|  |  |  | 862 | Milkshake | Milkshake |
|  |  |  | 1464 | Milk chocolate-flavoured semi-skimmed | Melk chocolade- halfvolle |
|  |  |  | 1970 | Milk chocolate-flavoured Chocomel light | Melk chocolade- m zoetstof Chocomel light |
|  |  |  | 2052 | Dairy drink Milk&Fruit strawberry-cherry | Zuiveldrank Milk&Fruit aardbei-kers |
|  |  |  | 2053 | Dairy drink Milk&Fruit orange | Zuiveldrank Milk&Fruit sinaasappel |
|  |  |  | 2259 | Dairy drink Milk&Fruit light | Zuiveldrank Milk&Fruit light |
|  |  |  | 2495 | Milk chocolate-flavoured with semi-skimmed milk and sweet cocoa | Melk chocolade- m hv melk en cacao gezoet |
|  |  |  | 2496 | Dairy drink Campina fruitmilk | Zuiveldrank Fruitmelk Campina |
|  |  |  | 2497 | Milkshake McDonald | Milkshake McDonald |
|  |  |  | 2500 | Milk chocolate-flavoured with semi-skimmed milk and Nesquik plus choc | Melk chocolade- van hv melk en Nesquik plus choc |
|  |  |  | 2760 | Hot chocolate from vending machine | Melk chocolade- automaat |
|  |  |  | 2835 | Coffee iced | IJskoffie |
|  |  |  | 2917 | Dairy drink Milk&Fruit mango | Zuiveldrank Milk&Fruit mango |
|  |  |  | 3004 | Milk chocolate-flavoured with sweetener Optimel | Melk chocolade- m zoetstof Optimel |
| **5** | **3** |  |  | **YOGHURT** |  |
|  |  |  | 278 | Yoghurt whole | Yoghurt volle |
|  |  |  | 284 | Yoghurt skimmed with fruit | Yoghurt magere m vruchten |
|  |  |  | 300 | Yoghurt whole Bulgarian | Yoghurt volle Bulgaarse |
|  |  |  | 301 | Yoghurt skimmed | Yoghurt magere |
|  |  |  | 657 | Yoghurt drink | Yoghurtdrank |
|  |  |  | 863 | Yoghurt whole with fruit | Yoghurt volle m vruchten |
|  |  |  | 917 | Fromage frais semi-skimmed with fruit | Kwark vruchten- halfvolle |
|  |  |  | 1502 | Yoghurt semi-skimmed | Yoghurt halfvolle |
|  |  |  | 1721 | Yoghurt vanilla semi-skimmed | Yoghurt vanille- halfvolle |
|  |  |  | 1813 | Yakult | Yakult |
|  |  |  | 1829 | Yoghurt semi-skimmed Vifit plain | Yoghurt halfvolle Vifit naturel |
|  |  |  | 1831 | Yoghurt drink Vifit plain | Yoghurtdrank Vifit naturel |
|  |  |  | 1832 | Yoghurt drink Vifit fruit | Yoghurtdrank Vifit vruchten |
|  |  |  | 1833 | Yoghurt skimmed with fruit/vanilla with sweetener Optimel | Yoghurt magere m vruchten/vanille m zoetstof Optimel |
|  |  |  | 1834 | Yoghurt drink with sweetener Optimel | Yoghurtdrank m zoetstof Optimel |
|  |  |  | 2023 | Yoghurt drink Yoki drink with sweetener | Yoghurtdrank Yoki drink m zoetstof |
|  |  |  | 2038 | Yoghurt drink Yomild drink plain | Yoghurtdrank Yomild drink naturel |
|  |  |  | 2039 | Yoghurt drink Yoki drink | Yoghurtdrank Yoki drink |
|  |  |  | 2241 | Yoghurt snack Breaker | Yoghurtsnack Breaker |
|  |  |  | 2243 | Yoghurt snack Breaker Light | Yoghurtsnack Breaker Light |
|  |  |  | 2244 | Yoghurt cream- with fruit | Yoghurt room- m vruchten |
|  |  |  | 2245 | Yoghurt skimmed with fruit with sweetener Vitalinea | Yoghurt magere m vruchten m zoetstof Vitalinea |
|  |  |  | 2247 | Fromage frais yoghurt with fruit | Kwarkyoghurt m vruchten |
|  |  |  | 2253 | Yoghurt drink Vifit fruit light | Yoghurtdrank Vifit vruchten light |
|  |  |  | 2254 | Yoghurt drink with sweetener | Yoghurtdrank m zoetstof |
|  |  |  | 2255 | Yoghurt drink Fristi with sweetener | Yoghurtdrank Fristi m zoetstof |
|  |  |  | 2256 | Yoghurt drink Yomild drink fruit | Yoghurtdrank Yomild drink vruchten |
|  |  |  | 2257 | Breakfast drink Goede Morgen original | Drinkontbijt Goede Morgen original |
|  |  |  | 2258 | Actimel | Actimel |
|  |  |  | 2265 | Yakult light | Yakult light |
|  |  |  | 2278 | Yoghurt whole with fruit/muesli Activia | Yoghurt volle m vruchten/muesli Activia |
|  |  |  | 2308 | Yoghurt drink Becel pro-activ | Yoghurtdrank Becel pro-activ |
|  |  |  | 2310 | Yoghurt product Becel pro-activ | Yoghurtproduct Becel pro-activ |
|  |  |  | 2501 | Yoghurt semi-skimmed with fruit | Yoghurt halfvolle m vruchten |
|  |  |  | 2502 | Yoghurt drink Topvit with sweetener | Yoghurtdrank Topvit m zoetstof |
|  |  |  | 2503 | Yoghurt Greek full fat | Yoghurt Griekse volle |
|  |  |  | 2597 | Yoghurt drink Optifit | Yoghurtdrank Optifit |
|  |  |  | 2604 | Optimel control | Optimel control |
|  |  |  | 2655 | Yoghurt 0% fat with fruit Activia | Yoghurt 0% vet m vruchten Activia |
|  |  |  | 2689 | Actimel light | Actimel light |
|  |  |  | 2822 | Yoghurt skimmed with fruit with sweetener | Yoghurt magere m vruchten m zoetstof |
|  |  |  | 2823 | Yoghurt whole stracciatella | Yoghurt volle stracciatella |
|  |  |  | 2853 | Yoghurt with fruit Mona Boordevol | Yoghurt m vruchten Mona Boordevol |
|  |  |  | 2935 | Yoghurt whole plain Activia | Yoghurt volle naturel Activia |
|  |  |  | 2936 | Breakfast drink Activia Start | Drinkontbijt Activia Start |
|  |  |  | 2940 | Breakfast drink Breaker Breakfast | Drinkontbijt Breaker Breakfast |
|  |  |  | 2941 | Breakfast drink Goede Morgen fruit | Drinkontbijt Goede Morgen fruit |
|  |  |  | 3005 | Breakfast drink Goede Morgen light | Drinkontbijt Goede Morgen light |
| **5** | **4** |  |  | **FROMAGE BLANC, PETITS SUISSES** |  |
|  |  |  | 305 | Fromage frais skimmed | Kwark magere |
|  |  |  | 306 | Fromage frais semi-skimmed | Kwark halfvolle |
|  |  |  | 307 | Fromage frais whole | Kwark volle |
|  |  |  | 654 | Cheese cottage | Kaas hüttenkäse |
|  |  |  | 917 | Fromage frais semi-skimmed with fruit | Kwark vruchten- halfvolle |
|  |  |  | 931 | Fromage frais skimmed with fruit | Kwark vruchten- magere |
|  |  |  | 2246 | Fromage frais skimmed with fruit with sw | Kwark magere m vruchten/vanille m zoetst Optimel |
|  |  |  | 2247 | Fromage frais yoghurt with fruit | Kwarkyoghurt m vruchten |
|  |  |  | 2248 | Fromage frais with fruit Danoontje | Kwark vruchten- Danoontje |
|  |  |  | 2504 | Fromage frais whole with fruit | Kwark vruchten- volle |
|  |  |  | 2853 | Yoghurt with fruit Mona Boordevol | Yoghurt m vruchten Mona Boordevol |
| **5** | **5** |  |  | **CHEESES (INCLUDING FRESH CHEESES)** | |
|  |  |  | 304 | Cheese Swiss dried | Kaas strooi- Zwitserse |
|  |  |  | 511 | Cheese Edam 40+ | Kaas Edammer 40+ |
|  |  |  | 512 | Cheese processed rindless 40+ | Kaas 40+ korstloze |
|  |  |  | 513 | Cheese Gouda 48+ average | Kaas Goudse 48+ gem |
|  |  |  | 514 | Cheese 20+ Leidse with cumin/Fries clove | Kaas 20+ Leidse/Friese nagel- |
|  |  |  | 515 | Cheese spread 48+ | Kaas smeer- volvet 48+ |
|  |  |  | 516 | Cheese spread 40+ | Kaas smeer- 40+ |
|  |  |  | 517 | Cheese spread 20+ | Kaas smeer- 20+ |
|  |  |  | 556 | Cheese Camembert 45+ | Kaas Camembert 45+ |
|  |  |  | 593 | Cheese Brie 50+ | Kaas Brie 50+ |
|  |  |  | 654 | Cheese cottage | Kaas hüttenkäse |
|  |  |  | 714 | Cheese Roquefort | Kaas Roquefort |
|  |  |  | 715 | Cheese cream 60+ | Kaas room- 60+ |
|  |  |  | 716 | Cheese Saint Paulin/Port Salut | Kaas Saint Paulin/Port Salut |
|  |  |  | 718 | Cheese Parmesan | Kaas Parmezaanse |
|  |  |  | 719 | Cheese cream soft Mon Chou | Kaas room- zachte Mon Chou |
|  |  |  | 721 | Cheese Limburger | Kaas Limburgse |
|  |  |  | 722 | Cheese Gruyere | Kaas Gruyere |
|  |  |  | 724 | Cheese Emmenthaler | Kaas Emmentaler |
|  |  |  | 725 | Cheese Cheddar | Kaas Cheddar |
|  |  |  | 726 | Cheese Bluefort | Kaas Bluefort |
|  |  |  | 728 | Cheese cream soft Boursin | Kaas room- zachte Boursin |
|  |  |  | 804 | Cheese sheep fresh | Kaas schapen- vers |
|  |  |  | 881 | Cheese 48+ low salt | Kaas 48+ minder zout |
|  |  |  | 882 | Cheese Kernhem 60+ | Kaas Kernhemmer 60+ |
|  |  |  | 883 | Cheese Amsterdam 48+ | Kaas Amsterdammer 48+ |
|  |  |  | 1103 | Cheese spread 30+ | Kaas smeer- 30+ |
|  |  |  | 1104 | Cheese smoked | Kaas rook- |
|  |  |  | 1108 | Cheese Rambol | Kaas Rambol |
|  |  |  | 1110 | Cheese Stilton | Kaas Stilton |
|  |  |  | 1111 | Cheese Camembert 30+ | Kaas Camembert 30+ |
|  |  |  | 1112 | Cheese raw milk 48+ | Kaas rauwmelkse 48+ |
|  |  |  | 1113 | Cheese sodium reduced 48+ | Kaas 48+ Na- |
|  |  |  | 1302 | Cheese cream soft Paturain | Kaas room- zachte Paturain |
|  |  |  | 1382 | Cheese 30+ | Kaas 30+ |
|  |  |  | 1487 | Cheese Brie 60+ | Kaas Brie 60+ |
|  |  |  | 1489 | Cheese fresh light 8% fat | Kaas verse light 8% vet |
|  |  |  | 1650 | Cheese goat fresh | Kaas geiten- verse |
|  |  |  | 1723 | Cheese 20+ | Kaas 20+ |
|  |  |  | 1724 | Cheese 50+ | Kaas 50+ |
|  |  |  | 1725 | Cheese Leerdammer/Maasdammer 45+ | Kaas Leerdammer/Maasdammer 45+ |
|  |  |  | 1726 | Cheese 40+ Leiden with cumin/Fries clove | Kaas 40+ Leidse/Friese nagel- |
|  |  |  | 1727 | Cheese 20+ rindless | Kaas 20+ korstloze |
|  |  |  | 1809 | Cheese 45+ | Kaas 45+ |
|  |  |  | 1893 | Cheese Bel Paese | Kaas Bel Paese |
|  |  |  | 1939 | Cheese Gorgonzola | Kaas Gorgonzola |
|  |  |  | 1955 | Cheese Mozzarella | Kaas Mozzarella |
|  |  |  | 2264 | Cheese 10+ | Kaas 10+ |
|  |  |  | 2516 | Cheese spread Eru Balans | Kaas smeer- Balans Eru |
|  |  |  | 2517 | Cheese spread Eru kids | Kaas smeer- kids Eru |
|  |  |  | 2518 | Cheese goat hard | Kaas geiten- hard |
|  |  |  | 2678 | Dairy spread plain/herbs | Zuivelspread naturel/kruiden |
|  |  |  | 2679 | Dairy spread plain/herbs light | Zuivelspread naturel/kruiden light |
|  |  |  | 2824 | Cheese 30+ low salt | Kaas 30+ minder zout |
|  |  |  | 2925 | Mascarpone cheese | Kaas Mascarpone |
|  |  |  | 2995 | Cheese spread 45+ | Kaas smeer- 45+ |
| **5** | **6** |  |  | **CREAM DESSERTS, PUDDINGS (MILK BASED)** | |
|  |  |  | 276 | Custard chocolate whole | Vla chocolade- volle |
|  |  |  | 282 | Custard vanilla whole | Vla vanille- volle |
|  |  |  | 284 | Yoghurt skimmed with fruit | Yoghurt magere m vruchten |
|  |  |  | 288 | Porridge oatmeal | Pap havermout- |
|  |  |  | 298 | Porridge rice | Pap rijste- |
|  |  |  | 477 | Custard vanilla skimmed | Vla vanille- magere |
|  |  |  | 478 | Custard chocolate skimmed | Vla chocolade- magere |
|  |  |  | 532 | Pudding chocolate | Pudding chocolade- |
|  |  |  | 605 | Porridge barley with raisins Bessola | Watergruwel Bessola |
|  |  |  | 736 | Pudding vanilla | Pudding vanille- |
|  |  |  | 767 | Mousse chocolate | Mousse chocolade- |
|  |  |  | 786 | Pudding chipolata | Pudding chipolata- |
|  |  |  | 912 | Pudding raspberries with currant sauce | Pudding frambozen- m bessensaus |
|  |  |  | 915 | Chocolate pudding with cream sauce | Pudding chocolade- m roomsaus |
|  |  |  | 917 | Fromage frais semi-skimmed with fruit | Kwark vruchten- halfvolle |
|  |  |  | 933 | Pudding caramel | Pudding caramel- |
|  |  |  | 938 | Pudding semolina with red currant sauce | Pudding griesmeel- m rode bessensap |
|  |  |  | 940 | Pudding vanilla with strawberry sauce | Pudding vanille- m aardbeiensaus |
|  |  |  | 1008 | Dairy dessert with cream average | Toetje m room |
|  |  |  | 1469 | Cream custard | Room banketbakkers- |
|  |  |  | 1720 | Custard several flavours whole | Vla volle overige smaken |
|  |  |  | 1721 | Yoghurt vanilla semi-skimmed | Yoghurt vanille- halfvolle |
|  |  |  | 1722 | Porridge semolina | Pap griesmeel- |
|  |  |  | 1957 | Custard with whipped cream | Vla slagroom- |
|  |  |  | 1958 | Porrdige buttermilk groats | Pap karnemelkse gorte- |
|  |  |  | 2244 | Yoghurt cream- with fruit | Yoghurt room- m vruchten |
|  |  |  | 2267 | Custard skimmed with sweetener Optimel | Vla magere m zoetstof Optimel |
|  |  |  | 2269 | Pudding low fat with sugar | Pudding light |
|  |  |  | 2270 | Mousse chocolate light | Mousse chocolade- light |
|  |  |  | 2371 | Tiramisu | Tiramisu |
|  |  |  | 2501 | Yoghurt semi-skimmed with fruit | Yoghurt halfvolle m vruchten |
|  |  |  | 2519 | Custard seni-skimmed all flavours | Vla halfvolle alle smaken |
|  |  |  | 2520 | Pudding airy average | Pudding luchtige gem |
|  |  |  | 2521 | Porridge whole milk with flour Lammetjespap | Pap lammetjes- bereid m volle melk |
|  |  |  | 2522 | Custard 2 flavours with syrup vlaflip Campina | Vlaflip dubbel- Campina |
|  |  |  | 2523 | Custard vanilla whole with chocolate balls Friesche Vlag | Vla Efteling knibbel knabbel Friesche Vlag |
|  |  |  | 2536 | Yoghurt & custard Campina | Yoghurt & vla Campina |
|  |  |  | 2537 | Custard with yoghurt and syrup vlaflip Campina | Vlaflip Campina |
|  |  |  | 2538 | Pudding home-made with semi-skimmed milk | Pudding huish bereid m halfvolle melk |
|  |  |  | 2539 | Custard soft and airy Campina | Vla zacht en luchtig Campina |
|  |  |  | 2822 | Yoghurt skimmed with fruit with sweetener | Yoghurt magere m vruchten m zoetstoffen |
| **5** | **7** |  |  | **DAIRY AND NON DAIRY CREAMS** |  |
| **5** | **7** | **1** |  | **DAIRY CREAMS** |  |
|  |  |  | 299 | Cream whipping | Room slag- onbereid |
|  |  |  | 812 | Cream sour | Room zure |
|  |  |  | 1503 | Cream 25% fat | Room slank- 25% vet onbereid |
|  |  |  | 1791 | Cream whipped with sugar canned | Room spuitbus |
|  |  |  | 1808 | Crème fraîche | Crème fraîche |
|  |  |  | 1916 | Cream whipped with added sugar | Room slag- geklopt m suiker |
|  |  |  | 2268 | Crème fraîche half fat | Crème fraîche halfvol |
|  |  |  | 2275 | Cream cooking | Room kook- |
|  |  |  | 2276 | Cream cooking light | Room kook- light |
| **5** | **7** | **2** |  | **NON DAIRY CREAMS** |  |
|  |  |  | 555 | Topping dessert with sugar whipped KlopKlop | Topping m suiker opgeklopt Klop-Klop |
|  |  |  | 2262 | Cream based on vegetable oil Cuisine Alpro | Cuisine Alpro |
|  |  |  | 2279 | Cream type prod Blue Band Finesse | Blue Band Finesse voor koken |
| **5** | **8** |  |  | **MILK FOR COFFEE AND CREAMERS** |  |
|  |  |  | 271 | Coffee creamer with pufa Becel | Melk koffie- mov verrijkt Becel |
|  |  |  | 274 | Coffee creamer powder | Creamer koffie- |
|  |  |  | 275 | Coffee creamer semi-skimmed with vegetable oil Nutroma | Melk koffie- halfvolle plantaardig Nutroma |
|  |  |  | 277 | Milk skimmed condensed with sugar tinned | Melk magere gecondenseerd m suiker blik |
|  |  |  | 280 | Coffee creamer whole | Melk koffie- volle |
|  |  |  | 285 | Coffee creamer semi-skimmed | Melk koffie- halfvolle |
|  |  |  | 292 | Coffee creamer skimmed | Melk koffie- magere |
|  |  |  | 293 | Coffee creamer 20% fat | Room koffie- |
|  |  |  | 554 | Coffee creamer powder Licht en Romig | Creamer koffie- Licht en Romig |
|  |  |  | 1954 | Coffee creamer Balance | Melk koffie- Balance |
|  |  |  | 2690 | Coffee creamer low fat powder | Creamer koffie- verlaagd vetgehalte |
|  |  |  |  |  |  |
| **6** |  |  |  | **CEREALS AND CEREAL PRODUCTS** |  |
| **6** | **1** |  |  | **FLOUR, FLAKES, STARCHES, SEMOLINA USED AS FLOUR** | |
|  |  |  | 78 | Semolina | Meel gries- |
|  |  |  | 80 | Flour rice | Bloem rijste- |
|  |  |  | 215 | Corn flour | Maizena |
|  |  |  | 220 | Flour wheat white 75% extraction | Bloem tarwe- patent |
|  |  |  | 222 | Flour wheat 50% extraction | Meel tarwe- |
|  |  |  | 223 | Binding agents averaged | Bindmiddel gem |
|  |  |  | 226 | Starch potato | Meel aardappel- |
|  |  |  | 231 | Wheat germ | Kiemen tarwe- |
|  |  |  | 530 | Flour for pancakes | Meel pannenkoek- |
|  |  |  | 590 | Wheat bran | Zemelen tarwe- |
|  |  |  | 696 | Flour maize | Meel mais- |
|  |  |  | 1020 | Flour wheat self-raising | Meel bak- zelfrijzend |
| **6** | **2** |  |  | **PASTA, RICE, OTHER GRAIN** |  |
|  |  |  | 368 | Chinese noodle dish Bami goreng tinned | Bami goreng blik |
|  |  |  | 646 | Spaghetti Bolognese frozen | Spaghetti Bolognaise diepvries |
|  |  |  | 658 | Rice white boiled | Rijst witte gekookt |
|  |  |  | 659 | Pasta plain average boiled | Pasta witte gem gekookt |
|  |  |  | 1014 | Rice brown boiled | Rijst zilvervlies- gekookt |
|  |  |  | 2157 | Pasta wholemeal boiled | Pasta volkoren gekookt |
|  |  |  | 2158 | Couscous boiled | Couscous gekookt |
|  |  |  | 2159 | Millet boiled | Gierst gekookt |
|  |  |  | 2271 | Pasta gluten-free raw | Pasta glutenvrij rauw |
|  |  |  | 2347 | Tortellini boiled | Tortellini gekookt |
|  |  |  | 2682 | Rice multi-grain boiled | Rijst meergranen- gekookt |
| **6** | **3** |  |  | **BREAD, CRISPBREAD, RUSKS** |  |
| **6** | **3** | **1** |  | **BREAD** |  |
|  |  |  | 230 | Roll white soft | Broodje wit zacht |
|  |  |  | 233 | Bread currant | Brood krenten- |
|  |  |  | 236 | Bread brown wheat | Brood tarwe- |
|  |  |  | 241 | Bread white milk based | Brood wit- melk |
|  |  |  | 242 | Bread rye dark | Brood rogge- donker |
|  |  |  | 243 | Bread rye light | Brood rogge- licht |
|  |  |  | 244 | Bread raisin | Brood rozijnen- |
|  |  |  | 246 | Bread wholemeal average | Brood volkoren- gem v fijn en grof |
|  |  |  | 248 | Bread white water based | Brood wit- water |
|  |  |  | 249 | Bread wheat malt Tarvo | Brood mout- Tarvo |
|  |  |  | 892 | Pizza mini frozen | Pizza mini diepvries |
|  |  |  | 1017 | Bread wheat rye wholemeal | Brood tarwerogge- volkoren |
|  |  |  | 1361 | Bread white Turkish | Brood wit- Turks |
|  |  |  | 1395 | Bread rye average | Brood rogge- gem |
|  |  |  | 1459 | Bread current wholemeal | Brood krenten- volkoren |
|  |  |  | 1534 | Pizza crossa crust frozen | Pizza m crossabodem diepvries |
|  |  |  | 2115 | Bread loaf gluten-free Glutafin | Brood glutenvrij Glutafin |
|  |  |  | 2277 | Bread Blue Band Goede Start white bread | Brood Blue Band Goede Start Witbrood |
|  |  |  | 2312 | Roti Surinam pancake | Roti |
|  |  |  | 2348 | Bread wholemeal with pumpkin seeds | Brood volkoren- m pompoenpitten |
|  |  |  | 2349 | Bread linseed | Brood lijnzaad- |
|  |  |  | 2350 | Bread multigrain average with seeds | Brood meergranen- m div zaden gem |
|  |  |  | 2351 | Bread white average milk/water based | Brood wit- gem van melk- en waterwit |
|  |  |  | 2352 | Bread currant with almond paste | Brood krenten- m spijs |
|  |  |  | 2353 | Bread brown/wholemeal with muesli | Brood muesli- obv meel/volkorenmeel |
|  |  |  | 2354 | Bread wholemeal with nuts | Brood noten- volkoren |
|  |  |  | 2355 | Bread white with sugar Suikerbrood | Brood suiker- wit |
|  |  |  | 2356 | Baguette with cheese-onion | Brood stok- kaas-uien |
|  |  |  | 2357 | Bread wholemeal with sunflower seeds | Brood volkoren- m zonnebloempitten |
|  |  |  | 2358 | Bread white with sunflower seeds | Brood wit- m zonnebloempitten |
|  |  |  | 2359 | Wrap/Tortilla | Wrap/Tortilla |
|  |  |  | 2382 | Bread brown with sunflower seeds | Brood tarwe- m zonnebloempitten |
|  |  |  | 2494 | Bread Blue Band Goede Start light brown | Brood Blue Band Goede Start Lichtbruin |
|  |  |  | 2702 | Bread C1000 Kids Wit | Brood C1000 Kids Wit |
|  |  |  | 2703 | Bread wholemeal with seeds | Brood volkoren- m diverse zaden |
|  |  |  | 2704 | Bread brown with seeds | Brood tarwe- m diverse zaden |
|  |  |  | 2707 | Bread corn with sunflower seeds | Brood mais- m zonnebloempitten |
|  |  |  | 2785 | Bread corn | Brood mais- |
|  |  |  | 2788 | Bread sourdough wholemeal | Brood zuurdesem- volkoren |
|  |  |  | 2789 | Bread ciabatta no filling | Brood wit- ciabatta ongevuld |
|  |  |  | 2790 | Bread pita white | Broodje wit pita |
|  |  |  | 2793 | Baguette white | Brood stok- wit |
|  |  |  | 2794 | Baguette brown | Brood stok- bruin |
|  |  |  | 2795 | Roll white hard | Broodje wit hard |
|  |  |  | 2796 | Roll brown hard | Broodje tarwe- hard |
|  |  |  | 2797 | Roll brown soft | Broodje tarwe- zacht |
|  |  |  | 2798 | Roll wholemeal soft | Broodje volkoren- zacht |
|  |  |  | 2803 | Bun currant/raisin | Bol krenten- |
|  |  |  | 2804 | Bun wholemeal with muesli | Bol muesli- meel/volkorenmeel |
|  |  |  | 2813 | Bread raisin/current average | Brood rozijnen-krenten gem |
|  |  |  | 2816 | Bread Tijger white | Brood tijger- wit |
|  |  |  | 2817 | Stollen with almond/imitat paste average | Stol m spijs gem m en z noten |
|  |  |  | 2821 | Bread brown with pumpkin seeds | Brood tarwe- m pompoenpitten |
|  |  |  | 2865 | Bread Omega- | Brood omega- |
|  |  |  | 2876 | Bread brioche | Brood brioche |
|  |  |  | 3007 | Bread low in carbohydrates | Brood koolhydraatarm |
| **6** | **3** | **2** |  | **CRISPBREAD, RUSKS** |  |
|  |  |  | 227 | Crispbakes Dutch | Beschuit |
|  |  |  | 228 | Crackers cream | Cracker cream- |
|  |  |  | 229 | Crispbread averaged | Knäckebröd gem |
|  |  |  | 230 | Roll white soft | Broodje wit zacht |
|  |  |  | 238 | Crackers matzes | Cracker tea- matses |
|  |  |  | 565 | Toast | Brood geroosterd toast |
|  |  |  | 596 | Crispbread gold-brown | Knäckebröd goudbruin |
|  |  |  | 655 | Crispbakes Dutch wholemeal | Beschuit volkoren |
|  |  |  | 975 | Crispbread sesame | Knäckebröd sesam |
|  |  |  | 976 | Crispbread light | Knäckebröd lichtgewicht |
|  |  |  | 1013 | Bread crumbs | Paneermeel |
|  |  |  | 1253 | Crispbread high fibre | Knäckebröd vezelrijk |
|  |  |  | 1312 | Crispbread Cracottes | Cracottes naturel |
|  |  |  | 1322 | Crispbread wholemeal Cracottes | Cracottes volkoren |
|  |  |  | 1352 | Crackers gluten-free Glutafin | Cracker glutenvrij Glutafin |
|  |  |  | 1481 | Rice cakes puffed | Wafel rijst- naturel |
|  |  |  | 1779 | Crispbread wholemeal | Knäckebröd volkoren |
|  |  |  | 2229 | Crackers VitaLU | Cracker VitaLU |
|  |  |  | 2230 | Crispbread Cracottes Vital | Cracottes Vital |
|  |  |  | 2231 | Rice cakes puffed with chocolate | Wafel rijst- m chocolade |
|  |  |  | 2360 | Rice cakes puffed with caramel | Wafel rijst- m caramel |
|  |  |  | 2406 | Biscuit children's average | Koekje kinder- gem |
|  |  |  | 2937 | Toast Melba plain | Toast Melba naturel |
|  |  |  | 2938 | Toast Melba other varieties | Toast Melba overige soorten |
| **6** | **4** |  |  | **BREAKFAST CEREALS** |  |
|  |  |  | 209 | Breakfast cereal Cornflakes Kellogg's | Ontbijtproduct Cornflakes Kellogg's |
|  |  |  | 213 | Oatmeal | Mout haver- |
|  |  |  | 225 | Breakfast cereal Brinta | Ontbijtproduct Brinta |
|  |  |  | 591 | Breakfast cereal All-Bran Plus Kellogg's | Ontbijtproduct All-Bran Plus Kellogg's |
|  |  |  | 653 | Breakfast cereal Rice Krispies Kellogg's | Ontbijtproduct Rice Krispies Kellogg's |
|  |  |  | 1023 | Flour rice instant Bambix | Bloem rijste- Bambix |
|  |  |  | 1338 | Breakfast prod Bambix Groei Ontbijt light wholemeal | Ontbijtprod Bambix Groei Ontbijt licht volkoren |
|  |  |  | 1991 | Breakfast prod Bambix Peuter Ontbijt 8 cereals | Ontbijtprod Bambix Peuter Ontbijt 8 granen |
|  |  |  | 1999 | Breakfast product Frosties Kellogg's | Ontbijtproduct Frosties Kellogg's |
|  |  |  | 2002 | Breakfast prod Coco Pops Kellogg's | Ontbijtproduct Choco Pops Kellogg's |
|  |  |  | 2004 | Breakfast cereal All-Bran Fruit 'n Fibre | Ontbijtproduct All-Bran Fruit 'n Fibre Kellogg's |
|  |  |  | 2005 | Breakfast product Special K Original Kellogg’s | Ontbijtproduct Special K Original Kellogg's |
|  |  |  | 2006 | Breakfast cereal Smacks Kellogg's | Ontbijtproduct Smacks Kellogg's |
|  |  |  | 2007 | Breakfast cereal Honey Loops Kellogg's | Ontbijtproduct Honey Loops Kellogg's |
|  |  |  | 2051 | Drink breakfast Brinta Wake Up | Drinkontbijt Brinta Wake Up |
|  |  |  | 2081 | Breakfast cereal Cornflakes | Ontbijtproduct Cornflakes |
|  |  |  | 2361 | Breakfast prod Albona 7-cereals-energy | Ontbijtproduct Albona 7-granen-energie ontbijt |
|  |  |  | 2366 | Muesli crunchy plain/with fruit | Muesli krokante naturel/m fruit |
|  |  |  | 2652 | Corn flakes Golden Bridge | Cornflakes Golden Bridge |
|  |  |  | 2675 | Muesli crunchy with nuts | Muesli krokante m noten |
|  |  |  | 2676 | Muesli crunchy with chocolate | Muesli krokante m chocolade |
|  |  |  | 2677 | Muesli crunchy with nuts and chocolate | Muesli krokante m noten en chocolade |
|  |  |  | 2683 | Breakfast cereal Chocos Kellogg's | Ontbijtproduct Chocos Kellogg's |
|  |  |  | 2684 | Breakfast product Special K chocolate Kellogg's | Ontbijtproduct Special K chocolade Kellogg's |
|  |  |  | 2705 | Breakfast prod All-Bran flakes Kellogg's | Ontbijtproduct All-Bran flakes Kellogg's |
|  |  |  | 2809 | Muesli with fruit | Muesli vruchten- |
|  |  |  | 2877 | Breakfast product Weetabix original | Ontbijtproduct Weetabix original |
| **6** | **5** |  |  | **SALTY BISCUITS, APERITIF BISCUITS, CRACKERS, …** | |
|  |  |  | 122 | Crisps potato average | Chips gem |
|  |  |  | 228 | Crackers cream | Cracker cream- |
|  |  |  | 264 | Biscuit salted average | Biscuit zoute |
|  |  |  | 265 | Puff pastry baked | Bladerdeeg bereid |
|  |  |  | 267 | Pretzel sticks | Pepsels |
|  |  |  | 269 | Prawn crackers | Kroepoek bereid |
|  |  |  | 546 | Peanuts coated | Noten borrel- |
|  |  |  | 565 | Toast | Brood geroosterd toast |
|  |  |  | 618 | Cocktail snacks Nibbits | Nibbits |
|  |  |  | 619 | Cocktail snacks Wokkels | Wokkels |
|  |  |  | 620 | Crisps potato straws average | Fritessticks gem |
|  |  |  | 630 | Popcorn puffed plain | Popcorn gepoft naturel |
|  |  |  | 1395 | Bread rye average | Brood rogge- gem |
|  |  |  | 1481 | Rice cakes puffed plain | Wafel rijst- naturel |
|  |  |  | 1505 | Crisps potato light unflavoured | Chips light naturel |
|  |  |  | 1699 | Biscuits and snacks cheesy averaged | Koekje kaas- gem |
|  |  |  | 1937 | Crisps tortilla unflavoured | Chips tortilla naturel |
|  |  |  | 1943 | Japanese rice cracker mix with peanuts | Japanse mix m pinda's |
|  |  |  | 2147 | Japanese rice cracker mix without peanuts | Japanse mix z pinda's |
|  |  |  | 2163 | Crisps maize Bugles | Bugles |
|  |  |  | 2173 | Crisps based on potato flour | Chips op basis van aardappelmeel |
|  |  |  | 2369 | Breadsticks | Soepstengel |
|  |  |  | 2370 | Cocktail snacks based on corn or wheat | Zoutje luchtig mais/tarwebasis |
|  |  |  | 2527 | Biscuit savoury Sultana | Biscuit hartig Sultana |
|  |  |  | 2528 | Croutons | Croutons |
|  |  |  | 2529 | Potato crisps oven baked | Chips oven- |
|  |  |  | 2653 | Rice cakes with spices | Wafel rijst- gekruid |
|  |  |  | 2706 | Cassave crackers | Chips/Kroepoek Cassave |
|  |  |  | 2923 | Crisps potato unflavoured | Chips naturel |
|  |  |  | 2924 | Crisps potato flavoured | Chips paprika ea smaken |
|  |  |  | 2926 | Crisps potato light flavoured | Chips light paprika ea smaken |
|  |  |  | 2927 | Crisps potato straws natural | Fritessticks naturel |
|  |  |  | 2928 | Crisps potato straws flavoured | Fritessticks paprika |
|  |  |  | 2929 | Crisps potato Lays Sensations flavoured | Chips Lays Sensations div smaken |
|  |  |  | 2937 | Toast Melba natural | Toast Melba naturel |
|  |  |  | 2938 | Toast Melba other varieties | Toast Melba overige soorten |
| **6** | **6** |  |  | **DOUGH AND PASTRY (PUFF, SHORT-CRUST, PIZZA)** | |
|  |  |  | 265 | Puff pastry baked | Bladerdeeg, bereid |
|  |  |  | 1914 | Dough for pizza and savoury pie | Brooddeeg (bodem voor pizza/hartige taart) |
|  |  |  |  |  |  |
| **7** |  |  |  | **MEAT AND MEAT PRODUCTS** |  |
| **7** | **1** |  |  | **FRESH MEAT** |  |
|  |  | 0 | 1434 | Minced beef/pork raw | Gehakt hoh rauw |
|  |  |  | 1568 | Minced beef/pork ball prepared with egg/crumbs | Gehaktbal hoh bereid |
|  |  |  | 1668 | Pork 5-14% fat raw average | Varkensvlees 5-14 g vet rauw gem |
|  |  |  | 2333 | Minced meat raw average | Gehakt rauw gem |
|  |  |  | 2334 | Minced meat beef/pork raw with egg/bread crumbs | Gehakt hoh m ei en paneermeel rauw |
|  |  |  | 2335 | Meat average raw excl liver | Vlees gem excl lever rauw |
|  |  |  | 2340 | Pork schnitzel breaded raw | Varkensschnitzel gepaneerd rauw |
| **7** | **1** | **1** |  | **BEEF** |  |
|  |  |  | 1400 | Beef rump steak raw | Runderbiefstuk rauw |
|  |  |  | 1401 | Beef tenderloin steak raw | Runderbiefstuk van de haas rauw |
|  |  |  | 1402 | Beef prime rib raw | Runderentrecote rauw |
|  |  |  | 1404 | Beef frying steak raw | Runderbaklappen rauw |
|  |  |  | 1405 | Minced beef raw | Gehakt runder- rauw |
|  |  |  | 1406 | Beef rib raw | Runderklapstuk rauw |
|  |  |  | 1408 | Beef stewing meat raw | Runderpoelet rauw |
|  |  |  | 1409 | Beef sirloin rolled raw | Runderrollade rauw |
|  |  |  | 1410 | Beef roast raw | Runderrosbief rauw |
|  |  |  | 1412 | Beef rib steak raw | Runderriblappen rauw |
|  |  |  | 1413 | Beef streaked/marbled raw | Runder doorregen lappen rauw |
|  |  |  | 1414 | Beef stewing steak raw | Rundersukadelappen rauw |
|  |  |  | 1415 | Beef steak tartare raw | Rundertartaar rauw |
|  |  |  | 1663 | Beef <5 g fat raw average | Rundvlees <5 g vet rauw gem |
|  |  |  | 2336 | Beef average raw | Rundvlees gem rauw |
|  |  |  | 2337 | Minced beef raw with egg and breadcrumbs | Gehakt runder- m ei en paneermeel rauw |
|  |  |  | 2338 | Beef for stewing averaged raw | Rundvlees om te stoven gem rauw |
|  |  |  | 2339 | Beef olives raw | Vink runder- rauw |
|  |  |  | 3033 | Beef T-bone steak raw | Runder T-bone steak rauw |
| **7** | **1** | **2** |  | **VEAL** |  |
|  |  |  | 1436 | Veal prime rib raw | Kalfsentrecote rauw |
|  |  |  | 1437 | Veal olive raw | Vink kalfs- rauw |
|  |  |  | 1673 | Veal <5g fat raw average | Kalfsvlees <5 g vet gem rauw |
|  |  |  | 3016 | Veal schnitzel not breaded raw | Kalfsschnitzel ongepaneerd rauw |
|  |  |  | 3017 | Minced veal raw | Gehakt kalfs- rauw |
|  |  |  | 3019 | Veal frying steak raw | Kalfslappen mager rauw |
| **7** | **1** | **3** |  | **PORK** |  |
|  |  |  | 1418 | Pork fillet raw | Varkensfiletlappen rauw |
|  |  |  | 1419 | Pork fricandeau part of leg raw | Varkensfricandeau rauw |
|  |  |  | 1420 | Pork chop raw | Varkenshamlappen rauw |
|  |  |  | 1421 | Minced pork raw | Gehakt varkens- rauw |
|  |  |  | 1422 | Pork tenderloin raw | Varkenshaas rauw |
|  |  |  | 1424 | Pork loin chop raw | Varkenshaaskarbonade rauw |
|  |  |  | 1425 | Pork shoulder chop raw | Varkensschouderkarbonade rauw |
|  |  |  | 1427 | Pork spare rib raw | Varkenskrabbetjes rauw |
|  |  |  | 1428 | Pork stir fry meat raw | Varkensnasivlees rauw |
|  |  |  | 1429 | Pork tenderloin medaillon raw | Varkensoester rauw |
|  |  |  | 1430 | Pork shoulder raw | Varkensschouderlappen rauw |
|  |  |  | 1431 | Kromesky meat filled raw | Vink sla- rauw |
|  |  |  | 1667 | Pork <5g fat raw average | Varkensvlees <5 g vet rauw gem |
|  |  |  | 1668 | Pork 5-14g fat raw average | Varkensvlees 5-14 g vet rauw gem |
|  |  |  | 1788 | Pork rib chop raw | Varkensribkarbonade rauw |
|  |  |  | 1790 | Pork schnitzel not breaded raw | Varkensschnitzel ongepaneerd rauw |
|  |  |  | 2340 | Pork schnitzel breaded raw | Varkensschnitzel gepaneerd rauw |
|  |  |  | 3024 | Pork stewing meat raw | Varkenspoelet rauw |
|  |  |  | 3027 | Pork shoarma seasoning raw | Shoarmavlees varkens- rauw |
|  |  |  | 3031 | Pork sparerib raw | Spareribs varkens- rauw |
| **7** | **1** | **4** |  | **MUTTON/LAMB** |  |
|  |  |  | 99 | Mutton >10g fat raw av | Schapenvlees >10 g vet rauw gem |
|  |  |  | 1444 | Minced lamb/mutton raw | Gehakt lams-/schapen- rauw |
|  |  |  | 1445 | Lamb chop raw | Lamskarbonade rauw |
|  |  |  | 1675 | Lamb >10g fat raw average | Lamsvlees >10 g vet rauw gem |
|  |  |  | 2057 | Lamb <10g fat raw av | Lamsvlees <10 g vet gem rauw |
|  |  |  | 2058 | Mutton <10g fat raw av | Schapenvlees <10 g vet gem rauw |
| **7** | **1** | **5** |  | **HORSE** |  |
|  |  |  | 95 | Horsemeat raw | Paardenvlees rauw |
| **7** | **1** | **6** |  | **GOAT** |  |
|  |  |  | 1900 | Goat meat raw average | Geitenvlees rauw gem |
| **7** | **2** |  |  | **POULTRY** |  |
|  |  | 0 | 1305 | Chicken without skin raw | Kip z vel rauw |
|  |  |  | 2089 | Ostrich, raw | Struisvogelvlees rauw |
| **7** | **2** | **1** |  | **CHICKEN, HEN** |  |
|  |  |  | 108 | Chicken with skin raw | Kip m vel rauw |
|  |  |  | 1305 | Chicken without skin raw | Kip z vel rauw |
|  |  |  | 1392 | Chicken fillet prepared | Kipfilet bereid |
|  |  |  | 1634 | Chicken fillet raw | Kipfilet rauw |
|  |  |  | 1642 | Chicken rolled raw | Kiprollade rauw |
|  |  |  | 2090 | Chicken drumstick with skin raw | Kip drumstick m vel rauw |
|  |  |  | 2341 | Chicken fillet breaded raw | Kipfilet gepaneerd rauw |
|  |  |  | 2342 | Chicken fillet in batter raw | Kipfilet omhuld m beslag rauw |
|  |  |  | 2364 | Chicken schnitzel breaded raw | Kipschnitzel gepaneerd rauw |
| **7** | **2** | **2** |  | **TURKEY, YOUNG TURKEY** |  |
|  |  |  | 330 | Turkey raw | Kalkoen rauw |
|  |  |  | 1936 | Turkey fillet raw | Kalkoenfilet rauw |
|  |  |  | 2364 | Chicken schnitzel raw | Kipschnitzel gepaneerd rauw |
| **7** | **2** | **3** |  | **DUCK** |  |
|  |  |  | 106 | Duck whole raw | Eend rauw |
| **7** | **2** | **4** |  | **GOOSE** |  |
| **7** | **2** | **5** |  | **RABBIT (DOMESTIC)** |  |
|  |  |  | 109 | Rabbit domesticated raw | Konijn tam rauw |
| **7** | **3** |  |  | **GAME** |  |
|  |  |  | 107 | Hare whole raw | Haas rauw |
|  |  |  | 339 | Venison raw | Ree wild rauw |
| **7** | **4** |  |  | **PROCESSED MEAT** |  |
|  |  |  | 319 | Corned beef | Cornedbeef |
|  |  |  | 322 | Sausage Dutch Frikandel deep-fried | Frikandel bereid |
|  |  |  | 324 | Sausage smoked cooked average | Worst rook- gekookt gem |
|  |  |  | 328 | Ham smoked raw | Ham rauwe |
|  |  |  | 333 | Liver ox boiled | Lever runder- gekookt (vleeswaar) |
|  |  |  | 334 | Liver pork boiled | Lever varkens- gekookt (vleeswaar) |
|  |  |  | 335 | Liver pate | Pastei lever- |
|  |  |  | 336 | Luncheon meat tinned | Luncheon meat blik |
|  |  |  | 338 | Beef salted cooked | Pekelvlees |
|  |  |  | 340 | Beef smoke-dried | Runderrookvlees |
|  |  |  | 343 | Bacon fat raw | Spek vers vet rauw |
|  |  |  | 344 | Processed meat products average | Vleeswaren gem |
|  |  |  | 566 | Sausage frankfurter tinned | Worst knak- blik/glas |
|  |  |  | 567 | Sausage luncheon meat | Worst boterham- |
|  |  |  | 568 | Black pudding | Worst bloed- |
|  |  |  | 638 | Salami sausage saveloy | Worst cervelaat- |
|  |  |  | 639 | Bacon rashers streaky | Spek ontbijt- |
|  |  |  | 640 | Liver sausage | Worst lever- |
|  |  |  | 641 | Bacon | Bacon |
|  |  |  | 642 | Pate | Pate |
|  |  |  | 643 | Pork side cured and smoked | Casselerrib gerookt/gekookt |
|  |  |  | 782 | Sausage spiced and smoked | Worst thee- |
|  |  |  | 783 | Sausage with smoked bacon-bits | Worst paling- |
|  |  |  | 784 | Ham lean boiled | Ham achter- |
|  |  |  | 785 | Ham shoulder medium fat boiled | Ham schouder- |
|  |  |  | 810 | Beef steak tartare spiced filet americ | Filet americain |
|  |  |  | 1152 | Salami | Worst salami |
|  |  |  | 1155 | Minced meat loaf fried | Gehakt gebraden (vleeswaar) |
|  |  |  | 1162 | Sausage cooked | Worst gekookte |
|  |  |  | 1238 | Liver sausage coarse hausmacher | Worst lever- hausmacher |
|  |  |  | 1239 | Liver paté sausage | Worst smeerlever- |
|  |  |  | 1306 | Chicken for soup with skin raw | Kip soep- m vel rauw |
|  |  |  | 1368 | Sausage dry salam Turkish | Worst droge salam Turks |
|  |  |  | 1391 | Bacon lean prepared | Spek mager bereid |
|  |  |  | 1417 | Sausage pork Braadworst raw | Varkensbraadworst rauw |
|  |  |  | 1421 | Minced pork raw | Gehakt varkens- rauw |
|  |  |  | 1432 | Bacon rasher raw | Speklap rauw |
|  |  |  | 1434 | Minced beef/pork raw | Gehakt hoh rauw |
|  |  |  | 1435 | Hamburger raw | Hamburger rauw |
|  |  |  | 1444 | Minced lamb/mutton raw | Gehakt lams-/schapen- rauw |
|  |  |  | 1544 | Beef rolled prepared | Runderrollade bereid |
|  |  |  | 1545 | Beef roast prepared | Runderrosbief bereid |
|  |  |  | 1558 | Pork loin chop prepared | Varkenshaaskarbonade bereid |
|  |  |  | 1568 | Minced beef/pork ball prepared with egg/crumbs | Gehaktbal hoh bereid |
|  |  |  | 1572 | Veal fricandeau prepared | Kalfsfricandeau bereid |
|  |  |  | 1641 | Chicken rolled prepared | Kiprollade bereid |
|  |  |  | 1643 | Chicken nuggets prepared in oven | Kipnuggets bereid in oven |
|  |  |  | 1671 | Pork 10-19 g fat prepared average | Varkensvlees 10-19 g vet bereid gem |
|  |  |  | 1675 | Lamb >10 g fat raw average | Lamsvlees >10 g vet rauw gem |
|  |  |  | 1771 | Liver pate/Berliner liver sausage | Leverkaas/berliner |
|  |  |  | 1772 | Pork fricandeau fried | Varkensfricandeau gebraden (vleeswaar) |
|  |  |  | 1773 | Brawn, pork pickled in vinegar | Zult zure |
|  |  |  | 1774 | Sausage tongue | Worst tongen- |
|  |  |  | 1776 | Ham lean grilled | Ham achter- gegrild |
|  |  |  | 1777 | Gammon boiled deboned | Ham been- |
|  |  |  | 1790 | Pork schnitzel not breaded raw | Varkensschnitzel ongepaneerd rauw |
|  |  |  | 1907 | Processed meat prod excl liver average | Vleeswaren excl leverproducten gem |
|  |  |  | 1908 | Processed meat prod <10 g fat excl liver average | Vleeswaren <10 g vet excl leverprod gem |
|  |  |  | 1909 | Sausage excl liver average | Worst excl leverproducten gem |
|  |  |  | 2300 | Sausage smoked lean cooked | Worst rook- magere gekookt |
|  |  |  | 2301 | Sausage smoked beef cooked | Worst rook- runder gekookt |
|  |  |  | 2302 | Sausage frankfurter lean tinned | Worst knak- magere blik/glas |
|  |  |  | 2303 | Sausage cooked lean | Worst gekookte magere |
|  |  |  | 2304 | Salami sausage saveloy lean | Worst cervelaat- magere |
|  |  |  | 2305 | Liver pate/Berliner liver sausage lean | Leverkaas/berliner magere |
|  |  |  | 2306 | Sausage luncheon meat lean | Worst boterham- magere |
|  |  |  | 2307 | Minced meat loaf fried lean | Gehakt gebraden magere (vleeswaar) |
|  |  |  | 2334 | Minced meat beef/pork raw with egg/bread crumbs | Gehakt hoh m ei en paneermeel rauw |
|  |  |  | 2340 | Pork schnitzel breaded raw | Varkensschnitzel gepaneerd rauw |
|  |  |  | 2342 | Chicken fillet in batter raw | Kipfilet omhuld m beslag rauw |
|  |  |  | 2344 | Pork filled with ham and cheese raw | Varkens cordon bleu rauw |
|  |  |  | 2362 | Sausage Dutch Frikandel frozen unprepared | Frikandel diepvries onbereid |
|  |  |  | 2363 | Minced meat filled with ham and cheese raw | Gehakt cordon bleu rauw |
|  |  |  | 2364 | Chicken schnitzel raw | Kipschnitzel gepaneerd rauw |
|  |  |  | 2365 | Chicken filled with ham and cheese raw | Kip cordon bleu rauw |
|  |  |  | 2381 | Paté spreadable Linera | Paté smeer- Linera |
|  |  |  | 2411 | Minced beef/ pork with vegetables raw | Gehakt hoh m groenten rauw |
|  |  |  | 2654 | Sandwich meat chicken | Kipfilet (vleeswaar) |
|  |  |  | 2768 | Sausage raw beef | Worst ossen- |
|  |  |  | 2826 | Chicken schnitzel satay breaded raw | Kipschnitzel saté gepaneerd rauw |
|  |  |  | 2827 | Pork schnitzel satay breaded raw | Varkensschnitzel saté gepaneerd rauw |
|  |  |  | 2836 | Sausage grill with cheese | Worst grill- met kaas |
|  |  |  | 2996 | Sausage Chorizo | Worst Chorizo |
|  |  |  | 2997 | Sausage grill | Worst grill- |
|  |  |  | 3001 | Sandwich meat turkey | Kalkoenfilet (vleeswaar) |
|  |  |  | 3002 | Bacon smoked katenspek | Spek katen- |
|  |  |  | 3003 | Sausage beef frankfurter type tinned | Worst knak- runder blik/glas |
|  |  |  | 3025 | Bacon lean smoked raw | Spek rook- mager rauw |
|  |  |  | 3030 | Sausage beef Braadworst raw | Runderbraadworst rauw |
| **7** | **5** |  |  | **OFFALS** |  |
|  |  |  | 475 | Liver chicken raw | Lever kippen- rauw |
|  |  |  | 1407 | Liver ox raw | Lever runder- rauw |
|  |  |  | 1426 | Liver pork raw | Lever varkens- rauw |
|  |  |  | 1902 | Kidney ox raw | Nier runder- rauw |
|  |  |  |  |  |  |
| **8** |  |  |  | **FISH AND SHELLFISH** |  |
| **8** | **1** |  |  | **FISH** |  |
|  |  |  | 115 | Fish medium fat > 2-10 g fat raw average | Vis matig vet >2-10 g vet gem rauw |
|  |  |  | 116 | Fish fat > 10 g fat raw average | Vis vet >10 g vet gem rauw |
|  |  |  | 347 | Kipper smoked | Bokking gerookt |
|  |  |  | 349 | Herring fillet in tomato sauce tinned | Haringfilet in tomatensaus blik |
|  |  |  | 350 | Herring salted | Haring gezouten |
|  |  |  | 355 | Sardines/pilchards in oil tinned | Sardines in olie blik |
|  |  |  | 602 | Salmon tinned | Zalm blik |
|  |  |  | 603 | Eel smoked | Paling gerookt |
|  |  |  | 815 | Fish fingers unprepared | Vissticks onbereid |
|  |  |  | 819 | Cod boiled | Kabeljauw gekookt |
|  |  |  | 820 | Cod raw | Kabeljauw rauw |
|  |  |  | 918 | Plaice boiled | Schol gekookt |
|  |  |  | 919 | Pollack boiled | Koolvis gekookt |
|  |  |  | 1096 | Salmon smoked | Zalm gerookt |
|  |  |  | 1100 | Herring pickled (sweet)sour | Haring in (zoet)zuur |
|  |  |  | 1586 | Mackerel fillet smoked | Makreelfilet gerookt |
|  |  |  | 1587 | Salmon raw | Zalm rauw |
|  |  |  | 1588 | Anchovy in oil tinned | Ansjovis in olie blik |
|  |  |  | 1589 | Tuna in oil tinned | Tonijn in olie blik |
|  |  |  | 1590 | Tuna in water tinned | Tonijn in water blik |
|  |  |  | 1608 | Mackerel prepared in microwave oven | Makreel bereid in magnetron z toev |
|  |  |  | 1609 | Mackerel in oil tinned | Makreel in olie blik |
|  |  |  | 1610 | Salmon farmed prepared in microwave oven | Zalm kweek- bereid in magnetron z toev |
|  |  |  | 1611 | Trout prepared in microwave oven | Forel bereid in magnetron z toev |
|  |  |  | 1612 | Salmon trout prepared in microwave oven | Forel zalm- bereid in magnetron z toev |
|  |  |  | 1618 | Dab/lemon dab prepared in microwave oven | Schar/tongschar bereid in magnetron z toev |
|  |  |  | 1619 | Sole prepared in microwave oven | Tong bereid in magnetron z toev |
|  |  |  | 1621 | Gurnard prepared in microwave oven | Poon bereid in magnetron z toev |
|  |  |  | 1622 | Perch prepared in microwave oven | Baars rood- bereid in magnetron z toev |
|  |  |  | 1623 | Wolf fish prepared in microwave oven | Zeewolf bereid in magnetron z toev |
|  |  |  | 1624 | Eel prepared in microwave oven | Paling bereid in magnetron z toev |
|  |  |  | 2297 | Tuna raw | Tonijn rauw |
|  |  |  | 2299 | Tilapia raw | Tilapia rauw |
|  |  |  | 2474 | Whitefish fillet batter deep-fried (without fat) | Lekkerbekje bereid (z bereidingsvet) |
|  |  |  | 2765 | Pangasius prepared in microwave oven | Pangasius bereid in magnetron z toev |
| **8** | **2** |  |  | **CRUSTACEANS, MOLLUSCS** |  |
|  |  |  | 111 | Mussels boiled | Mosselen gekookt |
|  |  |  | 348 | Shrimps Dutch peeled boiled | Garnalen Hollandse gekookt |
|  |  |  | 351 | Crab in water tinned | Krab in water blik |
|  |  |  | 352 | Lobster boiled | Kreeft gekookt |
|  |  |  | 354 | Oysters | Oesters |
|  |  |  | 1101 | Snails | Slakken wijngaard- |
|  |  |  | 1632 | Squid prepared in microwave oven | Inktvis pijl- bereid in magnetron z toev |
| **8** | **3** |  |  | **FISH PRODUCTS, FISH IN CRUMBS** |  |
|  |  |  | 814 | Fish fingers fried | Vissticks gebakken |
|  |  |  | 815 | Fish fingers unprepared | Vissticks onbereid |
|  |  |  | 818 | White fish fillet in batter deep-fried | Lekkerbekje gefrituurd |
|  |  |  | 819 | Cod boiled | Kabeljauw gekookt |
|  |  |  | 1097 | Caviar/cod roe | Kaviaar |
|  |  |  | 2474 | Whitefish fillet batter deep-fried (without fat) | Lekkerbekje bereid (z bereidingsvet) |
|  |  |  | 2475 | Salmon pate/-mousse | Zalmpate/-mousse |
|  |  |  |  |  |  |
| **9** |  |  |  | **EGGS AND EGG PRODUCTS** |  |
| **9** | **1** |  |  | **EGG** |  |
|  |  |  | 83 | Eggs chicken raw average | Ei kippen- rauw gem |
|  |  |  | 84 | Eggs chicken boiled average | Ei kippen- gekookt gem |
|  |  |  | 85 | Eggs chicken yolk raw | Eidooier kippen- rauw |
|  |  |  | 86 | Egg yolk chicken boiled | Eidooier kippen- gekookt |
|  |  |  | 358 | Eggs chicken white raw | Eiwit kippenei rauw |
|  |  |  |  |  |  |
| **10** |  |  |  | **FAT** |  |
|  | 0 |  | 313 | Oil soya | Olie soja- |
|  |  |  | 314 | Lard | Vet varkens- uitgesmolten |
|  |  |  | 601 | Oil olive | Olie olijf- |
|  |  |  | 2063 | Margarine 80% fat >24 g saturates | Margarine 80% vet >24 g verz vetz |
|  |  |  | 2066 | Cooking fat liquid 97% fat <17 g sat | Bak- en braadvet vloeib 97% vet <17g verz vetz |
|  |  |  | 2067 | Cooking fat solid 97% fat >17 g sat | Bak- en braadvet vast 97% vet >17 g verz vetz |
|  |  |  | 2068 | Frying fat liquid <24 g sat fatty acids | Frituurvet vloeibaar <24 g verz vetz |
|  |  |  | 2069 | Frying fat solid >24 g sat <10 g trans | Frituurvet vast >24 g verz vetz <10 g transvetz |
|  |  |  | 2525 | Frying fat horeca | Frituurvet horeca |
| **10** | **1** |  |  | **VEGETABLE OILS** |  |
|  |  |  | 308 | Oil peanut | Olie arachide- |
|  |  |  | 312 | Oil corn | Olie mais- |
|  |  |  | 313 | Oil soya | Olie soja- |
|  |  |  | 317 | Oil sunflower seed | Olie zonnebloem- |
|  |  |  | 601 | Oil olive | Olie olijf- |
|  |  |  | 606 | Oil Becel | Olie Becel- |
|  |  |  | 607 | Oil safflower | Olie saffloer- |
|  |  |  | 608 | Oil corn germ | Olie maiskiem- |
|  |  |  | 2472 | Oil wok average | Olie wok- gem |
| **10** | **2** |  |  | **BUTTER** |  |
|  |  |  | 310 | Butter unsalted | Boter ongezouten |
|  |  |  | 879 | Butter salted | Boter gezouten |
|  |  |  | 1530 | Butter product half fat | Boterproduct halfvolle |
|  |  |  | 2295 | Butter product 25% fat | Boterproduct 25% vet |
| **10** | **3** |  |  | **MARGARINES** |  |
|  |  |  | 310 | Butter unsalted | Boter ongezouten |
|  |  |  | 1839 | Low fat margarine product tub Becel Light | Halvarineproduct kuipje Becel Light |
|  |  |  | 1847 | Margarine product tub Becel Dieet | Margarineproduct kuipje Becel Dieet |
|  |  |  | 1956 | Low fat margarine product Becel pro-activ | Halvarineproduct Becel pro-activ |
|  |  |  | 1961 | Low fat margarine product Blue Band Goede Start | Halvarineproduct Blue Band Goede Start |
|  |  |  | 2059 | Low fat margarine 40% fat <17 g sat | Halvarine 40% vet <17 g verz vetz |
|  |  |  | 2060 | Low fat margarine product 35% fat <10 g sat | Halvarineproduct 35% vet <10g verz vetz ongezouten |
|  |  |  | 2061 | Low fat margarine product 20-25% fat <10 g sat | Halvarineproduct 20-25% vet <10 g verz vetz |
|  |  |  | 2062 | Margarine 80% fat 17-24 g saturates | Margarine 80% vet 17-24 g verz vetz |
|  |  |  | 2063 | Margarine 80% fat >24 g saturates | Margarine 80% vet >24 g verz vetz |
|  |  |  | 2064 | Margarine product 70% fat <17 g sat | Margarineproduct 70% vet <17 g verz vetz |
|  |  |  | 2065 | Margarine product 70% fat >17 g sat | Margarineproduct 70% vet >17 g verz vetz |
|  |  |  | 2066 | Cooking fat liquid 97% fat <17 g sat | Bak- en braadvet vloeib 97% vet <17g verz vetz |
|  |  |  | 2067 | Cooking fat solid 97% fat >17 g sat | Bak- en braadvet vast 97% vet >17 g verz vetz |
|  |  |  | 2069 | Frying fat solid >24 g sat <10 g trans | Frituurvet vast >24 g verz vetz <10 g transvetz |
|  |  |  | 2072 | Margarine product 60% fat <17 g sat | Margarineproduct 60% vet <17 g verz vetz ongez |
|  |  |  | 2073 | Cooking fat solid 80% fat >17 g sat | Bak- en braadvet vast 70-80% vet >17 g vvz |
|  |  |  | 2076 | Low fat margarine product Benecol light | Halvarineproduct Benecol light |
|  |  |  | 2077 | Margarine liq 80% fat <17 g saturates | Margarine vloeibaar 80% vet <17 g verz vetz |
|  |  |  | 2294 | Low fat margarine product Twenty four Ultralight | Halvarineproduct Twenty four Ultralight |
|  |  |  | 2295 | Butter product 25% fat | Boterproduct 25% vet |
|  |  |  | 2422 | Low fat margarine product Becel Omega-3 Plus | Halvarineproduct Becel Omega-3 Plus |
|  |  |  | 2423 | Low fat margarine product Blue Band Idee | Halvarineproduct Blue Band Idee |
|  |  |  | 2557 | Margarine 80% fat > 24 g sat unsalted | Margarine 80% vet > 24 g verz vetz ongezouten |
|  |  |  | 2558 | Margarine liq 80% fat < 17g sat unsalted | Margarine vlb 80% vet < 17 g verz vetz ongezouten |
|  |  |  | 2559 | Low fat margarine product AH Bewust light | Halvarineproduct Albert Heijn Bewust light |
|  |  |  | 2562 | Cooking fat liq 97%fat <17g sat unsalted | Bak- en braadvet vlb 97% vet <17g verz vetz ongezouten |
|  |  |  | 2563 | Cooking fat sol 97% fat>17g sat unsalted | Bak- en braadvet vast 97% vet > 17g verz vetz ongezouten |
|  |  |  | 2565 | Margarine 80% fat 17-24g sat unsalted | Margarine 80% vet 17-24g verz vetz ongezouten |
|  |  |  | 2566 | Low fat marg 40% fat <17g sat unsalted | Halvarine 40% vet <17g verz vetz ongezouten |
|  |  |  | 2567 | Low fat margarine product AH chol reductive | Halvarineproduct Albert Heijn chol verlagend |
|  |  |  | 2709 | Low fat margarine product C1000 Goed begin | Halvarineproduct C1000 Goed begin |
|  |  |  | 2711 | Margarine product AlbertHeijn Bewust | Margarineproduct Albert Heijn Bewust |
|  |  |  | 2712 | Margarine product liquid light unsalted | Margarineproduct vloeibaar light ongezouten |
|  |  |  | 2713 | Margarine product liquid light | Margarineproduct vloeibaar light |
|  |  |  | 2849 | Low fat margarine product Becel calorie light | Halvarineproduct Becel calorie light |
|  |  |  | 2850 | Low fat margarine product Becel pro-activ bloeddruk | Halvarineproduct Becel pro.activ bloeddruk |
|  |  |  | 2852 | Low fat margarine product Dieetella Double Activ/Topvit | Halvarineproduct Dieetella Double Activ/TopVit |
|  |  |  | 2878 | Low fat margarine product Gezonde Start | Halvarineproduct Gezonde Start |
|  |  |  | 2934 | Low fat margarine product Becel pro.activ calorie light | Halvarineproduct Becel pro-activ calorie light |
| **10** | **4** |  |  | **DEEP-FRYING FATS** |  |
|  |  |  | 2068 | Frying fat liquid <24 g sat fatty acids | Frituurvet vloeibaar <24 g verz vetz |
|  |  |  | 2069 | Frying fat solid >24 g sat <10 g trans | Frituurvet vast >24 g verz vetz <10 g transvetz |
|  |  |  | 2525 | Frying fat horeca | Frituurvet horeca |
| **10** | **5** |  |  | **MARINE OIL** |  |
| **10** | **6** |  |  | **OTHER ANIMAL FAT** |  |
|  |  |  | 314 | Lard | Vet varkens- uitgesmolten |
|  |  |  |  |  |  |
| **11** |  |  |  | **SUGAR AND CONFECTIONERY** |  |
|  | 0 |  | 440 | Ginger stem in syrup tinned | Gember op siroop blik/glas |
|  |  |  | 832 | Ginger root fresh | Gemberwortel |
| **11** | **1** |  |  | **SUGAR, HONEY, JAM** |  |
|  |  |  | 374 | Sugar brown Basterdsuiker | Suiker basterd- bruine |
|  |  |  | 375 | Sugar white Basterdsuiker | Suiker basterd- witte |
|  |  |  | 377 | Sugar granulated | Suiker kristal- |
|  |  |  | 443 | Honey | Honing |
|  |  |  | 445 | Jam | Jam |
|  |  |  | 457 | Jam rose hip | Jam rozenbottel- |
|  |  |  | 484 | Jam reduced sugar | Jam halfzoet |
|  |  |  | 807 | Jam without sugar | Jam z suiker |
|  |  |  | 1065 | Preparation glucose powder Dextro-M | Glucosepoeder Dextro-M |
|  |  |  | 2343 | Jelly | Pudding gelatine- |
|  |  |  | 2368 | Milk drink pow with fruit flavour Nesquik plus | Poeder voor melkdrank vr smaak Nesquik plus |
| **11** | **2** |  |  | **CHOCOLATE, CANDY BARS, PASTE, CONFETTI/FLAKES** | |
|  |  |  | 430 | Cocoa powder | Cacaopoeder |
|  |  |  | 431 | Chocolate milk | Chocolade melk- |
|  |  |  | 432 | Chocolate plain | Chocolade puur |
|  |  |  | 433 | Chocolate flakes milk | Vlokken chocolade- melk |
|  |  |  | 434 | Chocolate butter | Boter chocolade- |
|  |  |  | 435 | Chocolate flakes plain | Vlokken chocolade- puur |
|  |  |  | 436 | Spread chocolate hazelnut | Pasta chocolade- hazelnoot |
|  |  |  | 444 | Spread chocolade plain | Pasta chocolade- puur |
|  |  |  | 487 | Candybar Mars | Candybar Mars |
|  |  |  | 524 | M&M's chocolate | M&M's chocolade |
|  |  |  | 525 | Candybar Milky Way | Candybar Milky Way |
|  |  |  | 526 | Candybar Bounty | Candybar Bounty |
|  |  |  | 528 | Candybar Snickers | Candybar Snickers |
|  |  |  | 535 | Cocoa powder sweetened Benco | Cacaopoeder gezoet Benco |
|  |  |  | 570 | Candybar Nuts | Candybar Nuts |
|  |  |  | 595 | Cocoa product powder Ovomaltine | Cacaoproduct poeder Ovomaltine |
|  |  |  | 621 | M&M's chocolate with peanuts | M&M's chocolade m pinda's |
|  |  |  | 717 | Chocolate bar milk with nuts | Chocolade melk- m noten |
|  |  |  | 727 | Chocolate liqueurs | Bonbon m likeur |
|  |  |  | 845 | Candybar Twix | Candybar Twix |
|  |  |  | 929 | Chocolate bar milk without sugar | Chocolade melk- z suiker |
|  |  |  | 1311 | Chocolate confetti averaged | Hagelslag chocolade- gem |
|  |  |  | 1450 | Chocolate plain without sugar | Chocolade puur z suiker |
|  |  |  | 1508 | Chocolates filled/Belguim chocolate | Bonbon |
|  |  |  | 1509 | Muesli bar with chocolate | Mueslireep m chocolade |
|  |  |  | 1962 | Chocolate confetti milk | Hagelslag chocolade- melk |
|  |  |  | 1963 | Chocolate confetti plain | Hagelslag chocolade- puur |
|  |  |  | 1964 | Spread chocolate milk | Pasta chocolade- melk |
|  |  |  | 2234 | Cereal bar Hero B'tween | Graanreep Hero B'tween |
|  |  |  | 2235 | Cereal bar Hero B'tween free | Graanreep Hero B'tween free |
|  |  |  | 2237 | Cereal bar Special K Kellogg's | Graanreep Special K Kellogg's |
|  |  |  | 2266 | Chocolate white | Chocolade witte |
|  |  |  | 2372 | Raisins coated with milkchocolate | Rozijnen omhuld m chocolade melk- |
|  |  |  | 2373 | After eight chocolate mints | After eight |
|  |  |  | 2374 | Chocolate milk with raisins | Chocolade melk- m rozijnen |
|  |  |  | 2375 | Chocolate plain with nuts | Chocolade puur m noten |
|  |  |  | 2376 | Chocolate milk with puffed rice | Chocolade melk- m gepofte rijst |
|  |  |  | 2415 | Cocoa product sweetened Nesquik Hot Choc | Cacaoproduct poeder gezoet Nestle Hot Chocolate |
|  |  |  | 2424 | Chocolate confetti white | Hagelslag chocolade wit- |
|  |  |  | 2530 | Maltesers | Maltesers |
|  |  |  | 2531 | Chocolate flakes average | Vlokken chocolade- gem |
|  |  |  | 2535 | Cocoa powder sweetened Nesquik plus | Cacaopoeder gezoet Nesquik plus |
|  |  |  | 2595 | Candybar KitKat | Candybar KitKat |
|  |  |  | 2656 | Chocolate confetti mix white and plain | Hagelslag chocolade mix wit en puur |
|  |  |  | 2657 | Spread duo with chocolate | Pasta duo- m chocolade |
|  |  |  | 2658 | Spread duo without chocolate | Pasta duo- z chocolade |
|  |  |  | 2666 | Spread chocolate Duo Penotti hazelnut | Pasta Duo Penotti chocolade- hazelnoot |
|  |  |  | 2829 | Chocolate bar filled Kinder | Chocoladereep gevuld Kinder |
|  |  |  | 2870 | Candybar Lion | Candybar Lion |
|  |  |  | 2930 | Chocolate spread white | Pasta chocolade- wit |
| **11** | **3** |  |  | **CONFECTIONERY NON CHOCOLATE** |  |
|  |  |  | 377 | Sugar granulated | Suiker kristal- |
|  |  |  | 442 | Coloured confetti fruit-flavoured | Hagelslag vruchten- |
|  |  |  | 446 | Chewing gum | Kauwgom |
|  |  |  | 447 | Chewing gum without sugar | Kauwgom z suiker |
|  |  |  | 449 | Coconut bread sweetened sliced | Kokosbrood |
|  |  |  | 450 | Boiled sweets | Zuurtjes |
|  |  |  | 453 | Peppermint | Pepermunt |
|  |  |  | 461 | Toffees | Toffee |
|  |  |  | 482 | Sweets marshmallow type Spekkie | Spekkie |
|  |  |  | 520 | Liquorice Dutch type salted | Drop zoute |
|  |  |  | 521 | Liquorice Dutch type double salted | Drop dubbelzoute |
|  |  |  | 522 | Liquorice Dutch type sweet | Drop zoete |
|  |  |  | 523 | Liquorice Stophoest | Stophoest |
|  |  |  | 750 | Marshmallows | Marshmellows |
|  |  |  | 751 | Liquorice all sorts | Drop Engelse |
|  |  |  | 752 | Wine gums | Winegums |
|  |  |  | 866 | Sweets fruit pectinbased | Snoep op pectinebasis vruchten- |
|  |  |  | 1256 | Marzipan | Marsepein |
|  |  |  | 1364 | Turkish Delight | Turks fruit |
|  |  |  | 1507 | Fondant cream | Borstplaat room- |
|  |  |  | 2234 | Cereal bar Hero B'tween | Graanreep Hero B'tween |
|  |  |  | 2237 | Cereal bar Special K Kellogg's | Graanreep Special K Kellogg's |
|  |  |  | 2239 | Muesli bar | Mueslireep |
|  |  |  | 2292 | Boiled sweets sugar free | Zuurtjes suikervrij |
|  |  |  | 2380 | Toffee with chocolate | Toffee m chocolade |
|  |  |  | 2383 | Nougat with chocolate | Nougat m chocolade |
|  |  |  | 2384 | Liquorice with peppermint | Drop m pepermunt |
|  |  |  | 2385 | Liquorice with sal ammoniac powder | Salmiakkogel |
|  |  |  | 2386 | Nougat | Nougat |
|  |  |  | 2387 | Popcorn sweet puffed | Popcorn gepoft zoet |
|  |  |  | 2489 | Spread spiced biscuit flavoured | Pasta speculoos- |
|  |  |  | 2532 | Liquorice sugarfree | Drop suikervrij |
|  |  |  | 2533 | Wine gum with liquorice | Winegum m drop |
|  |  |  | 2659 | Sweets jelly/gums/foam | Snoep schuim-/gum- |
|  |  |  | 2660 | Wine gums with foam layer | Winegum m schuimlaag |
|  |  |  | 2691 | Sweet fruity chew | Kauwbonbon fruitsmaak |
| **11** | **4** |  |  | **SYRUP** |  |
|  |  |  | 376 | Syrup golden | Stroop huishoud- |
|  |  |  | 381 | Syrup sugar | Stroop suiker- |
|  |  |  | 427 | Syrup apple | Stroop appel- |
|  |  |  | 463 | Fruit drink concentrate undiluted | Siroop vruchtenlimonade- |
|  |  |  | 497 | Fruit drink concentrate fruitmix | Siroop vruchtenmix |
|  |  |  | 498 | Fruit drink concentrate Roosvicee Ferro | Siroop Roosvicee Ferro |
|  |  |  | 500 | Fruit drink concentrate Roosvicee Laxo | Siroop Roosvicee Laxo |
|  |  |  | 501 | Fruit drink concentrate Roosvicee Stop | Siroop Roosvicee Stop |
|  |  |  | 738 | Fruit drink concentrate Roosvicee Dieet | Siroop Roosvicee Dieet |
|  |  |  | 1467 | Syrup from tinned fruit | Vocht van vruchtenconserven blik/glas |
|  |  |  | 1655 | Fruit juice concentrated | Diksap geconcentreerd |
|  |  |  | 1807 | Fruit drink concentrate other flavours | Siroop vruchtenlimonade- overige smaken Spontin |
|  |  |  | 1810 | Fruit drink concentrate Karvan Cevitam | Siroop vruchtenlimonade- Karvan Cevitam |
|  |  |  | 1880 | Fruit drink concentrate Roosvicee Calcium | Siroop Roosvicee Calcium |
|  |  |  | 1881 | Fruit drink concentrate Roosvicee Multivit | Siroop Roosvicee Multivit bosvr/perzik |
|  |  |  | 1882 | Fruit drink concentrate low sugar Lessini | Siroop Roosvicee Lessini light |
|  |  |  | 2287 | Fruit drink concentrate with sugar and sweeteners | Siroop vruchtenlimonade- m suiker en zoetstof |
|  |  |  | 2288 | Fruit drink concentrate with sugar and sweetener with vitC | Siroop vruchtenlim m suiker en zoetst m vit C |
|  |  |  | 2289 | Fruit drink concentrate light | Siroop vruchtenlimonade- light |
|  |  |  | 2291 | Fruit juice concentrated with added vit C | Diksap m vit C geconcentreerd |
|  |  |  | 2388 | Fruit drink concentrate with 10-15 mg vit C | Siroop vruchtenlimonade- m 10-15 mg vit C |
|  |  |  | 2389 | Fruit drink concentrate can Albert Heijn | Siroop vruchtenlimonade- blik Albert Heijn |
|  |  |  | 2390 | Fruit drink concentrate with 15-20 mg vit C | Siroop vruchtenlimonade- m 15-20 mg vit C |
|  |  |  | 2534 | Fruitdrink concentrate sugar and sweetener 5-10 g CHO | Siroop vruchtenlim m suiker en zoetst 5-10g KH |
|  |  |  | 2680 | Fruit drink concentrate with 40 mg vit C | Siroop vruchtenlimonade- m 40 mg vit C |
|  |  |  | 2831 | Fruit drink concentrate with sugar and sweetener 10-15 g CHO | Siroop vruchtenlim m suiker en zoetst 10-15g KH |
| **11** | **5** |  |  | **ICE CREAM, WATER ICE** |  |
| **11** | **5** | **1** |  | **ICE CREAM** |  |
|  |  |  | 303 | Ice cream dairy cream based | IJs room/vanille- |
|  |  |  | 485 | Ice cream dairy cornet | IJs room/vanille cornet |
|  |  |  | 1002 | Ice cream dairy Delight Line without ad sugar | IJs gst Delight Line Ton Puts |
|  |  |  | 2251 | Ice cream dairy with chocolate coating | IJs room/vanille m chocoladecoating |
|  |  |  | 2252 | Ice cream dairy with fruitcoating | IJs room/vanille m fruitcoating |
|  |  |  | 2416 | Ice cream stracciatella- | IJs stracciatella- |
| **11** | **5** | **2** |  | **SORBET** |  |
|  |  |  | 1474 | Ice lolly/ Sorbet | IJs water- |
| **11** | **5** | **3** |  | **WATER ICE** |  |
|  |  |  | 1474 | Ice lolly/ Sorbet | IJs water- |
|  |  |  | 2250 | Ice lolly Festini | IJs Festini |
|  |  |  |  |  |  |
| **12** |  |  |  | **CAKES** |  |
| **12** | **1** |  |  | **CAKES, PIES, PASTRIES, PUDDINGS (NON-MILK BASED)** | |
|  |  |  | 232 | Danish pastry | Broodje koffie- |
|  |  |  | 240 | Cake Dutch spiced Ontbijtkoek | Koek ontbijt- |
|  |  |  | 250 | Almond filled pastry | Broodje amandel- |
|  |  |  | 251 | Apple pie Dutch with shortbread without butter | Taart appel- van zandtaartdeeg z roomboter |
|  |  |  | 253 | Cake without butter | Cake z roomboter |
|  |  |  | 254 | Cake sponge Dutch Eierkoek | Koek eier- |
|  |  |  | 255 | Gateau with whipped cream | Taart slagroom- |
|  |  |  | 256 | Gateau with butter-cream filling | Taart crème au beurre- |
|  |  |  | 257 | Almond paste filled tarts average | Koek gevulde gem |
|  |  |  | 467 | Pancake | Pannenkoek |
|  |  |  | 468 | Cream slice Dutch Tompouce | Tompouce |
|  |  |  | 474 | Doughnut Dutch style | Oliebol |
|  |  |  | 486 | Flan with fruit filling | Vlaai vruchten- |
|  |  |  | 489 | Flan filled with rice pudding | Vlaai rijste- |
|  |  |  | 789 | Cake butter Dutch Boterkoek | Koek boter- |
|  |  |  | 833 | Apple strudel | Appelcarre |
|  |  |  | 835 | Cheesecake made with fromage frais | Taart kwark- |
|  |  |  | 837 | Biscuit oatmeal | Koek haver- |
|  |  |  | 854 | Gateau fatless sponge with fruit and whipped cream | Taart vruchten- van biscuitdeeg m slagroom |
|  |  |  | 855 | Biscuit spiced Speculaas with almnd paste | Speculaas gevulde |
|  |  |  | 878 | Croissants | Croissants uit blik afgebakken |
|  |  |  | 925 | Cake Dutch spiced Ontbijtkoek wholemeal | Koek ontbijt- volkoren |
|  |  |  | 1365 | Baklava nut-honey cake | Baklava |
|  |  |  | 1460 | Cake Dutch spiced Ontbijtkoek with ginger | Koek ontbijt- gember |
|  |  |  | 1470 | Berliner pastry | Berliner bol |
|  |  |  | 1473 | Doughnuts plain | Donuts ongevuld |
|  |  |  | 1475 | Eclair with whipped cream filling | Soes slagroom- |
|  |  |  | 1476 | Meringue with butter-cream | Taart schuim- m creme au beurre |
|  |  |  | 1477 | Biscuits sugar free | Koekje suikervrij |
|  |  |  | 1478 | Flan fruit and crumble topping | Vlaai kruimel- m vruchten |
|  |  |  | 1479 | Cheesecake made with cream cheese | Taart Mon Chou- |
|  |  |  | 1915 | Eclair without filling | Soes ongevuld |
|  |  |  | 1945 | Bun with vanilla custard | Broodje pudding- |
|  |  |  | 1969 | Cake made with butter | Cake m roomboter |
|  |  |  | 2010 | Sponge cake with fruit | Taart vruchten- van cakedeeg |
|  |  |  | 2190 | Brownies with nuts | Brownies met noten |
|  |  |  | 2222 | Cake Dutch spiced Ontbijtkoek Happers | Ontbijtkoekreep Happers |
|  |  |  | 2224 | Cake Dutch spiced Kapitein Koek | Ontbijtkoekreep Kapitein Koek |
|  |  |  | 2232 | Biscuit fruit | Biscuit fruit- |
|  |  |  | 2233 | Cake Dutch spiced Ontbijtkoek with raisins | Koek ontbijt- m rozijnen |
|  |  |  | 2234 | Cereal bar Hero B'tween | Graanreep Hero B'tween |
|  |  |  | 2329 | Cake Dutch spiced Ontbijtkoek less sugar | Koek ontbijt- minder suiker |
|  |  |  | 2391 | Cake marble- | Cake marmer- |
|  |  |  | 2392 | Cake apple- | Cake appel- |
|  |  |  | 2393 | Cake raisins- | Cake rozijnen- |
|  |  |  | 2394 | Eclair filled with banana and whipped cream | Soes bananen- |
|  |  |  | 2395 | Chocolate éclair | Moorkop |
|  |  |  | 2396 | Chocolate pastry with whipped cream | Gebak chocolade- m slagroom |
|  |  |  | 2397 | Cake Dutch spiced Ontbijtkoek with nuts | Koek ontbijt- m noten |
|  |  |  | 2398 | Cake Dutch spiced Ontbijtkoek with rockcandy | Koek ontbijt- m kandij |
|  |  |  | 2399 | Spiced cake Indonesian- Spekkoek | Spekkoek |
|  |  |  | 2400 | Croissant chocolate- | Croissant chocolade- |
|  |  |  | 2401 | Tarts filled with jam | Koek met gelei/appelvulling |
|  |  |  | 2403 | Cup cake iced | Koek Glace- |
|  |  |  | 2404 | Cake Dutch spiced with sunflower seeds | Ontbijtkoekreep zonnebloempitten Bammetje |
|  |  |  | 2405 | Chocolate chip cookie | Koekje chocolate chip cookie |
|  |  |  | 2428 | Wafer with milk and hazelnuts Knoppers | Wafel Knoppers |
|  |  |  | 2429 | Cake with nuts | Cake m noten |
|  |  |  | 2430 | Fritter banana | Beignet banaan- |
|  |  |  | 2431 | Gateau fatless sponge with marzipan | Taart marsepein- |
|  |  |  | 2432 | Cake with nuts | Taart noten- |
|  |  |  | 2560 | Apple turnover | Appelflap |
|  |  |  | 2568 | Flan with custard and crumble topping | Vlaai kruimel- m pudding |
|  |  |  | 2569 | Cake Dutch Ontbijtkoek with nuts and fruit | Koek ontbijt- m noten en vruchten |
|  |  |  | 2570 | Flan with custard and fruit | Vlaai pudding- m vruchten |
|  |  |  | 2571 | Cake with macaroons | Cake bitterkoekjes- |
|  |  |  | 2577 | Cake made with butter and apple | Cake roomboter- m appel |
|  |  |  | 2578 | Pancake prepared with sunflower oil | Pannenkoek bereid m zonnebloemolie |
|  |  |  | 2579 | Pancake prepared with olive oil | Pannenkoek bereid m olijfolie |
|  |  |  | 2581 | Pancake wholemeal prepared with marg liq 2077 | Pannenkoek volkoren bereid m marg vlb 2077 |
|  |  |  | 2582 | Pancake wholemeal | Pannenkoek volkoren |
|  |  |  | 2608 | Biscuit childrens Smoeltje fruit | Koekje kinder- Smoeltje fruit |
|  |  |  | 2622 | Appel pie Dutch with shortbread with butter | Taart appel- van zandtaartdeeg m roomboter |
|  |  |  | 2661 | Waffle Luikse | Wafel Luikse- |
|  |  |  | 2662 | Waffle soft-/sugar-/flash- | Wafel zachte-/suiker-/flash- |
|  |  |  | 2698 | Cake chocolate made without butter | Cake chocolade- z roomboter |
|  |  |  | 2721 | Doughnuts iced | Donuts m glazuur |
|  |  |  | 2722 | Waffle Luikse with chocolate | Wafel dikke Luikse m chocolade |
|  |  |  | 2761 | Almond paste filled tarts with butter | Koek gevulde m roomboter |
|  |  |  | 2776 | Flan apple and crumble topping | Vlaai appelkruimel- |
|  |  |  | 2777 | Flan hard shell with custard cream | Vlaai wener bodem m bakkersroom |
|  |  |  | 2778 | Flan sponge with custard cream | Vlaai zachte bodem m bakkersroom |
|  |  |  | 2801 | Croissant prepared with butter | Croissant roomboter- |
|  |  |  | 2802 | Croissant prepared without butter | Croissant z roomboter |
|  |  |  | 2818 | Croissant average | Croissant gem |
|  |  |  | 2832 | Cake chocolate made with butter | Cake chocolade- m roomboter |
|  |  |  | 2862 | Pancake with apple and raisin | Pannenkoek met appel en rozijnen |
|  |  |  | 2863 | Pie apple-nuts | Taart appel-noten |
|  |  |  | 2868 | Pancake prepared with marg liquid 2077 | Pannenkoek bereid m maragrine vloeib 2077 |
|  |  |  | 2869 | Fritter apple | Beignet appel- |
| **12** | **2** |  |  | **DRY CAKES, BISCUITS** |  |
|  |  |  | 234 | Biscuit fortified Liga Tweede Stap | Voedingsbiscuit Liga Tweede Stap |
|  |  |  | 235 | Biscuit fort Liga Derde Stap Wholemeal | Voedingsbiscuit Liga Derde Stap Meergranen |
|  |  |  | 240 | Cake Dutch spiced Ontbijtkoek | Koek ontbijt- |
|  |  |  | 252 | Biscuit sweet | Biscuit |
|  |  |  | 254 | Cake sponge Dutch Eierkoek | Koek eier- |
|  |  |  | 258 | Biscuits averaged | Koekje gem |
|  |  |  | 259 | Macaroons | Kokosmakronen |
|  |  |  | 260 | Biscuit sponge fingers | Lange vingers |
|  |  |  | 261 | Biscuit spiced Speculaas | Speculaas gem |
|  |  |  | 262 | Biscuit Dutch shortbread spritsstukken | Spritsstukken |
|  |  |  | 263 | Biscuit brown/wholemeal | Biscuit tarwe-/volkoren- |
|  |  |  | 480 | Biscuit chocolate coated Chocoprins | Prince Chocoprince |
|  |  |  | 481 | Biscuit shortbread Bastogne | Koek Bastogne |
|  |  |  | 633 | Meringue cake Bokkenpootje | Bokkenpootje |
|  |  |  | 634 | Coconut flavoured cookies | Koek kokos- |
|  |  |  | 635 | Biscuits Dutch krakeling | Krakeling |
|  |  |  | 636 | Biscuit muesli | Koek muesli- |
|  |  |  | 713 | Waffle syrup average | Wafel stroop- gem |
|  |  |  | 790 | Almond paste with egg | Amandelspijs m ei |
|  |  |  | 836 | Shortbread | Zandtaartjes |
|  |  |  | 837 | Biscuit oatmeal | Koek haver- |
|  |  |  | 855 | Biscuit spiced Speculaas with almnd paste | Speculaas gevulde |
|  |  |  | 873 | Biscuit fortifd with currants Liga Evergreen | Voedingsbiscuit Liga Evergreen krenten |
|  |  |  | 1318 | Biscuit fortified Liga Eerste Stap | Voedingsbiscuit Liga Eerste Stap |
|  |  |  | 1321 | Biscuit fortified Liga FruitKick | Voedingsbiscuit Liga FruitKick |
|  |  |  | 1353 | Biscuit gluten-free Glutafin | Biscuit glutenvrij Glutafin |
|  |  |  | 1356 | Biscuit fortified Liga Evergr assortment | Voedingsbiscuit Liga Evergreen ov smaken |
|  |  |  | 1471 | Biscuit chocolate | Biscuit chocolade- |
|  |  |  | 1476 | Meringue with butter-cream | Taart schuim- m crème au beurre |
|  |  |  | 1477 | Biscuits sugar free | Koekje suikervrij |
|  |  |  | 1480 | Biscuit Jaffa cakes/Cake PiM's | Koekje PiM's sinaasappel |
|  |  |  | 1767 | Biscuits children's Bambix beestenkoekje | Koekjes beesten- Bambix |
|  |  |  | 1965 | Biscuit fortified Liga Milkbreak | Voedingsbiscuit Liga Milkbreak |
|  |  |  | 1972 | Biscuits assorted with butter | Koekje roomboter- gem |
|  |  |  | 2162 | Biscuit Café noir | Café noir |
|  |  |  | 2225 | Biscuit fortified Fruitkick Extra | Voedingsbiscuit Liga Fruitkick Extra |
|  |  |  | 2226 | Biscuit fortified Liga Continue vit/min | Voedingsbiscuit Liga Continue vit en min |
|  |  |  | 2227 | Biscuit filled Prince | Prince gevulde biscuit |
|  |  |  | 2228 | Biscuit with chocolate layer Scholiertje | Koekje Scholiertje |
|  |  |  | 2232 | Biscuit fruit | Biscuit fruit- |
|  |  |  | 2233 | Cake Dutch spiced Ontbijtkoek with raisin | Koek ontbijt- m rozijnen |
|  |  |  | 2234 | Cereal bar Hero B'tween | Graanreep Hero B'tween |
|  |  |  | 2401 | Tarts filled with jam | Koek met gelei/appelvulling |
|  |  |  | 2403 | Cup cake iced | Koek Glace- |
|  |  |  | 2405 | Chocolate chip cookie | Koekje chocolate chip cookie |
|  |  |  | 2406 | Biscuit children's average | Koekje kinder- gem |
|  |  |  | 2407 | Biscuit fortified Provita milkbiscuit | Voedingsbiscuit melkbiscuit Provita |
|  |  |  | 2408 | Biscuit fortified Bridge fruit and grain | Voedingsbiscuit fruit en granen Bridge |
|  |  |  | 2409 | Biscuit Dutch shortbread with chocolate | Spritsstukken m chocolade |
|  |  |  | 2410 | Teacakes chocolate coated marshmallow | Schuimzoenen |
|  |  |  | 2412 | Gingersnap | Kletskop |
|  |  |  | 2413 | Cake wrapped in marzipan and chocolate | Mergpijpje |
|  |  |  | 2414 | Biscuit fortified Bridge milkbiscuit | Voedingsbiscuit melkbiscuit Bridge |
|  |  |  | 2417 | Cake Dutch with icing and cream 'Oranjekoek' | Koek oranje- |
|  |  |  | 2425 | Wafer galette | Wafel galette- |
|  |  |  | 2427 | Spice biscuit sprinkles Bolletje | Schuddebuikjes Bolletje |
|  |  |  | 2428 | Wafer with milk and hazelnuts Knoppers | Wafel Knoppers |
|  |  |  | 2556 | Biscuit fortified LU Time Out | Voedingsbiscuit LU Time Out |
|  |  |  | 2572 | Biscuit fortified milk Perfekt/Plus | Voedingsbiscuit melkbiscuit Perfekt/Plus div smaken |
|  |  |  | 2583 | Biscuit peanut | Koek pinda- |
|  |  |  | 2607 | Biscuit with nuts and chocolate | Koekje m noten en chocolade |
|  |  |  | 2608 | Biscuit children’s Smoeltje fruit | Koekje kinder- Smoeltje fruit |
|  |  |  | 2609 | Biscuits with nuts | Koekje m noten |
|  |  |  | 2661 | Waffle Luikse | Wafel Luikse- |
|  |  |  | 2687 | Biscuit spiced kruidnoten with plain choc | Kruidnoten m chocolade puur |
|  |  |  | 2688 | Biscuit spiced kruidnoten with milk choc | Kruidnoten m chocolade melk- |
|  |  |  | 2696 | Biscuit Dutch Amaretti Bitterkoekjes | Koekje bitter- |
|  |  |  | 2699 | Biscuit Dutch Frou frou | Frou frou |
|  |  |  | 2700 | Biscuit with dried fruit and yoghurt Yofruit | Biscuit Sultana Yofruit |
|  |  |  | 2701 | Biscuit spiced kruidnoten with white choc | Kruidnoten m chocolade witte |
|  |  |  | 2714 | Biscuit fortified Evergreen crunchy | Voedingsbiscuit Evergreen crunchy |
|  |  |  | 2715 | Meringue | Schuimbaton |
|  |  |  | 2719 | Biscuit Bridge ommetjes | Koekjes granen- Bridge ommetjes |
|  |  |  | 2763 | Biscuit spiced Speculaas with butter | Speculaas m roomboter |
|  |  |  | 2764 | Biscuit spiced Speculaas without butter | Speculaas z roomboter |
|  |  |  | 2775 | Biscuit digestive with chocolate | Biscuit tarwe- m chocolade |
|  |  |  | 2810 | Waffle syrup without butter | Wafel stroop- z roomboter |
|  |  |  | 2820 | Waffle syrup with butter | Wafel stroop- m roomboter |
|  |  |  | 2864 | Biscuit syrup with chocolate | Koek stroop- met chocola |
|  |  |  | 2933 | Sponge cake wholemeal | Koek eier- volkoren/meergranen |
|  |  |  |  |  |  |
| **13** |  |  |  | **NON ALCOHOLIC BEVERAGES** |  |
|  | 0 |  | 396 | Juice grape | Sap druiven- |
|  |  |  | 400 | Soft drink without caffeine | Frisdrank |
|  |  |  | 1519 | Beer alcohol free <0,1 vol% | Bier alcoholvrij <0,1 vol% alcohol |
|  |  |  | 2290 | Coconut milk | Kokosmelk |
|  |  |  | 2433 | Rice drink Rice Dream Ca+ | Rijstedrank Rice Dream Ca+ |
| **13** | **1** |  |  | **FRUIT AND VEGETABLE JUICES** |  |
|  |  |  | 383 | Juice apple | Sap appel- |
|  |  |  | 385 | Fruit juice drink redcurrant | Vruchtendrank rode bessen |
|  |  |  | 387 | Fruit juice drink blackcurrant | Vruchtendrank zwarte bessen |
|  |  |  | 388 | Juice redcurrant | Sap bessen- |
|  |  |  | 396 | Juice grape | Sap druiven- |
|  |  |  | 399 | Fruit juice drink raspberry | Vruchtendrank frambozen |
|  |  |  | 400 | Soft drink without caffeine | Frisdrank |
|  |  |  | 410 | Juice orange pasteurized | Sap sinaasappel- gepasteuriseerd |
|  |  |  | 413 | Tomato juice | Sap tomaten- |
|  |  |  | 417 | Juice drink | Limonade vruchten- |
|  |  |  | 664 | Juice grapefruit | Sap grapefruit- |
|  |  |  | 861 | Juice beetroot | Sap bieten- |
|  |  |  | 923 | Juice pear | Sap peren- |
|  |  |  | 1127 | Juice lemon fresh | Sap citroen- vers |
|  |  |  | 1294 | Whey drink Taksi with sugar | Weidrank Taksi m suiker |
|  |  |  | 1462 | Juice pineapple | Sap ananas- |
|  |  |  | 1463 | Fruit juice dk minimal 2 fruits | Vruchtendrank 2 of meer vruchten |
|  |  |  | 1521 | Juice drink light | Limonade vruchten- light |
|  |  |  | 1522 | Soft drink light without caffeine | Frisdrank light z cafeine |
|  |  |  | 1878 | Fruit juice drink Roosvicee Multivit | Vruchtendrank Roosvicee Multivit |
|  |  |  | 1932 | Juice orange with pulp | Sap sinaasappel- m vruchtvlees |
|  |  |  | 1933 | Juice tomato/vegetable Appelsientje | Sap tomatengroenten- Appelsientje Tomaat-Groente |
|  |  |  | 1934 | Juice tomato Appelsientje Zontomaat | Sap tomaten- Appelsientje Zontomaat |
|  |  |  | 1998 | Drink fruit Stimulance multifibre | Drink vruchten- Stimulance multivezel |
|  |  |  | 2079 | Breakfast drink HeroFruitontbijt p 100 ml | Drinkontbijt Hero FruitOntbijt p 100 ml |
|  |  |  | 2134 | Juice drink Dubbelfrisss | Limonade vruchten- Dubbelfrisss |
|  |  |  | 2135 | Juice drink Vruchtenfris/Tintelfruit | Limonade vruchten- Vruchtenfris/Tintelfruit |
|  |  |  | 2136 | Lemonade squash Dubbelfrisss light | Limonade vruchten- Dubbelfrisss light |
|  |  |  | 2137 | Juice drink Vruchtenfris/Tintelfruit light | Limonade vruchten- Vruchtenfris/Tintelfruit light |
|  |  |  | 2138 | Juice drink Spa&Fruit still | Limonade vruchten- Spa&Fruit koolzuurvrij |
|  |  |  | 2139 | Juice drink Spa&Fruit light still | Limonade vruchten- Spa&Fruit light koolzuurvrij |
|  |  |  | 2140 | Lemonade squash Spa&Fruit Vitamins | Limonade vruchten- Spa&Fruit Vitamine |
|  |  |  | 2141 | Mineral water with sweetener | Bronwater m zoetstof |
|  |  |  | 2144 | Juice apple with vitamin C | Sap appel- m vit C |
|  |  |  | 2145 | Juice fruit Appelsientje Vitamientje orange fruit | Sap vruchten- Appelsientje Vitamientje oranje vr |
|  |  |  | 2146 | Fruit drink Appelsientje Vitamientje forest fruit | Vruchtendrank Appelsientje Vitamientje bosvruchten |
|  |  |  | 2150 | Juice fruit Coolbest Vitaday tropical | Sap vruchten- Coolbest Vitaday tropical |
|  |  |  | 2151 | Juice fruit Coolbest Vitaday original | Sap vruchten- Coolbest Vitaday original |
|  |  |  | 2152 | Fruit juice dk Coolbest light orange | Vruchtendrank Coolbest light premium orange |
|  |  |  | 2153 | Fruit drink Fruit2day | Fruitdrank Fruit2day |
|  |  |  | 2154 | Juice fruit multi with vitamins SuperdeBoer | Sap vruchten- multivr sap m vit Super de Boer |
|  |  |  | 2220 | Whey drink Taksi with sugar and sweetener | Weidrank Taksi m suiker en zoetstof |
|  |  |  | 2328 | Fruit juice drink with dairy Wicky peach | Vruchtendrank m zuivel Wicky fruitzacht perzik |
|  |  |  | 2434 | Juice drink Wicky | Limonade vruchten- Wicky |
|  |  |  | 2435 | Soft drink with sugar and sweetener 2-<5 g KH | Frisdrank m suiker en zoetstof 2-<5 g KH |
|  |  |  | 2436 | Juice drink with sugar and sw | Limonade vruchten- m suiker en zoetstof |
|  |  |  | 2437 | Juice drink multivitamin Caprisonne | Limonade vruchten- multivitamine Caprisonne |
|  |  |  | 2481 | Fruit juice dk Roosvicee Spongebob | Vruchtendrank Roosvicee Spongebob/Shrek |
|  |  |  | 2483 | Fruit juice drink ACE average | Vruchtendrank ACE gem |
|  |  |  | 2486 | Fruit drink concentrate Karvan Cevitam diluted av | Siroop vrlimonade- Karvan Cevitam bereid gem |
|  |  |  | 2490 | Fruit juice drink 12fruit nectar light | Vruchtendrank multivit 12vr nectar light |
|  |  |  | 2491 | Fruit juicedrink with sweetener 5-<8g CHO | Vruchtendrank m zoetstof 5-<8g KH |
|  |  |  | 2492 | Juice drink Wicky light | Limonade vruchten- Wicky light |
|  |  |  | 2506 | Juice vegetable/fruit Knorr Vie average | Sap groente-/vruchten gem Knorr Vie |
|  |  |  | 2507 | Juice multifruit | Sap multivruchten- |
|  |  |  | 2508 | Fruit juice drink ACE Aldi/Kruidvat | Vruchtendrank ACE Aldi/Kruidvat |
|  |  |  | 2509 | Juice drink Sisi Fruitmania Smooth peach | Limonade vruchten- Sisi Fruitmania Smooth peach |
|  |  |  | 2510 | Juice drink Sisi Fruitmania Smooth berry | Limonade vruchten- Sisi Fruitmania Smooth berry |
|  |  |  | 2511 | Fruit juice Solevita multivit 12-fruits | Sap vruchten- Solevita multivitamine 12-vruchten |
|  |  |  | 2512 | Fruit juice drink Fruity King tropical | Vruchtendrank Fruity King tropical fruit |
|  |  |  | 2514 | Fruit juice concentrated prepared | Diksap bereid |
|  |  |  | 2515 | Fruit juice dk Surango multivit light | Vruchtendrank multivit nectar light Surango |
|  |  |  | 2564 | Juice fruit Hero Fruit&Co | Sap vruchten- Hero Fruit&Co |
|  |  |  | 2634 | Fruit juice dk minimal 2 fruits with vit C | Vruchtendrank 2 of meer vruchten m vit C |
|  |  |  | 2635 | Fruit juice drink dairy Wicky fruitzacht red fruit | Vruchtendrank m zuivel Wicky fruitzacht rood fruit |
|  |  |  | 2636 | Fruit juice dk Solevita multivit 12 fruits | Vruchtendrank Solevita multivit nectar 12 vruchten |
|  |  |  | 2637 | Fruit juice dk Roosvicee Multivit light | Vruchtendrank Roosvicee Multivit light |
|  |  |  | 2638 | Fruit juice dk ACE Fruxano/Super/Tasting Good | Vruchtendrank ACE Fruxano/Super/Tasting Good |
|  |  |  | 2639 | Smoothie fruit | Smoothie vruchten |
|  |  |  | 2640 | Smoothie fruit with dairy | Smoothie vruchten m zuivel |
|  |  |  | 2644 | Fruit drink Roosvicee 50/50 | Vruchtendrank Roosvicee 50/50 |
|  |  |  | 2663 | Juice fruit Vruchtoase multivit 12 fruits | Sap vruchten- Vruchtoase Multivitamine 12-vruchten |
|  |  |  | 2692 | Breakfast drink Ontbijt&Fit | Drinkontbijt Ontbijt&Fit |
|  |  |  | 2694 | Juice drink Sisi Frizzr tropical | Limonade vruchten- Sisi Frizzr tropical |
|  |  |  | 2716 | Juice multivitamins Albert Heijn | Sap multivitamine- Albert Heijn |
|  |  |  | 2717 | Fruit juice dk Dr.Siemer multivit light 10 fruits | Vruchtendrank Dr.Siemer multivit 10-vrnectar light |
|  |  |  | 2718 | Fruit drink Linessa Vital&Active Multivit Light nectar | Vrdrank Linessa Vital&Active Multivit Light Nectar |
|  |  |  | 2755 | Juice orange freshly squeezed | Sap sinaasappel- vers geperst |
|  |  |  | 2825 | Juice drink Dubbelfrisss less sugar | Limonade vruchten- Dubbelfrisss minder zoet |
|  |  |  | 2841 | Fruit drink Coolbest Vitaday light | Vruchtendrank Coolbest Vitaday light |
|  |  |  | 2842 | Fruit drink Coolbest Powerfruit | Vruchtendrank Coolbest Powerfruit |
|  |  |  | 2846 | Whey drink Taksi with sweetener | Weidrank Taksi m zoetstof |
|  |  |  | 2847 | Fruit drink light AH multivit red fruit | Vruchtendrank light AH multivitamine rode vruchten |
|  |  |  | 2851 | Juice multifruit mild with vit C | Sap vruchten- mild multifruit m vit C |
|  |  |  | 2854 | Fruit drink light Papaya multivitamins | Vruchtendrank light Pataya multivitamine |
|  |  |  | 2855 | Juice drink Sisi Fruitmania tropic/smooth | Limonade vruchten- Sisi Fruitmania tropical/smooth |
|  |  |  | 2856 | Fruit juice Vitafit multifruits | Sap vruchten- Vitafit multifruits |
|  |  |  | 2866 | Juice Appelsientje Superfruit pomegranate | Sap Appelsientje Superfruit granaatappel |
| **13** | **2** |  |  | **CARBONATED/SOFT/ISOTONIC DRINKS, DILUTED SYRUPS** | |
|  |  |  | 383 | Juice apple | Sap appel- |
|  |  |  | 395 | Soft drink cola with caffeine | Frisdrank cola |
|  |  |  | 400 | Soft drink without caffeine | Frisdrank |
|  |  |  | 414 | Soft drink tonic | Frisdrank tonic |
|  |  |  | 417 | Juice drink | Limonade vruchten- |
|  |  |  | 425 | Whey drink light Rivella | Weidrank Rivella light |
|  |  |  | 1294 | Whey drink Taksi with sugar | Weidrank Taksi m suiker |
|  |  |  | 1463 | Fruit juice dk minimal 2 fruits | Vruchtendrank 2 of meer vruchten |
|  |  |  | 1521 | Juice drink light | Limonade vruchten- light |
|  |  |  | 1522 | Soft drink light without caffeine | Frisdrank light z cafeine |
|  |  |  | 1523 | Soft drink light with caffeine | Frisdrank light m cafeine |
|  |  |  | 1878 | Fruit juice drink Roosvicee Multivit | Vruchtendrank Roosvicee Multivit |
|  |  |  | 2040 | Sports drink AA Sportwater | Sportdrank AA-Drink Sportwater |
|  |  |  | 2041 | Sports drink AA Isotone | Sportdrank AA-Drink Isotone |
|  |  |  | 2042 | Sports drink AA High Energy | Sportdrank AA-Drink High Energy |
|  |  |  | 2043 | Sports drink AA Multi Nine | Sportdrank AA-Drink Multi Nine |
|  |  |  | 2074 | Mineral water Bar le Duc | Bronwater Bar le Duc |
|  |  |  | 2086 | Ice tea | IJsthee |
|  |  |  | 2087 | Ice tea light | IJsthee light |
|  |  |  | 2088 | Ice tea with sugar and sweetener | IJsthee m suiker en zoetstof |
|  |  |  | 2134 | Juice drink Dubbelfrisss | Limonade vruchten- Dubbelfrisss |
|  |  |  | 2135 | Juice drink Vruchtenfris/Tintelfruit | Limonade vruchten- Vruchtenfris/Tintelfruit |
|  |  |  | 2136 | Juice drink Dubbelfrisss light | Limonade vruchten- Dubbelfrisss light |
|  |  |  | 2137 | Juice drink Vruchtenfris/Tintelfruit lig | Limonade vruchten- Vruchtenfris/Tintelfruit light |
|  |  |  | 2138 | Juice drink Spa&Fruit still | Limonade vruchten- Spa&Fruit koolzuurvrij |
|  |  |  | 2139 | Juice drink Spa&Fruit light still | Limonade vruchten- Spa&Fruit light koolzuurvrij |
|  |  |  | 2140 | Juice drink Spa&Fruit Vitamins | Limonade vruchten- Spa&Fruit Vitamine |
|  |  |  | 2141 | Mineral water with sweetener | Bronwater m zoetstof |
|  |  |  | 2153 | Fruit drink Fruit2day | Fruitdrank Fruit2day |
|  |  |  | 2218 | Sports drink Extran Energy | Sportdrank Extran Energy |
|  |  |  | 2219 | Sports drink Extran Hydro | Sportdrank Extran Hydro |
|  |  |  | 2220 | Whey drink Taksi with sugar and sweetener | Weidrank Taksi m suiker en zoetstof |
|  |  |  | 2328 | Fruit juice drink with dairy Wicky peach | Vruchtendrank m zuivel Wicky fruitzacht perzik |
|  |  |  | 2434 | Juice drink Wicky | Limonade vruchten- Wicky |
|  |  |  | 2435 | Soft drink with sugar and sweetener 2-<5 g KH | Frisdrank m suiker en zoetstof 2-<5 g KH |
|  |  |  | 2436 | Juice drink with sugar and sw | Limonade vruchten- m suiker en zoetstof |
|  |  |  | 2437 | Juice drink multivitamin Caprisonne | Limonade vruchten- multivitamine Caprisonne |
|  |  |  | 2438 | Fruit drink concentrate diluted average | Siroop vruchtenlimonade- bereid gem |
|  |  |  | 2439 | Fruit drink concentrate with sugar diluted Albert Heijn | Siroop vrlimonade- m suiker bereid blik Albert Heijn |
|  |  |  | 2440 | Fruit drink concentrate diluted 1 to 4 | Siroop vruchtenlimonade- bereid 1 op 4 |
|  |  |  | 2441 | Fruit drink concentrate diluted 1 to 7 | Siroop vruchtenlimonade- bereid 1 op 7 |
|  |  |  | 2442 | Fruit drink concentrate Lessini light diluted aver | Siroop Roosvicee Lessini light bereid gem |
|  |  |  | 2443 | Sport drink River Powerdrink/Freeway | Sportdrank River Powerdrink/Freeway |
|  |  |  | 2479 | Fruit drink concentrate Roosvicee diluted average | Siroop Roosvicee Multivit bosvr/perzik bereid gem |
|  |  |  | 2480 | Energy drink Golden Power/Bullit | Energydrink Golden Power/Bullit |
|  |  |  | 2481 | Fruit juice dk Roosvicee Spongebob/Shrek | Vruchtendrank Roosvicee Spongebob/Shrek |
|  |  |  | 2482 | Fruit drink concentrate fruitmix diluted average | Siroop vruchtenmix bereid gem |
|  |  |  | 2483 | Fruit juice drink ACE average | Vruchtendrank ACE gem |
|  |  |  | 2484 | Fruit drink concentrate light diluted average | Siroop vruchtenlimonade- light bereid gem |
|  |  |  | 2485 | Fruitdrink concentrate 10-15 mg vit C diluted average | Siroop vrlimonade- 10-15 mg vit C bereid gem |
|  |  |  | 2486 | Fruitdrink concentrate Karvan Cevitam diluted average | Siroop vrlimonade- Karvan Cevitam bereid gem |
|  |  |  | 2490 | Fruit juice drink 12 fruit nectar light | Vruchtendrank multivit 12vr nectar light |
|  |  |  | 2492 | Lemonade fruit Wicky light | Limonade vruchten- Wicky light |
|  |  |  | 2507 | Juice multifruit | Sap multivruchten- |
|  |  |  | 2508 | Fruit juice drink ACE Aldi/Kruidvat | Vruchtendrank ACE Aldi/Kruidvat |
|  |  |  | 2509 | Juice drink Sisi Fruitmania Smooth peach | Limonade vruchten- Sisi Fruitmania Smooth peach |
|  |  |  | 2515 | Fruit juice dk Surango multivit light | Vruchtendrank multivit nectar light Surango |
|  |  |  | 2564 | Juice fruit Hero Fruit&Co | Sap vruchten- Hero Fruit&Co |
|  |  |  | 2634 | Fruit juice dk minimal 2 fruits with vit C | Vruchtendrank 2 of meer vruchten m vit C |
|  |  |  | 2635 | Fruit juice drink dairy Wicky fruitzacht red fruit | Vruchtendrank m zuivel Wicky fruitzacht rood fruit |
|  |  |  | 2637 | Fruit juice dk Roosvicee Multivit light | Vruchtendrank Roosvicee Multivit light |
|  |  |  | 2638 | Fruit juice dk ACE Fruxano/Super/Tasting Good | Vruchtendrank ACE Fruxano/Super/Tasting Good |
|  |  |  | 2641 | Soft drink with sugar and sweetener 2-<5 carb with caffeine | Frisdrank m suiker en zoetst 2-<5 g KH m cafeine |
|  |  |  | 2642 | Fruit drink concentrate prepared 15-20mg vit C | Siroop vruchtenlimonade- 15-20 mg vit C bereid |
|  |  |  | 2643 | Fruit drink concentrate with sugar and sweetener diluted | Siroop vruchtenlimonade m suiker en zoetstof bereid |
|  |  |  | 2644 | Fruit drink Roosvicee 50/50 | Vruchtendrank Roosvicee 50/50 |
|  |  |  | 2645 | Sports drink Albert Heijn | Sportdrank Albert Heijn |
|  |  |  | 2646 | Sports drink Aquarius | Sportdrank Aquarius |
|  |  |  | 2664 | Lemonade fruit Sisi no bubbles Action | Limonade vruchten- Sisi no bubbles Action |
|  |  |  | 2665 | Softdrink with sugar and sweetener 5-<8g carb with caffeine | Frisdrank m suiker en zoetst 5-<8 g KH m cafeine |
|  |  |  | 2672 | Energy drink Red Bull/Euroshopper/ Rodeo | Energydrink Red Bull/Euroshopper/Rodeo |
|  |  |  | 2673 | Energy drink Red Bull sugar free | Energydrink Red Bull sugar free |
|  |  |  | 2674 | Energy drink Mxxed up | Energydrink Mxxed up |
|  |  |  | 2693 | Juice drink tea & fruit with sweetener and vit C | Limonade vruchten- tea & fruit m zoetstof en vit C |
|  |  |  | 2694 | Juice drink Sisi Frizzr tropical | Limonade vruchten- Sisi Frizzr tropical |
|  |  |  | 2695 | Fruit drink with soya Adez | Vruchtendrank m soja Adez |
|  |  |  | 2720 | Fruit drink concentrate diluted 40 mg vit C | Siroop vruchtenlimonade- m 40 mg vit C bereid |
|  |  |  | 2723 | Fruitdrink concentrate with sugar and sweetener diluted Raak | Siroop vruchtenlim m suiker en zoetst bereid Raak |
|  |  |  | 2724 | Ice tea with less sugar | IJsthee m minder suiker |
|  |  |  | 2825 | Juice drink Dubbelfrisss less sugar | Limonade vruchten- Dubbelfrisss minder zoet |
|  |  |  | 2838 | Fruit drink concentrate with sugar and sweetener diluted Tasting Good | Siroop vrlim- m suiker en zoetst bereid Tasting Good |
|  |  |  | 2842 | Fruit drink Coolbest Powerfruit | Vruchtendrank Coolbest Powerfruit |
|  |  |  | 2846 | Whey drink Taksi with sweetener | Weidrank Taksi m zoetstof |
|  |  |  | 2848 | Mineral water with sweetener and vitamins | Bronwater m zoetstof en vitamines |
|  |  |  | 2855 | Juice drink Sisi Fruitmania tropic/smooth | Limonade vruchten- Sisi Fruitmania tropical/smooth |
|  |  |  | 2857 | Sport drink Fitness | Sportdrank Fitness |
|  |  |  | 2879 | Sport drink Freeway Sportivo | Sportdrank Freeway Sportivo |
| **13** | **3** |  |  | **COFFEE, TEA AND HERBAL TEAS** |  |
| **13** | **3** | **1** |  | **COFFEE** |  |
|  |  |  | 644 | Coffee prepared | Koffie bereid |
|  |  |  | 2476 | Cappuccino freshly made | Cappuccino vers bereid |
|  |  |  | 2477 | Coffee cappuccino instant prepared | Koffie cappuccino oplos- bereid |
|  |  |  | 2478 | Cappuccino instant powder | Cappuccino oplos- poeder |
|  |  |  | 2633 | Coffee instant powder | Koffie oplos- poeder |
|  |  |  | 2647 | Coffee with sugar and milk vending machine | Koffie automaat- m suiker en melk |
|  |  |  | 2648 | Coffee with milk vending machine | Koffie automaat- m melk |
|  |  |  | 2880 | Coffee wiener melange instant powder | Koffie wiener melange oplos poeder |
|  |  |  | 2881 | Coffee wiener melange instant prepared | Koffie wiener melange oplos bereid |
| **13** | **3** | **2** |  | **TEA** |  |
|  |  |  | 645 | Tea prepared | Thee bereid |
|  |  |  | 2444 | Tea herbal instant sweetened prepared | Thee kruiden- oplos gezoet bereid |
|  |  |  | 2649 | Tea herbal instant powder | Thee kruiden- oplos- poeder |
| **13** | **3** | **3** |  | **HERBAL TEA** |  |
|  |  |  | 645 | Tea prepared | Thee bereid |
|  |  |  | 2444 | Tea herbal instant sweetened prepared | Thee kruiden- oplos gezoet bereid |
|  |  |  | 2649 | Tea herbal instant powder | Thee kruiden- oplos- poeder |
| **13** | **3** | **4** |  | **CHICORY, SUBSTITUTES** |  |
|  |  |  | 644 | Coffee prepared | Koffie bereid |
|  |  |  | 2633 | Coffee instant powder | Koffie oplos- poeder |
| **13** | **4** |  |  | **WATERS** |  |
|  |  |  | 398 | Mineral water Evian | Bronwater Evian |
|  |  |  | 406 | Mineral water Perrier | Bronwater Perrier |
|  |  |  | 411 | Mineral water Spa | Bronwater Spa |
|  |  |  | 420 | Mineral water Vittel | Bronwater Vittel |
|  |  |  | 747 | Mineral water average | Bronwater gem |
|  |  |  | 1131 | Mineral water Chaudfontaine | Bronwater Chaudfontaine |
|  |  |  | 1885 | Water average | Water gem |
|  |  |  | 1968 | Mineral water Sourcy | Bronwater Sourcy |
|  |  |  | 2074 | Mineral water Bar le Duc | Bronwater Bar le Duc |
|  |  |  |  |  |  |
| **14** |  |  |  | **ALCOHOLIC BEVERAGES** |  |
|  | 0 |  | 1466 | Liqueur >25 vol% alcohol | Likeur >25 vol% alc |
|  |  |  | 2505 | Fruit dried in brandy | Fruit gedroogd op brandewijn |
| **14** | **1** |  |  | **WINE** |  |
|  |  |  | 422 | Wine red | Wijn rode |
|  |  |  | 423 | Wine white dry | Wijn witte droge |
|  |  |  | 2142 | Wine white sweet | Wijn witte zoete |
|  |  |  | 2513 | Cider | Cider |
|  |  |  | 2610 | Wine rosé | Wijn rosé |
| **14** | **2** |  |  | **FORTIFIED WINES (SHERRY, PORTO, VERMOUTH, ...)** | |
|  |  |  | 392 | Campari | Campari |
|  |  |  | 405 | Muscatel | Muscatel |
|  |  |  | 407 | Port wine | Port |
|  |  |  | 409 | Sherry | Sherry |
|  |  |  | 415 | Vermouth | Vermouth zoete |
| **14** | **3** |  |  | **BEER, CIDER** |  |
|  |  |  | 389 | Beer brown | Bier oud bruin |
|  |  |  | 390 | Beer pilsner | Bier pils |
|  |  |  | 1468 | Beer >7 vol% alcohol | Bier zwaar >7 vol% alcohol |
|  |  |  | 2513 | Cider | Cider |
|  |  |  | 2837 | Beer with fruit flavour | Bier met vruchtensmaak |
|  |  |  | 3009 | Shandy | Shandy |
| **14** | **4** |  |  | **SPIRITS, BRANDY** |  |
|  |  |  | 391 | Brandy | Brandewijn |
|  |  |  | 394 | Cognac | Cognac |
|  |  |  | 401 | Gin young Dutch | Jenever jonge |
|  |  |  | 402 | Gin old Dutch | Jenever oude |
|  |  |  | 408 | Rum | Rum |
|  |  |  | 416 | Brandy Dutch vieux | Vieux |
|  |  |  | 421 | Whisky | Whisky |
|  |  |  | 805 | Jägermeister herb liquor | Jägermeister |
|  |  |  | 1134 | Beerenburg herb liquor | Beerenburg |
|  |  |  | 1466 | Liqueur >25 vol% alcohol | Likeur >25 vol% alc |
| **14** | **5** |  |  | **ANISEED DRINKS (PASTIS,..)** |  |
|  |  |  | 421 | Whisky | Whisky |
| **14** | **6** |  |  | **LIQUEURS** |  |
|  |  |  | 382 | Advocaat liqueur | Advocaat |
|  |  |  | 386 | Gin Dutch red currant flavoured | Jenever bessen- |
|  |  |  | 392 | Campari | Campari |
|  |  |  | 403 | Liqueur 15-25 vol% alcohol | Likeur 15-25 vol% alc |
|  |  |  | 421 | Whisky | Whisky |
|  |  |  | 1134 | Beerenburg herb liquor | Beerenburg |
|  |  |  | 1465 | Liqueur <15 vol% alcohol | Likeur <15 vol% alc |
|  |  |  | 1466 | Liqueur >25 vol% alcohol | Likeur >25 vol% alc |
|  |  |  | 2828 | Liqueur with cream 15-25 vol% alcohol | Likeur met room 15-25 vol% alc |
| **14** | **7** |  |  | **COCKTAILS, PUNCHES** |  |
|  |  |  | 386 | Gin Dutch red currant flavoured | Jenever bessen- |
|  |  |  | 403 | Liqueur 15-25 vol% alcohol | Likeur 15-25 vol% alc |
|  |  |  | 408 | Rum | Rum |
|  |  |  | 2311 | Breezer | Breezer |
|  |  |  |  |  |  |
| **15** |  |  |  | **CONDIMENTS AND SAUCES** |  |
| **15** | **1** | **0** |  | **SAUCES** |  |
|  |  |  | 454 | Piccalilly | Piccalilly |
|  |  |  | 548 | Sauce tomato based shashlik | Saus schaschlik- |
|  |  |  | 584 | Ketchup curry | Ketchup curry- |
|  |  |  | 601 | Oil olive | Olie olijf- |
|  |  |  | 612 | Sauce tomato based with 15% oil | Saus frikandel- 15% olie |
|  |  |  | 616 | Peanut sauce jar prepared | Saus saté- op basis potje bereid |
|  |  |  | 1213 | Soya sauce salt | Ketjap zout |
|  |  |  | 1215 | Soya sauce sweet | Ketjap zoet |
|  |  |  | 1515 | Sauce based on roux prepared | Saus op basis roux bereid |
|  |  |  | 1517 | Sauce mix packet <3% fat prepared | Saus op basis pakje <3% vet bereid |
|  |  |  | 1518 | Sauce mix packet >3% fat prepared | Saus op basis pakje >3% vet bereid |
|  |  |  | 1524 | Sauce tomato ready made jar | Saus tomaten- kant-en-klaar glas |
|  |  |  | 1803 | Sauce oriental ready-made in jar/bag | Saus oosterse kant-en-klaar glas/zak |
|  |  |  | 1847 | Margarine product tub Becel Dieet | Margarineproduct kuipje Becel Dieet |
|  |  |  | 1886 | Mango chutney | Mango chutney |
|  |  |  | 1938 | Peanut sauce packet prepared | Saus saté- op basis pakje bereid |
|  |  |  | 2063 | Margarine 80% fat >24 g saturates | Margarine 80% vet >24 g verz vetz |
|  |  |  | 2066 | Cooking fat liquid 97% fat <17 g sat | Bak- en braadvet vloeib 97% vet <17g verz vetz |
|  |  |  | 2067 | Cooking fat solid 97% fat >17 g sat | Bak- en braadvet vast 97% vet >17 g verz vetz |
|  |  |  | 2077 | Margarine liq 80% fat <17 g saturates | Margarine vloeibaar 80% vet <17 g verz vetz |
|  |  |  | 2178 | Pesto | Pesto |
|  |  |  | 2418 | Sauce hot liquid ready made <12% fat | Saus warm vloeibaar kant-en-klaar <12% vet |
|  |  |  | 2419 | Sauce cheese- | Saus kaas- op basis roux bereid |
|  |  |  | 2420 | Sauce hot liquid ready made >12% fat | Saus warm vloeibaar kant-en-klaar >12% vet |
|  |  |  | 2421 | Sauce Chicken Tonight low fat varieties | Saus Chicken Tonight div. smaken laag vetgehalte |
|  |  |  | 2445 | Sauce butter- | Saus boter- |
|  |  |  | 2447 | Sauce veg with semi-skimmed milk and maize | Saus groente- van halfvolle melk en maizena |
|  |  |  | 2448 | Sauce powder approx 30% fat | Sauspoeder ca 30%vet |
|  |  |  | 2449 | Gravy 25% fat clear prepared with gravy powder | Jus 25% vet ongebonden bereid m juspoeder |
|  |  |  | 2450 | Gravy 50% fat prepared with gravy powder | Jus 50% vet ongebonden bereid m juspoeder |
|  |  |  | 2451 | Gravy 25% fat thickened prepared with gravy powder | Jus 25% vet gebonden bereid m juspoeder |
|  |  |  | 2452 | Gravy 25% fat thick prepared without gravy powder | Jus 25% vet gebonden bereid z juspoeder |
|  |  |  | 2453 | Gravy 50% fat thick prepared without gravy powder | Jus 50% vet gebonden bereid z juspoeder |
|  |  |  | 2454 | Gravy 75% fat thick prepared without gravy powder | Jus 75% vet gebonden z juspoeder |
|  |  |  | 2455 | Sauce Aardappel Anders average | Saus Aardappel Anders gem |
|  |  |  | 2456 | Gravy 5% fat thick prepared without gravy powder | Jus 5% vet gebonden bereid z juspoeder |
|  |  |  | 2457 | Peanut sauce homemade with semi-skimmed milk without fat | Saus saté huishoudelijk bereid m hv melk z vet |
|  |  |  | 2458 | Peanut sauce homemade with water without fat | Saus saté huishoudelijk bereid m water z vet |
|  |  |  | 2459 | Gravy 50% fat clear prepared without gravy powder | Jus 50% vet ongebonden bereid z juspoeder |
|  |  |  | 2460 | Gravy 75% fat clear prepared without gravy powder | Jus 75% vet ongebonden bereid z juspoeder |
|  |  |  | 2461 | Gravy 50%fat thickened prepared with gravy powder | Jus 50% vet gebonden bereid m juspoeder |
|  |  |  | 2462 | Gravy 5% fat clear prepared without gravy powder | Jus 5% vet ongebonden bereid z juspoeder |
|  |  |  | 2463 | Sauce worcester- | Saus worcester- |
|  |  |  | 2464 | Sauce powder approx 10% fat | Sauspoeder ca 10%vet |
|  |  |  | 2465 | Salad dressing Yofresh | Dressing sla- Yofresh |
|  |  |  | 2466 | Salad dressing vinaigrette | Dressing vinaigrette- |
|  |  |  | 2468 | Salad dressing honey/mustard | Dressing honing/mosterd- |
|  |  |  | 2469 | Sauce mayonnaise based average | Saus mayonaisebasis gem |
|  |  |  | 2526 | Gravy 25% fat thickened (with cooking fat 2067) with gravy powder | Jus 25% vet gebonden (m bak en brvet 2067) m juspoeder |
|  |  |  | 2580 | Gravy 5% fat thickened with gravy powder | Jus 5% vet gebonden bereid m juspoeder |
|  |  |  | 2584 | Sauce prepared with semi-skimmed milk en marg 2063/2062 | Saus bereid m hv melk en marg 2063/2062 |
|  |  |  | 2585 | Gravy 25% fat with cooking fat 2067 | Jus 25% vet (m bak- en braadvet 2067) |
|  |  |  | 2586 | Gravy 25% fat with margarine 2063 | Jus 25% vet (m margarine 2063) |
|  |  |  | 2587 | Gravy 25% fat with margarine liq 2077 | Jus 25% vet (m maragrine vloeibaar 2077) |
|  |  |  | 2588 | Gravy 25% fat clear prepared without gravy powder | Jus 25% vet ongebonden bereid z juspoeder |
|  |  |  | 2589 | Gravy 25% fat with olive oil | Jus 25% vet (m olijfolie) |
|  |  |  | 2590 | Gravy 25% fat with butter | Jus 25% vet (m boter) |
|  |  |  | 2591 | Gravy 25% fat with blue band finesse v koken | Jus 25% vet (m blue band finesse v koken) |
|  |  |  | 2592 | Gravy 50% fat with cooking fat 2067 | Jus 50% vet (m bak- en braadvet 2067) |
|  |  |  | 2593 | Gravy 50% fat with margarine 2063 | Jus 50% vet (m margarine 2063) |
|  |  |  | 2594 | Gravy 50% fat with margarine liq 2077 | Jus 50% vet (m margarine vloeibaar 2077) |
|  |  |  | 2596 | Gravy 75% fat with margarine 2063 | Jus 75% vet (m margarine 2063) |
|  |  |  | 2598 | Gravy 75% fat with cooking fat 2067 | Jus 75% vet (m bak- en braadvet 2067) |
|  |  |  | 2599 | Gravy 75% fat with margarine liq 2077 | Jus 75% vet (m margarine vloeibaar 2077) |
|  |  |  | 2600 | Gravy 25% fat thickened with marg 2063 | Jus 25% vet gebonden (m margarine 2063) |
|  |  |  | 2601 | Gravy 25% fat thickened with cook fat 2067 | Jus 25% vet gebonden (m bak- en braadvet 2067) |
|  |  |  | 2602 | Gravy 25% fat thickened with olive oil | Jus 25% vet gebonden (m olijfolie) |
|  |  |  | 2603 | Gravy 25% fat with marg 2063 and gravy powder | Jus 25% vet gebonden (m margarine 2063) m juspoeder |
|  |  |  | 2606 | Mix for marinade powder unprepared | Mix voor marinade poeder onbereid |
|  |  |  | 2611 | Sauce Joppie | Saus Joppie- |
|  |  |  | 2612 | Tapenade olive | Tapenade van olijven |
|  |  |  | 2613 | Mix seasoning Mexican unprepared | Mix seasoning Mexicaans onbereid |
|  |  |  | 2623 | Sauce Chicken Tonight high fat varieties | Saus Chicken Tonight div. smaken hoog vetgehalte |
|  |  |  | 2624 | Gravy 25% fat with margarine liquid 2558 | Jus 25% vet (m margarine vloeibaar 2558) |
|  |  |  | 2625 | Gravy 25% fat with cooking fat 2563 | Jus 25% vet (m bak- en braadvet 2563) |
|  |  |  | 2626 | Gravy 25% fat with cooking fat liq 2562 | Jus 25% vet (m bak- en braadvet vloeibaar 2562) |
|  |  |  | 2627 | Gravy 50% fat with margarine liquid 2558 | Jus 50% vet (m margarine vloeibaar 2558) |
|  |  |  | 2628 | Gravy 50% fat with cooking fat liquid 2562 | Jus 50% vet (m bak- en braadvet vloeibaar 2562) |
|  |  |  | 2629 | Gravy 50% fat with cooking fat 2563 | Jus 50% vet (m bak- en braadvet 2563) |
|  |  |  | 2630 | Gravy 50% fat with olive oil | Jus 50% vet (m olijfolie) |
|  |  |  | 2631 | Gravy 75% fat with olive oil | Jus 75% vet (m olijfolie) |
|  |  |  | 2632 | Gravy 75% fat with cooking fat 2563 | Jus 75% vet (m bak- en braadvet 2563) |
|  |  |  | 2650 | Gravy no fat prepared with gravy powder | Jus zonder vet bereid m juspoeder |
|  |  |  | 2651 | Sauce cocktail/party/table >25% oil | Saus cocktail/party/tafel- >25% olie |
|  |  |  | 2669 | Mix Maggi oven-/dagschotels prepared | Mix voor oven-/dagschotels Maggi bereid |
|  |  |  | 2670 | Mix Hollands Pannetje Maggi unprepared | Mix voor Hollands Pannetje Maggi onbereid |
|  |  |  | 2713 | Margarine product liquid light | Margarineproduct vloeibaar light |
|  |  |  | 2833 | Gravy 50% fat with cooking fat 2067 and gravy powder | Jus 50% vet gebonden (m bak&brvet 2067) m juspoeder |
|  |  |  | 2871 | Gravy 25% fat with marg 2077 and gravy powder | Jus 25% vet geb (m marg vloeib 2077) m juspoeder |
|  |  |  | 2872 | Gravy 25% fat with marg 2558 and gravy powder | Jus 25% vet gebonden (m marg vloeibaar 2558) |
|  |  |  | 3036 | Gravy 75%fat thick prepared with gravy powder | Jus 75% vet gebonden bereid m juspoeder |
|  |  |  | 3037 | Gravy 75% fat clear prepared with gravy powder | Jus 75% vet ongebonden bereid m juspoeder |
| **15** | **1** | **1** |  | **TOMATO SAUCES** |  |
|  |  |  | 428 | Sauce barbecue | Saus barbecue- |
|  |  |  | 462 | Ketchup tomato | Ketchup tomaten- |
|  |  |  | 548 | Sauce tomato based shashlik | Saus schaschlik- |
|  |  |  | 583 | Ketchup hot | Ketchup hot |
|  |  |  | 584 | Ketchup curry | Ketchup curry- |
|  |  |  | 1232 | Pepper red hot paste | Sambal oelek |
|  |  |  | 1517 | Sauce mix packet <3% fat prepared | Saus op basis pakje <3% vet bereid |
|  |  |  | 1518 | Sauce mix packet >3% fat prepared | Saus op basis pakje >3% vet bereid |
|  |  |  | 1524 | Sauce tomato ready made jar | Saus tomaten- kant-en-klaar glas |
|  |  |  | 1803 | Sauce oriental ready-made in jar/bag | Saus oosterse kant-en-klaar glas/zak |
|  |  |  | 2178 | Pesto | Pesto |
|  |  |  | 2418 | Sauce hot liquid ready made <12% fat | Saus warm vloeibaar kant-en-klaar <12% vet |
|  |  |  | 2448 | Sauce powder approx 30% fat | Sauspoeder ca 30%vet |
|  |  |  | 2464 | Sauce powder approx 10% fat | Sauspoeder ca 10%vet |
|  |  |  | 2614 | Ketchup salsa | Ketchup salsa |
|  |  |  | 2623 | Sauce Chicken Tonight high fat varieties | Saus Chicken Tonight div. smaken hoog vetgehalte |
| **15** | **1** | **2** |  | **DRESSING SAUCES** |  |
|  |  |  | 458 | Salad cream 25% oil | Saus sla- 25% olie |
|  |  |  | 459 | Salad cream 50% oil Calve | Saus sla- 50% olie Calve |
|  |  |  | 465 | Sauce for chips 25% oil | Saus frites- 25% olie |
|  |  |  | 466 | Sauce for chips 35% oil | Saus frites- 35% olie |
|  |  |  | 553 | Salad dressing 40% oil Becel | Dressing 40% olie Becel |
|  |  |  | 729 | Mayonnaise low fat 40% oil | Halvanaise |
|  |  |  | 844 | Salad dressing naturel without oil | Dressing naturel z olie |
|  |  |  | 1260 | Mayonnaise yoghurt based 25% oil | Yoghonaise |
|  |  |  | 2465 | Salad dressing Yofresh | Dressing sla- Yofresh |
|  |  |  | 2466 | Salad dressing vinaigrette | Dressing vinaigrette- |
|  |  |  | 2467 | Salad dressing./sauce approx 13% oil | Dressing/saus frites-/saus sla- ca 13% olie |
|  |  |  | 2468 | Salad dressing honey/mustard | Dressing honing/mosterd- |
|  |  |  | 2470 | Sauce for chips 5% oil | Saus frites- 5% olie |
|  |  |  | 2573 | Sauce garlic 20% oil | Saus knoflook- 20% olie |
|  |  |  | 2605 | Salad dressing olive oil-vinegar | Dressing olijfolie-azijn |
|  |  |  | 2611 | Sauce Joppie | Saus Joppie- |
|  |  |  | 2667 | Salad dressing 20% oil with yoghurt | Dressing sla- 20% olie m yoghurt |
| **15** | **1** | **3** |  | **MAYONNAISES AND SIMILARS** |  |
|  |  |  | 437 | Sauce cocktail/party/table 25% oil | Saus cocktail/party/tafel- 25% olie |
|  |  |  | 451 | Mayonnaise | Mayonaise |
|  |  |  | 458 | Salad cream 25% oil | Saus sla- 25% olie |
|  |  |  | 465 | Sauce for chips 25% oil | Saus frites- 25% olie |
|  |  |  | 466 | Sauce for chips 35% oil | Saus frites- 35% olie |
|  |  |  | 549 | Sauce curry 25% oil | Saus kerrie- 25% olie |
|  |  |  | 574 | Sandwich spread cucumber | Spread sandwich- komkommer |
|  |  |  | 575 | Sandwich spread original | Spread sandwich- naturel |
|  |  |  | 584 | Ketchup curry | Ketchup curry- |
|  |  |  | 612 | Sauce tomato based with 15% oil | Saus frikandel- 15% olie |
|  |  |  | 729 | Mayonnaise low fat 40% oil | Halvanaise |
|  |  |  | 1260 | Mayonnaise yoghurt based 25% oil | Yoghonaise |
|  |  |  | 1496 | Salad fish | Salade vis- lunch/borrel |
|  |  |  | 2083 | Mayonnaise product with olive oil | Mayonaiseproduct m olijfolie |
|  |  |  | 2465 | Salad dressing Yofresh | Dressing sla- Yofresh |
|  |  |  | 2467 | Salad dressing/sauce for chips approx 13% oil | Dressing/saus frites-/saus sla- ca 13% olie |
|  |  |  | 2469 | Sauce mayonnaise based average | Saus mayonaisebasis gem |
|  |  |  | 2470 | Sauce for chips 5% oil | Saus frites- 5% olie |
|  |  |  | 2471 | Mayonnaise product approx 35% oil | Mayonaiseproduct ca 35% olie |
|  |  |  | 2573 | Sauce garlic 20% oil | Saus knoflook- 20% olie |
|  |  |  | 2611 | Sauce Joppie | Saus Joppie- |
|  |  |  | 2651 | Sauce cocktail/party/table >25% oil | Saus cocktail/party/tafel- >25% olie |
| **15** | **1** | **4** |  | **DESSERT SAUCES** |  |
|  |  |  | 539 | Sauce fruit for pudding | Dessertsaus vruchten- |
|  |  |  | 540 | Sauce chocolate for pudding | Dessertsaus chocolade- |
|  |  |  | 2873 | Saus caramel for pudding | Dessertsaus caramel- |
| **15** | **2** |  |  | **YEAST** |  |
|  |  |  | 441 | Yeast extract Marmite | Gistextract Marmite |
|  |  |  | 1025 | Yeast dried | Gist gedroogd |
| **15** | **3** |  |  | **SPICES, HERBS AND FLAVOURINGS** |  |
| **15** | **4** |  |  | **CONDIMENTS** |  |
|  |  |  | 439 | Gelatin | Gelatine |
|  |  |  | 704 | Shrimp paste trassie | Garnalenpasta trassie |
|  |  |  | 824 | Mustard | Mosterd |
|  |  |  | 1232 | Pepper red hot paste | Sambal oelek |
|  |  |  | 1500 | Herb mix cube | Kruidenmix blok |
|  |  |  | 2464 | Sauce powder approx 10% fat | Sauspoeder ca 10%vet |
|  |  |  | 2473 | Herb paste boemboe | Pasta kruiden-/boemboe |
|  |  |  | 2613 | Mix seasoning Mexican unprepared | Mix seasoning Mexicaans onbereid |
|  |  |  | 2615 | Vinegar | Azijn |
|  |  |  | 2668 | Mix Maggi oven/dagschotel unprepared | Mix voor oven-/dagschotels Maggi onbereid |
|  |  |  | 2669 | Mix Maggi oven-/dagschotel prepared | Mix voor oven-/dagschotels Maggi bereid |
|  |  |  | 2670 | Mix Hollands Pannetje Maggi unprepared | Mix voor Hollands Pannetje Maggi onbereid |
|  |  |  | 2671 | Mix rice/Chinese noodles unprepared | Mix voor nasi/bami onbereid |
|  |  |  | 2681 | Mixed spices Wereldgerechten unprep | Mix kruiden- Wereldgerechten onbereid |
|  |  |  | 2874 | Mix rice/Chinese noodles prepared | Mix voor nasi/bami bereid |
|  |  |  |  |  |  |
| **16** |  |  |  | **SOUPS, BOUILLON** |  |
| **16** | **1** |  |  | **SOUPS** |  |
|  |  |  | 757 | Soup clear with vegetables and noodles | Soep heldere m vermicelli en groente |
|  |  |  | 758 | Soup clear with meat | Soep heldere m vlees |
|  |  |  | 759 | Soup clear with vegetables | Soep heldere m groente |
|  |  |  | 760 | Soup clear with meat and noodles | Soep heldere m vermicelli en vlees |
|  |  |  | 761 | Soup clear with meat and vegetables | Soep heldere m vlees en groente |
|  |  |  | 762 | Soup clear with meat vegetables and noodles | Soep heldere m vlees/vermicelli/groente |
|  |  |  | 763 | Soup thickened with vegetables | Soep gebonden m groente |
|  |  |  | 764 | Soup thickened with meat | Soep gebonden m vlees |
|  |  |  | 765 | Soup main course with legumes without meat | Soep maaltijd- m peulvruchten z vlees |
|  |  |  | 766 | Soup main course with legumes and meat | Soep maaltijd- m peulvruchten en vlees |
|  |  |  | 791 | Soup clear with noodles | Soep heldere m vermicelli |
|  |  |  | 792 | Soup thickened with meat and vegetables | Soep gebonden m vlees en groente |
|  |  |  | 797 | Soup vegetable based dried packet prep | Soep op groentebasis bereid pakje |
|  |  |  | 798 | Soup meat based dried packet prepared | Soep op vleesbasis bereid pakje |
|  |  |  | 799 | Soup legume based dried packet prepared | Soep op peulvruchtenbasis bereid pakje |
|  |  |  | 800 | Soup vegetable based tinned prepared | Soep op groentebasis bereid blik/zak |
|  |  |  | 801 | Soup meat based tinned prepared | Soep op vleesbasis bereid blik/zak |
|  |  |  | 802 | Soup legume based tinned prepared | Soep op peulvruchtenbasis bereid blik |
|  |  |  | 803 | Soup main course tinned prepared | Soep maaltijd- bereid blik |
|  |  |  | 2487 | Soup veg and meat based tinned prep | Soep op groente- en vleesbasis bereid blik |
|  |  |  | 2488 | Soup vegetable and meat based prepared pack | Soep op groente- en vleesbasis bereid pakje |
|  |  |  | 2540 | Soup noodle prepared | Soep noodle- bereid |
|  |  |  | 2561 | Soup thickened no filling | Soep gebonden z vulling |
|  |  |  | 2932 | Soup cup-a-soup prepared | Soep cup-a-soup bereid |
| **16** | **2** |  |  | **BOUILLON** |  |
|  |  |  | 1528 | Stock/bouillon from cube prepared | Bouillon van blokje bereid |
|  |  |  |  |  |  |
| **17** | **0** |  |  | **MISCELLANEOUS** |  |
|  |  |  | 2030 | Quorn minced unprepared | Quorn fijngehakt onbereid |
|  |  |  | 2031 | Quorn pieces unprepared | Quorn stukjes onbereid |
|  |  |  | 2032 | Quorn fillet crumbed unprepared | Quorn filet gepaneerd onbereid |
|  |  |  | 2281 | Vegetarian product without brdcrumbs Valess | Valess filet ongepaneerd onbereid |
|  |  |  | 2282 | Vegetarian schnitzel Valess unprepared | Valess schnitzel onbereid |
|  |  |  | 2286 | Vegetable burger vegetarian unpr | Schijf vegetarisch onbereid |
|  |  |  | 2994 | Falafel unprepared | Falafel onbereid |
|  |  |  | 3040 | Vegetarian prod with cheese Valess unprep | Valess kaasvariatie onbereid |
|  |  |  | 3041 | Vegetarian product filled filet Valess | Valess gevulde filet onbereid |
| **17** | **1** |  |  | **SOYA PRODUCTS** |  |
|  |  |  | 263 | Biscuit brown/wholemeal | Biscuit tarwe-/volkoren- |
|  |  |  | 687 | Tahoe soya curd | Tahoe |
|  |  |  | 688 | Tempeh fermented soya beans | Tempe |
|  |  |  | 869 | Flour soya full fat | Meel soja- volvet |
|  |  |  | 870 | Milk soya average | Melk soja- gem |
|  |  |  | 871 | Miso soya paste | Sojapasta miso |
|  |  |  | 971 | Beans soya boiled | Bonen soja- gekookt |
|  |  |  | 1094 | Vegetable paste Tartex | Pasta plantaardig Tartex |
|  |  |  | 1380 | Dessert soya Alpro | Dessert soja- Alpro |
|  |  |  | 1381 | Milk soya Natural Fresh Alpro | Melk soja- Natural Fresh Alpro |
|  |  |  | 1510 | Milk soya Nature Ca+ Alpro | Melk soja- Nature Ca+ Alpro |
|  |  |  | 1511 | Hamburger vegetarian unprep | Hamburger vegetarisch onbereid |
|  |  |  | 1512 | Schnitzel vegetarian unprep | Schnitzel vegetarisch onbereid |
|  |  |  | 1602 | Milk soya several flavours Alpro | Melk soja- diverse smaken Alpro |
|  |  |  | 1953 | Soya based yoghurt Yofu soja Alpro fruit/vanilla | Yofu soja Alpro m vruchten/vanille |
|  |  |  | 2046 | Vegetarian mincemeat balls unprep | Balletjes vegetarisch onbereid |
|  |  |  | 2047 | Mincemeat vegetarian unprepared | Gehakt fijn- vegetarisch onbereid |
|  |  |  | 2285 | Vegetarian sausages unprepared | Worst vegetarisch onbereid |
|  |  |  | 2286 | Vegetable burger unprepared | Schijf vegetarisch onbereid |
|  |  |  | 2541 | Sausage luncheon meat vegetarian | Worst boterham- vegetarisch |
|  |  |  | 2542 | Paté vegetarian | Paté/smeerleverworst vegetarisch |
|  |  |  | 2543 | Crisps based on soya Socrispy | Chips op sojabasis Socrispy |
|  |  |  | 2544 | Ham vegetarian | Ham vegetarisch |
|  |  |  | 2616 | Burger vegetarian cheese unprepared | Burger vegetarisch met kaas onbereid |
|  |  |  | 2858 | Milk soya natural Fresh light Alpro | Melk soja- Natural Fresh light Alpro |
|  |  |  | 2951 | Nuggets vegetarian unprepared | Nuggets vegetarisch onbereid |
| **17** | **2** | **0** |  | **DIETETIC PRODUCTS** |  |
|  |  |  | 76 | Modular protein powder Protifar Plus Nutricia | Protifar Plus poeder Nutricia |
|  |  |  | 1048 | Polymeric drink Nutridrink Protein p 100 ml Nutricia | Nutridrink Protein p 100 ml Nutricia |
|  |  |  | 1246 | Polymeric drink Nutridrink p 100 ml Nutricia | Nutridrink p 100 ml Nutricia |
|  |  |  | 1279 | Infant food meal 15 months | Kindervoeding maaltijd 15 mnd |
|  |  |  | 1702 | Polymeric drink Nutridrink Multi Fibre per 100 ml Nutricia | Nutridrink Multi Fibre p 100 ml Nutricia |
|  |  |  | 1704 | Pef Fortify Complete powder Nutricia | Fortify Complete poeder Nutricia |
|  |  |  | 1970 | Milk chocolate-flavoured Chocomel light | Melk chocolade- m zoetstof Chocomel light |
|  |  |  | 1997 | Preparation Respifor p 100 ml Nutricia | Respifor p 100 ml Nutricia |
|  |  |  | 2054 | Infant food meal 12 months | Babyvoeding maaltijd 12 mnd |
|  |  |  | 2545 | Meal replacer milkshake prepared semi-skimmed milk Herbalife | Maaltijdverv milkshake bereid m hv melk Herbalife |
|  |  |  | 2546 | Meal replacer milkshake powder Herbalife | Maaltijdvervanger milkshake poeder Herbalife |
|  |  |  | 2839 | Meal replacer Cambridge shake/soup prep | Cambridge maaltijdvervanger shake/soep bereid |
|  |  |  | 2861 | Prodimed powder average | Prodimed eiwitpreparaat poeder gem |
|  |  |  | 2882 | Meal replacer bar Weightcare | Maaltijdreep Weightcare |
| **17** | **2** | **1** |  | **ARTIFICIAL SWEETENERS** |  |
|  |  |  | 1088 | Sweetener p tablet Natrena | Zoetstof Natrena p tablet |
|  |  |  | 1089 | Sweetener liquid p drop Natrena | Zoetstof Natrena p druppel |
|  |  |  | 1591 | Sweetener aspartame per tablet | Zoetstof op aspartaambasis p tablet |
|  |  |  | 1592 | Sweetener saccharine per tablet | Zoetstof op sacharinebasis p tablet |
|  |  |  | 1593 | Sweetener aspartame/acesulfame p tablet | Zoetstof aspartaam en acesulfaam p tablet |
|  |  |  | 1594 | Sweetener saccharine/cyclamate p tablet | Zoetstof sacharine en cyclamaat p tablet |
|  |  |  | 1596 | Sweetener aspartame powder per tsp | Zoetstof op aspartaambasis p theelepel |
|  |  |  | 1597 | Sweetener aspartame/acesulfame per tsp | Zoetstof aspartaam en acesulfaam p theelepel |
|  |  |  | 1598 | Sweetener saccharine powder per tsp | Zoetstof op sacharinebasis p theelepel |
|  |  |  | 2697 | Sugar light | Suiker light |
| **17** | **3** |  |  | **SNACKS** |  |
|  |  |  | 266 | Snack sausage roll puff pastry | Broodje saucijzen- |
|  |  |  | 326 | Croquette meat ragout deep-fried | Kroket bereid |
|  |  |  | 369 | Spring roll fried | Loempia bereid |
|  |  |  | 467 | Pancake | Pannenkoek |
|  |  |  | 610 | Chinese noodle ball deep-fried | Bamibal bereid |
|  |  |  | 892 | Pizza mini frozen | Pizza mini diepvries |
|  |  |  | 901 | Snack sausage roll with bread dough pastry | Broodje worsten- |
|  |  |  | 943 | Croquette meat Bitterbal prepared in oven | Bitterbal bereid in oven |
|  |  |  | 944 | Croquette meat ragout prepared in oven | Kroket bereid in oven |
|  |  |  | 1488 | Pastry puff cheese filled unprepared | Kaassouffle onbereid |
|  |  |  | 1802 | Crispbread sandwich Wasa | Knäckebröd sandwich Wasa |
|  |  |  | 2334 | Minced meat beef/pork raw with egg/brcrumbs | Gehakt hoh m ei en paneermeel rauw |
|  |  |  | 2362 | Sausage Dutch Frikandel frozen unprep | Frikandel diepvries onbereid |
|  |  |  | 2365 | Chicken filled with ham and cheese raw | Kip cordon bleu rauw |
|  |  |  | 2547 | Chinese noodle ball frozen unprepared | Bamibal diepvries onbereid |
|  |  |  | 2548 | Croquette meat ragout frozen unprepared | Kroket diepvries onbereid |
|  |  |  | 2549 | Springroll frozen unprepared | Loempia diepvries onbereid |
|  |  |  | 2550 | Wrap shoarma roll prepared without fat | Shoarmarol bereid (z bereidingsvet) |
|  |  |  | 2551 | Croissant with ham and cheese | Croissant m ham en kaas |
|  |  |  | 2552 | Chicken sticks breaded frozen unprepared | Kipcorn diepvries onbereid |
|  |  |  | 2553 | Cheese pasty with puff pastry | Broodje kaas- bladerdeeg |
|  |  |  | 2554 | Roll white with cheese | Broodje wit- belegd m kaas |
|  |  |  | 2555 | Viandel unprepared Mora | Viandel onbereid Mora |
|  |  |  | 2617 | Bread stuffed Bapao meat | Broodje bapao vlees |
|  |  |  | 2618 | Bread stuffed Bapao vegetarian mince | Broodje bapao vegetarisch gehakt |
|  |  |  | 2619 | Meatball Berenklauw unprepared | Berenklauw onbereid |
|  |  |  | 2830 | Croissant with cheese | Croissant met kaas |
|  |  |  | 2859 | Pancake with cheese | Pannenkoek met kaas |
|  |  |  | 2860 | Ragout pasty with puff pastry | Broodje ragout- bladerdeegbasis |
|  |  |  |  |  |  |
